# Non-Covalent Inhibitors of SARS-CoV-2 Papain-Like Protease (PLpro): In Vitro and In Vivo Antiviral Activity

**DOI:** 10.1021/acs.jmedchem.4c00378

**Published:** 2024-08-05

**Authors:** Ganga Reddy Velma, Zhengnan Shen, Cameron Holberg, Jiqiang Fu, Farinaz Soleymani, Laura Cooper, Omar Lozano Ramos, Divakar Indukuri, Soumya Reddy Musku, Pavel Rychetsky, Steve Slilaty, Zuomei Li, Kiira Ratia, Lijun Rong, Dominik Schenten, Rui Xiong, Gregory R. J Thatcher

**Affiliations:** † Department of Pharmacology & Toxicology, R. Ken Coit College of Pharmacy, University of Arizona, Tucson 85721, Arizona, United States; ‡ Department of Chemistry & Biochemistry, Colleges of Science and Medicine, University of Arizona, Tucson 85721, Arizona, United States; § Department of Microbiology, College of Medicine, University of Illinois at Chicago (UIC), Chicago 60612, Illinois, United States; ∥ Sunshine Biopharma Inc, 333 Las Olas Way, CU4 Suite 433, Fort Lauderdale 33301, Florida, United States; ⊥ Research Resources Center, University of Illinois at Chicago (UIC), Chicago 60612, Illinois, United States; # Department of Immunology, College of Medicine, University of Arizona, Tucson 85721, Arizona, United States

## Abstract

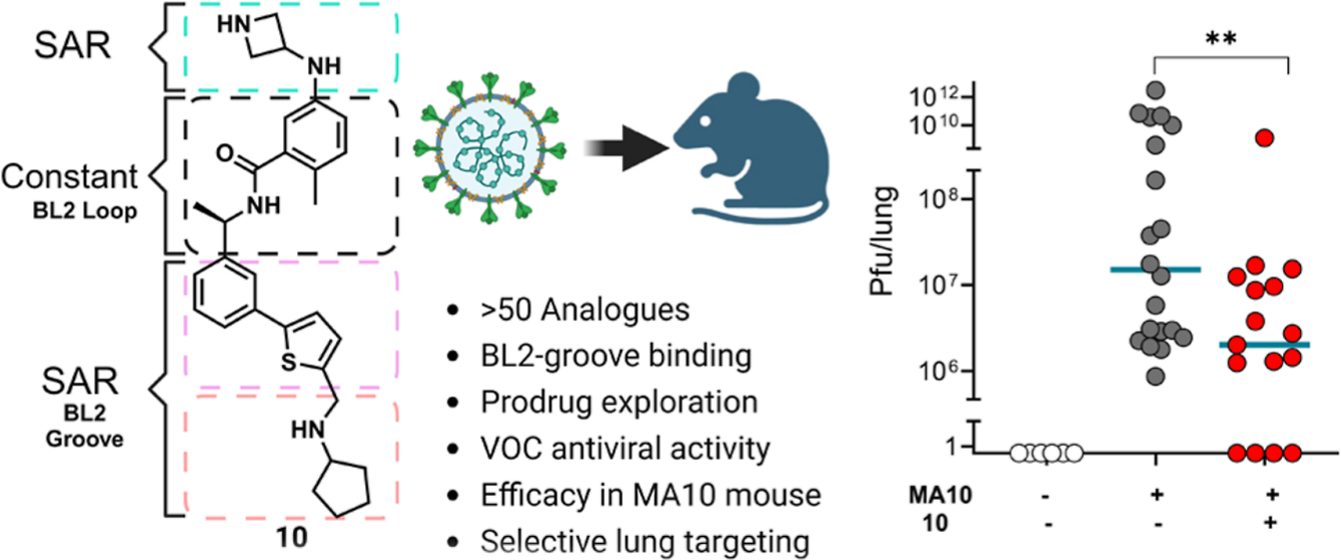

The SARS-CoV-2 papain-like protease (PLpro), essential for viral processing and immune response disruption, is a promising target for treating acute infection of SARS-CoV-2. To date, there have been no reports of PLpro inhibitors with both submicromolar potency and animal model efficacy. To address the challenge of PLpro’s featureless active site, a noncovalent inhibitor library with over 50 new analogs was developed, targeting the PLpro active site by modulating the BL2-loop and engaging the BL2-groove. Notably, compounds **42** and **10** exhibited strong antiviral effects and were further analyzed pharmacokinetically. **10**, in particular, showed a significant lung accumulation, up to 12.9-fold greater than plasma exposure, and was effective in a mouse model of SARS-CoV-2 infection, as well as against several SARS-CoV-2 variants. These findings highlight the potential of **10** as an in vivo chemical probe for studying PLpro inhibition in SARS-CoV-2 infection.

## Introduction

The COVID-19 pandemic, caused by the novel severe acute respiratory syndrome coronavirus 2 (SARS-CoV-2), has caused profound socioeconomic challenges for humankind.^1−4^ The rapid development of vaccines has greatly accelerated the buildup of anti-COVID immunity in the general population, helping to shift the pandemic toward an endemic state. However, COVID-19 mutates rapidly, with a mutation rate estimated at around 1 × 10^–6^–2 × 10^–6^ mutations per nucleotide per replication cycle. This rate allows the virus to acquire nearly two evolutionary changes per month.^5^ Consequently, the emergence of variants of concern (VOC) and potential future antigenically distinct lineages continue to pose a significant threat to public health. As such, continued basic and translational research is essential for active control of these unpredictable and potentially dangerous VOC.

In contrast to biologics, progress on small molecule antivirals for SARS-CoV-2 has been disappointing. Only three small molecule antiviral drugs are either fully approved or approved for emergency use authorization (EUA) by the U.S. Food and Drug Administration (FDA). Two of these agents, remdesivir (Veklury), and molnupiravir (Lagevrio), were not optimized for efficacy toward SARS-CoV-2 but were repositioned after development for other viruses. Remdesivir and molnupiravir target viral RNA-dependent RNA polymerase (RdRp). Remdesivir is administered by intravenous infusion and molnupiravir, an oral antiviral, has a modest efficacy of about 30% against hospitalization or death in unvaccinated adults with mild or moderate COVID-19 and at least one risk factor for disease progression.^6^ Molnupiravir, a prodrug of a nucleotide mimetic, has raised concerns around potential mutagenicity.^7^

Paxlovid represented a game-changing oral antiviral in acute treatment of COVID-19 and may reduce the risk of “long COVID”, or post-COVID-19 condition (PCC).^8^ It is also strongly recommended by the WHO for individuals at high and moderate risk of hospitalization. This medication is a combination of two drugs: nirmatrelvir and ritonavir. The latter, a human immunodeficiency virus (HIV) protease inhibitor, is a potent inhibitor of P450 CYP3A4, the main enzyme responsible for nirmatrelvir metabolism.^9^ Nirmatrelvir inhibits the SARS-CoV-2 main cysteine protease (Mpro or 3CLpro), while ritonavir is required to boost the exposure of nirmatrelvir to a concentration that provides efficacy against SARS-CoV-2.

Despite its initial success, Paxlovid faces several challenges. First, a recent retrospective NIH study, which included 1,012,910 COVID-19 positive patients at risk of severe illness, revealed that only 9.7% were treated with Paxlovid.^10^ This low rate is primarily due to medical hesitancy regarding potential drug–drug interactions in patients who are often concurrently on other medications for coexisting conditions, in particular elderly patients and those with on long-term medication for cardiovascular disease and diabetes. Second, while Paxlovid has demonstrated a 73% reduction in mortality, this finding underscores the urgent need for additional or combination antivirals to enhance efficacy. Third, Paxlovid is not suitable for certain populations, such as pediatric patients, due to the inhibition of CYP3A4 by ritonavir, which leads to metabolic unpredictability. Finally, the ongoing transmission of the virus, its continuous evolution, and increasing selective pressures may give rise to viral variants that are resistant to 3CLpro inhibitors, including Paxlovid and ensitrelvir. Notably, phylogenetic analyses suggest that many of these resistant variants existed before the introduction of these drugs into human populations and are capable of spreading.^11^ Collectively, these factors highlight the need for ongoing development of antiviral agents.

Beyond the 3CLpro and RdRp, the papain-like protease (PLpro; nsp3) is another promising therapeutic target for developing antiviral agents against SARS-CoV-2. PLpro, like 3CLpro, processes the viral polypeptide. Proteolytic cleavage mediated by 3CLpro occurs at 11 polyprotein sites, whereas PLpro, which recognizes the P4–P1 sequence, LxGG, cleaves at three sites to release nsp1, nsp2, and nsp3. PLpro constitutes residues 1602–1855 of nsp3 (1922aa, 215 kDa).^12,13^ The cysteine protease activity of 3CLpro and PLpro is essential for viral replication, making these enzymes compelling targets for antiviral therapy. Importantly, mutations in 3CLpro and PLpro are significantly less prevalent than in spike protein,^14^ further supporting these enzymes as viable drug targets for emerging variants. Beyond the role of PLpro in viral polyprotein processing, PLpro supports viral replication by perturbation of the host innate immune response. PLpro is a deubiquitinase (DUB) removing ubiquitin (Ub) and ubiquitin-like proteins (UbL), most importantly interferon-stimulated gene product 15 (ISG15), from host proteins.^15−21^ ISG modification of host proteins is a key component of the innate immune response to viral infection and PLpro has evolved to have the greatest DUB efficiency toward ISGylated host proteins.^18,22^

**Figure 1:**
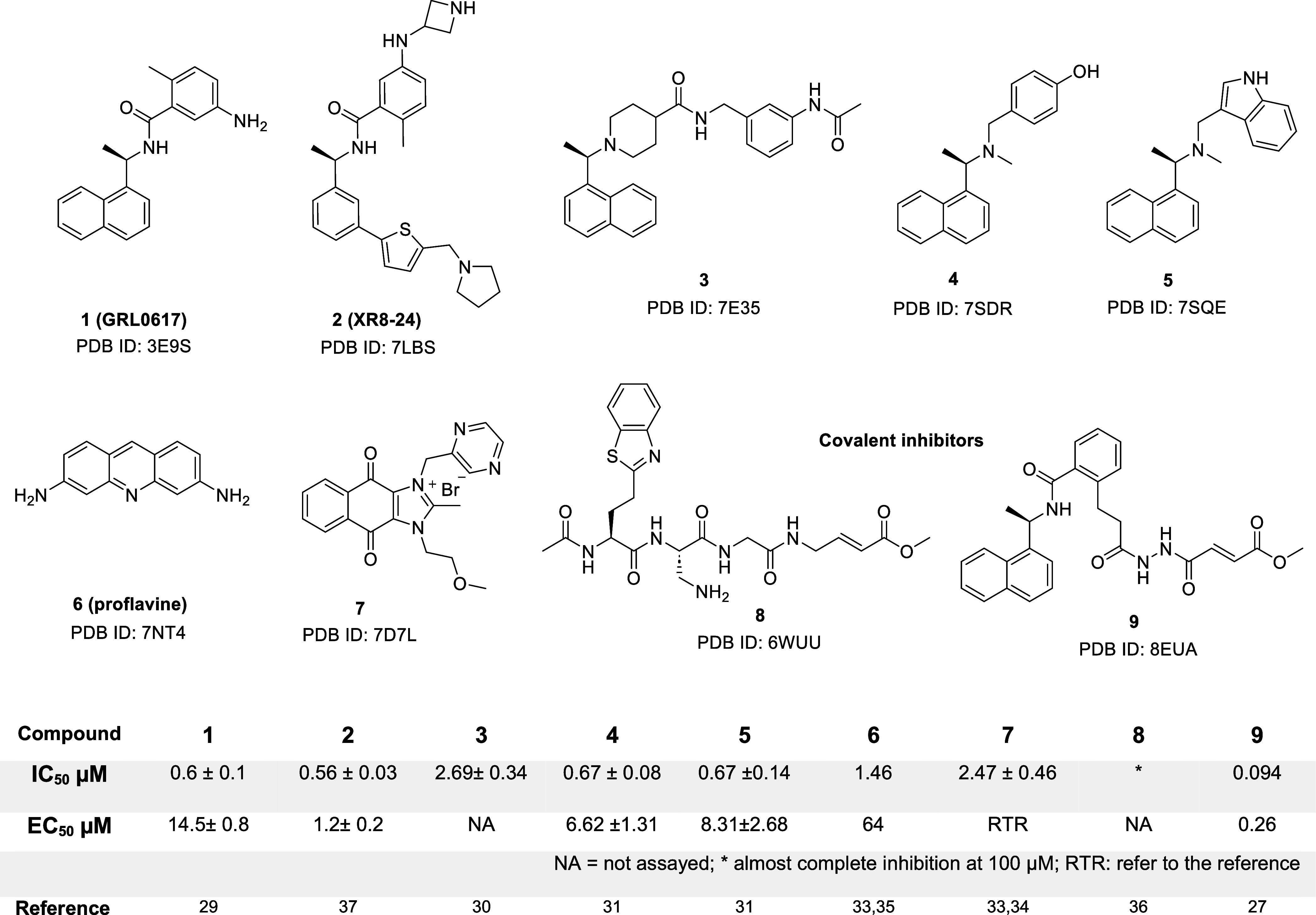
Structures of compounds reported to inhibit PLpro with crystal structure data showing PLpro binding. Reported enzyme inhibition potency (IC_50_) and cell-based antiviral potency (EC_50_). See referenced publications for assay details.

While PLpro is recognized as a promising target, discovery of efficacious and translatable inhibitors has been a challenge. This is primarily due to the characteristics of the P1 and P2 sites (Gly–Gly recognition) that do not provide druggable binding pockets proximal to the active site cysteine. This “classical” inhibition strategy for cysteine proteases places a covalent warhead close to the active site cysteine using P1 and P2 site binding, as seen in Mpro inhibitors. Inhibition of Mpro and human cysteine proteases is readily achieved using this strategy.^23,24^ Consequently, very few submicromolar inhibitors of SARS-CoV-2 PLpro have been reported with experimentally validated antiviral efficacy (Figure 1).^25−28^ The naphthalene derivative GRL0617 (**1**), discovered as an inhibitor of SARS-CoV PLpro by Ratia et al.,^29^ has provided inspiration for structure-based drug design. The weak potency of **1**, itself, along with reported poor metabolic stability, limits development for SARS-CoV-2. Several naphthyl derivatives (**3–5**) have been reported,^30,31^ which inhibit PLpro with submicromolar potency (Figure 1).^32^ The selectivity of other reported inhibitors (**6**, **7**) is problematic.^33−35^ The most promising covalent PLpro inhibitors (**8**, **9**), while achieving impressive potency, suffer from the metabolic lability of the fumarate warhead.^27,36^ Thus, **9**, potent in virus yield reduction assay, fails to demonstrate in vivo efficacy. The field would benefit from in vivo evidence for antiviral efficacy resulting from pharmacological inhibition of PLpro.

**Figure 2:**
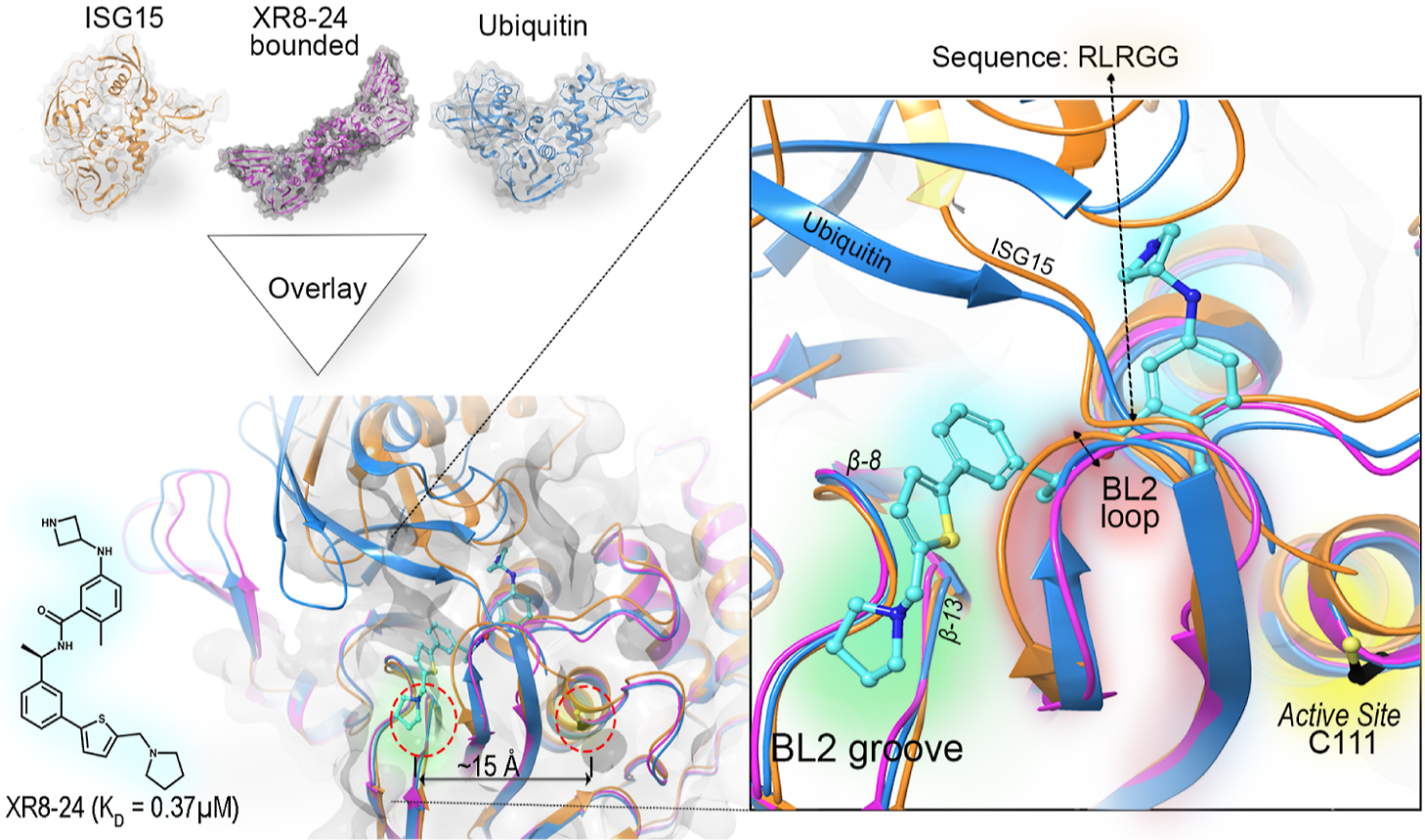
Overlaid structures of PLpro bound with inhibitor XR8–24 (**2**) (purple, PDB: 7LBS), ubiquitin (blue, 6XAA) and ISG15 (orange, 6YVA). PLpro acts as a peptidase and a DUB. Post translational modification by Ub and UbL regulates host protein responses such as antiviral immunity. PLpro recognizes and cleaves the C-terminal RLRGG sequence of many UbLs acting as a DUB toward host proteins. PLpro DUB activity is hypothesized to cause dysregulation of host immune response.

To address the featureless active site and to explore structural space beyond **1**, we searched for and discovered a novel binding site, the “BL2-groove”, distal from the active site (15 Å). Through structure-guided medicinal chemistry, supported by new X-ray cocrystal structures, novel, noncovalent PLpro inhibitors were designed that engaged the BL2-groove, positioned between β8 and β9 strands, and adjacent to the BL2-loop (Figures 1 and 2).^37^ The BL2-loop itself is recommended as a site for ligand design, because the mutation rate of residues in the vicinity of the active site and BL2-loop is low. Of 2.6 million PLpro sequences in the NCBI Virus database, 21% had mutations in the PLpro domain:^38^ the most prevalent were A145D, P77, and P223.^39^ The BL2-groove is now studied as an important feature of PLpro for drug design.^40−42^

Herein we identified novel inhibitors that bind to the BL2-loop to block substrate access to the active site (PDB: 7CJDFigures 1–3A). The most potent BL2-loop inhibitor, **11** (IC_50_ = 110 nM; PDB 7LBR), bound to PLpro with the highest affinity (*K*_D_ = 113 nM) and lowest off-rate of all inhibitors studied. Given the flexibility of the BL2-loop, it is likely that binding is by an induced fit mechanism, in which binding of these BL2-loop inhibitors closes the BL2-loop (Figure 2). Our novel, inhibitors, **2** and **10**, although weaker PLpro ligands than **11**, displayed low micromolar potency against viral infection in both Vero E6 and A549-hACE2 cells. To further explore the structure–activity relationship (SAR) of our noncovalent inhibitors and select a compound for testing in a mouse model, we synthesized further examples, including prodrugs and a PLpro/RdRp mutual prodrug. Ultimately, compound **10** was selected for exploration of bioavailability and tested in the MA10 mouse model of SARS-CoV-2 infection.^37^

**Figure 3:**
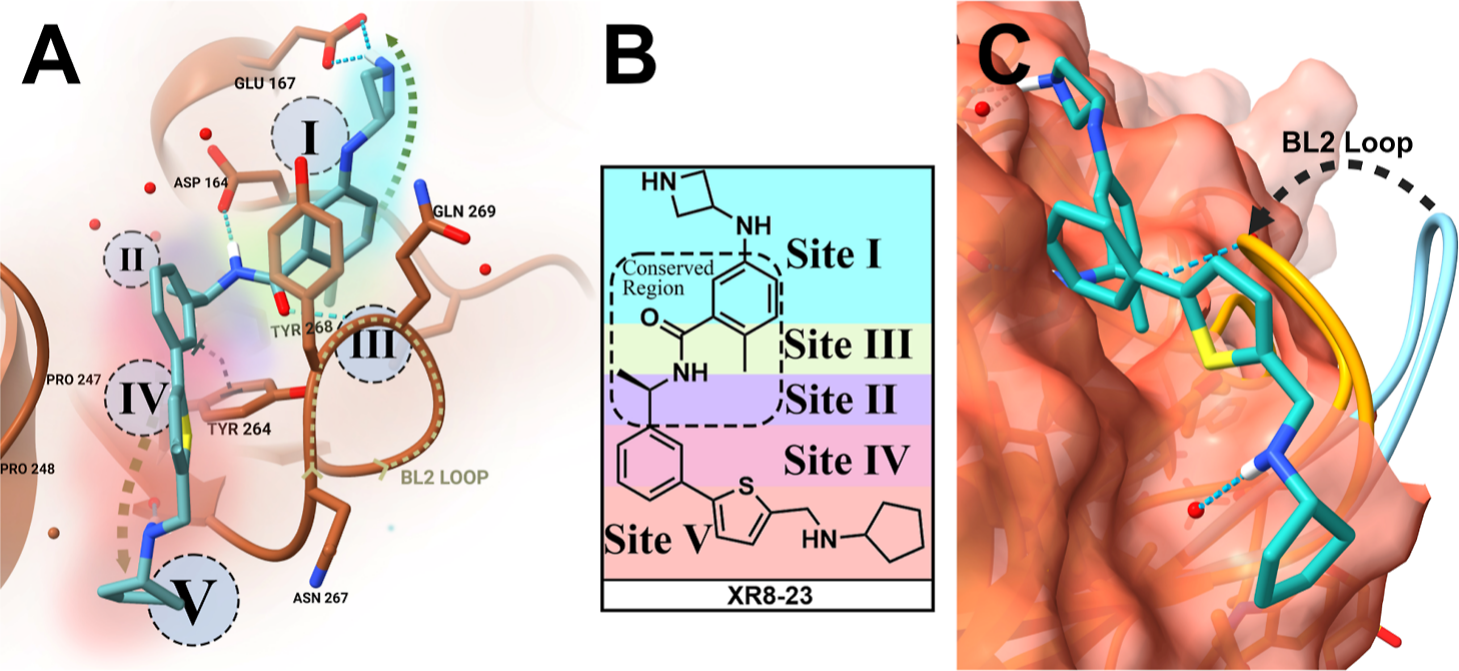
Structure-guided design of SARS-CoV-2 PLpro inhibitors. (A) Relevant sites (*Sites I*–*V*) and important contact amino acids considered in drug design of BL2-loop inhibitors. Inhibitor **10** (turquoise) is displayed docked to a PLpro cocrystal structure with XR8–24 bound (PDB ID: 7LBS). (B) SAR strategy for further design and development of BL2-loop inhibitors based on **10** 2D: *Site I* cyan, *Site II* as purple, *Site III* pale green, *Site IV* pink, *Site V* coral. (C) Overlaid apoenzyme BL2-loop (7CJD cyan) with **10** (turquoise) bound in the BL2-loop and BL2-groove, showing closing of the BL2 on inhibitor binding.

## Design & Optimization

We published the design strategy leading to **2**, **10**, and **11**.^37^ This optimization was driven by potency (IC_50_) for PLpro inhibition, measured using a short peptide substrate Z-RLRGG-AMC, and affinity, as measured by surface plasmon resonance. We identified four potential regions that could be targeted (*Sites I*–*V*) (Figure 3A). These putative inhibitor binding sites are defined by binding interactions between PLpro and either Ub- or ISG15-adducted substrates as described in crystal structures (e.g., Ub: PLpro SARS-CoV PDB 4MM3; Figure 2). Despite achieving up to a 24-fold improvement in potency and a significant enhancement in metabolic stability over compound **1**, the optimization in *Site I*, *Site IV* and *Site V* remained inadequate. Consequently, we designed and synthesized more than 50 additional analogs to further explore the SAR with the expectation that modifications to improve bioavailability may lead to some sacrifice of potency and affinity.

Glu167 forms electrostatic interactions with the Arg72 of Ub-substrates in *Site I*, and Glu167 binding may be leveraged using electrostatic interactions with cationic amine substituted ligands. However, a potency increase of only up to 2-fold was observed for cationic amines that form electrostatic interactions with Glu167. This increase is significantly lower than that of a typical electrostatic interaction (≈2 kcal/mol), equivalent to a 14-fold increase in affinity. This modest improvement suggests the presence of a substantial desolvation penalty around *Site I*. Therefore, designing new analogs capable of rearranging the water network could potentially mitigate this desolvation penalty, thereby enhancing both potency and drug-like properties.

*Site II* includes the charged side chains of Arg166 and Asp164 that form an electrostatic interaction (Figure 3A). In the Ub: PLpro complex (PDB 4MM3), Arg166 and Asp164 of PLpro are H-bonded to Gln49 and Arg72 of ubiquitin, respectively. *Site III* incorporates the P3 substrate-binding site that is formed by helix 5, the BL2-loop, and adjacent hydrophobic residues (Tyr264, Tyr273, and Leu162). *Site IV* is occupied by naphthalene of **1** in PLpro cocrystal structures. SAR studies leading to inhibitors **2**, **10**, and **11**, demonstrated that *Site II* and *Site III* would not accommodate significant modifications nor replacement of the *N*-isopropyl-2-methylbenzamide moiety. The orientation of the benzamide and naphthalene rings of **1** is essential for BL2-loop binding. Only biaryl group replacements were successful in improving binding affinity and inhibitor potency, as fully rationalized by the cocrystal structures obtained.

In addition to exploring known binding interactions in *Sites I–IV*, we identified a novel binding site, the BL2-groove (*Site V*). The BL2-groove, at the base of the BL2-loop, is not a known binding site for Ub- or ISG15-modified substrates; however, the presence of several hydrophobic (e.g., Pro248 and Pro299) and hydrogen-bonding residues (e.g., the Gly266 backbone amide) drove us to explore *Site V* (Figure 3A,B). Several noncovalent PLpro inhibitors were identified with potency and affinity better than 500 nM, notably **2**, **10**, and **11**, and were shown to engage the BL2-groove in cocrystal structures.^37^ The pyrrolidine group of **2** is oriented perpendicular to the thiophene and sits in the BL2-groove at *Site V*, engaging in van der Waals interactions with Pro248, Tyr264, Tyr268, and forming a water-mediated hydrogen-bond with the carbonyl oxygen of Gly266 (Figure 3A). Collectively, these potential binding interactions were validated in our cocrystal structures. This motivated us to further explore the SAR in these regions.

### *Site IV*/*V* SAR

The family of aminoazetidine-substituted phenylthiophenes, for which cocrystal structures were obtained, includes **11**, which is 15-fold superior to **1** (GRL0617) in terms of enzyme inhibition potency (Table 1). The enantiomeric analogue **14** is 2-fold less potent showing that the region extending from the BL2-groove can mediate small differences in potency. Methylation of the thiophene ring in the analogs of parent compounds **2**, **10** and **11** led to the compound trios **15–17** and **18–20**. Potency across the two trios followed the same relative trend, with 4-methyl substitution leading to significantly lower potency, presumably owing to the perturbation of the optimal torsional angle between the benzene and thiophene rings, which we previously reported as important for binding to the BL2-groove.^37^

**Table 1:**
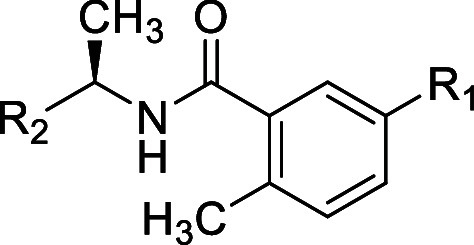

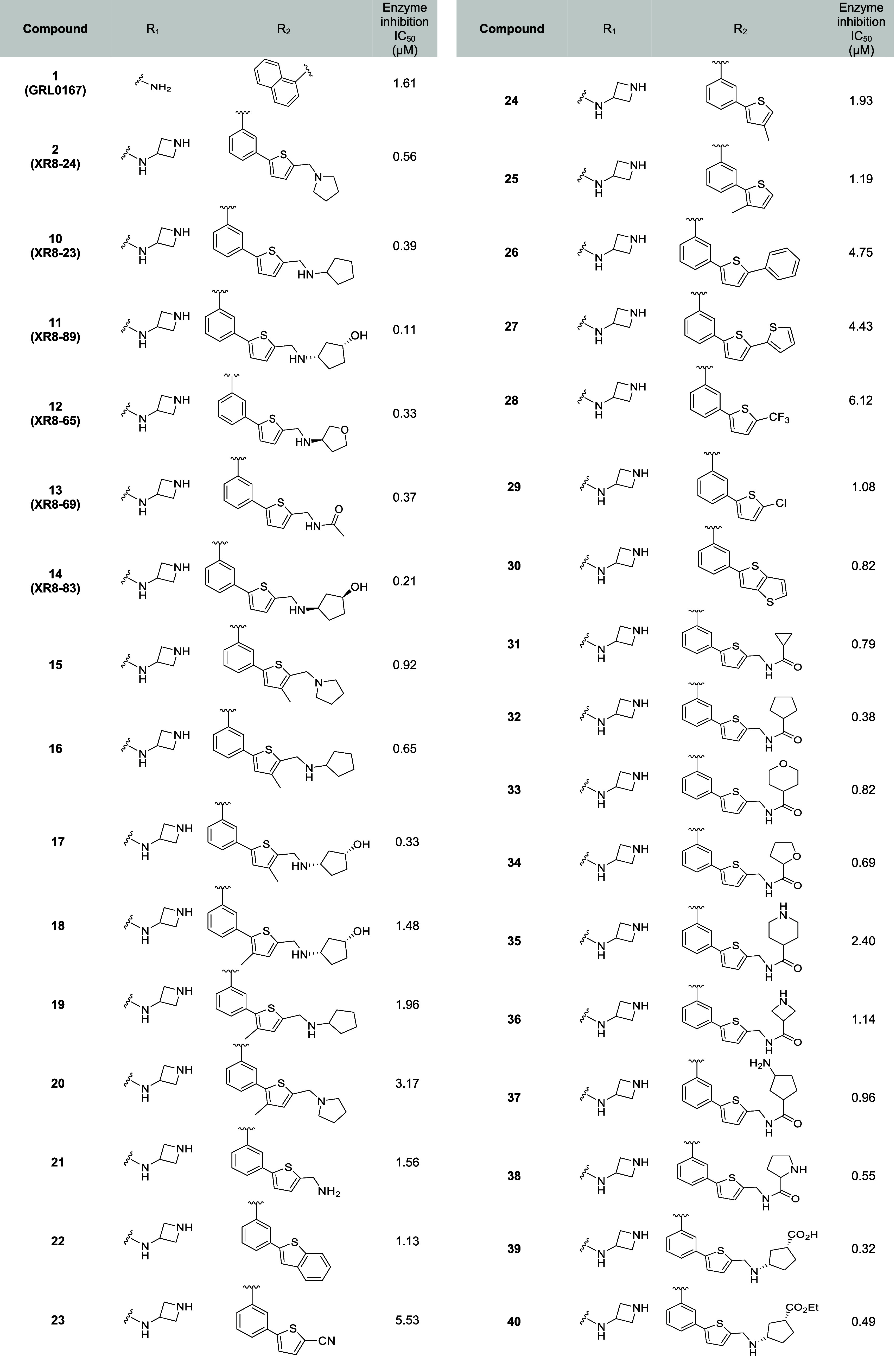
Structures and Potency for PLpro Enzyme Inhibition

Replacement and/or truncation of the thiophene substituents present in parent compounds **2**, **10** and **11** led to loss of inhibitory potency in all examples and in the cases of analogues **23**, and **26–28**, the potency worsened to 4–6 μM. These weaker inhibitors are characterized by a rigid hydrophobic extension from the 2-position of the thiophene ring. Bicyclic analogs, such as **22** and **30**, were better tolerated. Of the original PLpro inhibitors, compound **13** incorporated an amide in place of an amine substituent extending from the thiophene and retained a potency of 370 nM. Extensive further exploration of thiophene-2-alkylamides was made incorporating various alicyclic substituents (**31–38**) maintaining potency similar to inhibitor **13**. Docking suggests that substituents at the 2-thiophene position extend toward solvent; therefore, compounds that contain a flexible substituent with H-bonding groups (**31–38**) were more potent PLpro inhibitors than **26–28**. Compound **32** showed similar potency to **13** and incorporation of a piperidine ring (**35**) gave a significant reduction in potency.

Mindful of the potential need to prepare prodrugs to increase bioavailability and the potential for synthesizing conjugates, such as hybrid drugs, incorporation of a carboxylic acid was explored (**39**). This resulted in an inhibitor with equivalent potency to **10** that allowed esterification with a modest loss of potency (**40**).

### *Site I* SAR

To explore *Site I* interactions, particularly in rearrangement of the water network, modifications were made to the R_1_ group while incorporating several of the modifications made to R_2_ introduced in Table 1. Replacement of the aminoazetidine (Table 1) with oxyazetidine, in general, led to loss of potency for enzyme inhibition of approximately 3-fold (Table 2): for example, comparing oxyazetidine **43** to aminoazetidine **11**. Comparison of R_1_ substituents aminoazetidine (**10**), oxyazetidine (**41**), oxypiperidine (**44**), and oxyethylamine (**47**) (IC_50_ = 0.39, 0.96, 0.75, 0.26 respectively) showed some sensitivity to the substituent occupying *Site 1.* This was also shown in comparison of aminoazetidine (**2**), oxyazetidine (**42**), oxyethylamine (**48**), and oxypyrrolidine (**49**) (IC_50_ = 0.56, 0.81, 0.57, 1.2, respectively). Simple amino substituents at R_1_ led to reduced potency compared with aminoazetidines (**53–55**) (0.6 < IC_50_ < 1.7). However, all cationic R_1_ substituents were superior to neutral amide substitutions (**50–52**) (1.5 < IC_50_ < 2.4), which are incapable of optimal interactions with Glu-167 at *Site I*. Although several modifications of R_1_ were explored, there remains scope for further exploration of *Site I* to gain affinity.

**Table 2:**
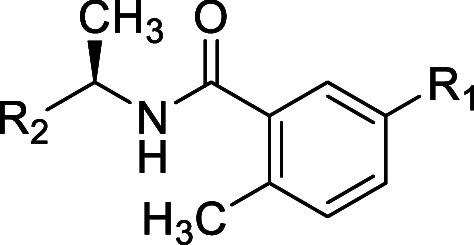

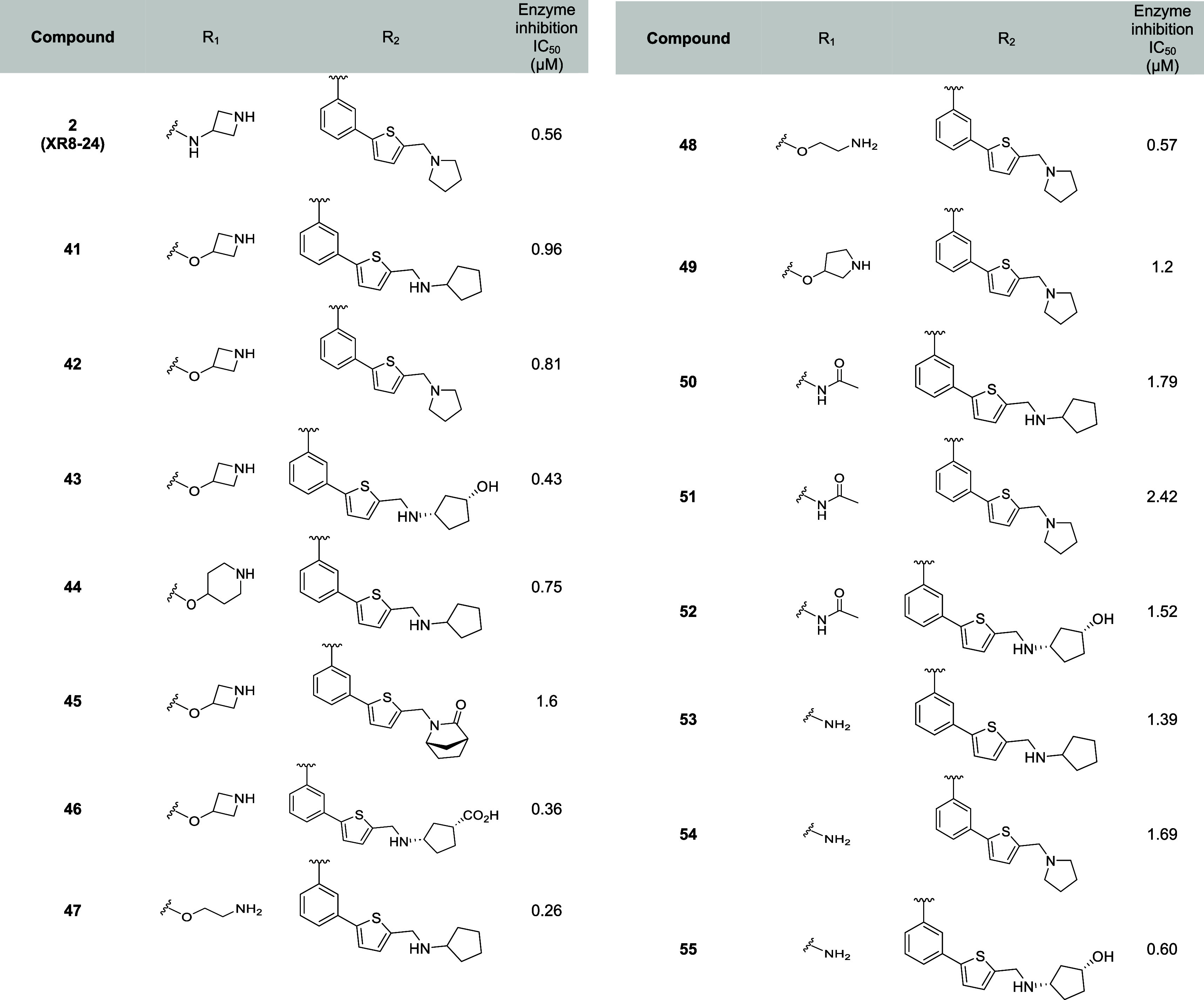
Structures and Potency for PLpro Enzyme Inhibition

Replacement of the R_2_ thiophene group was explored with both oxyazetidine and aminoazetidine substituents at R_1_ (Table 3). Furan (**56**) and thiazole (**57**, **58**) replacements led to 2–3 fold reduced potency compared with corresponding compounds (**41** and **10**), and the pyrrole analog tested (**59**) was one of the weakest inhibitors studied. Further heterocyclic substitutions were not explored.

**Table 3:**
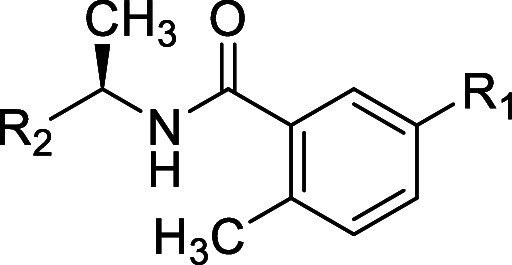

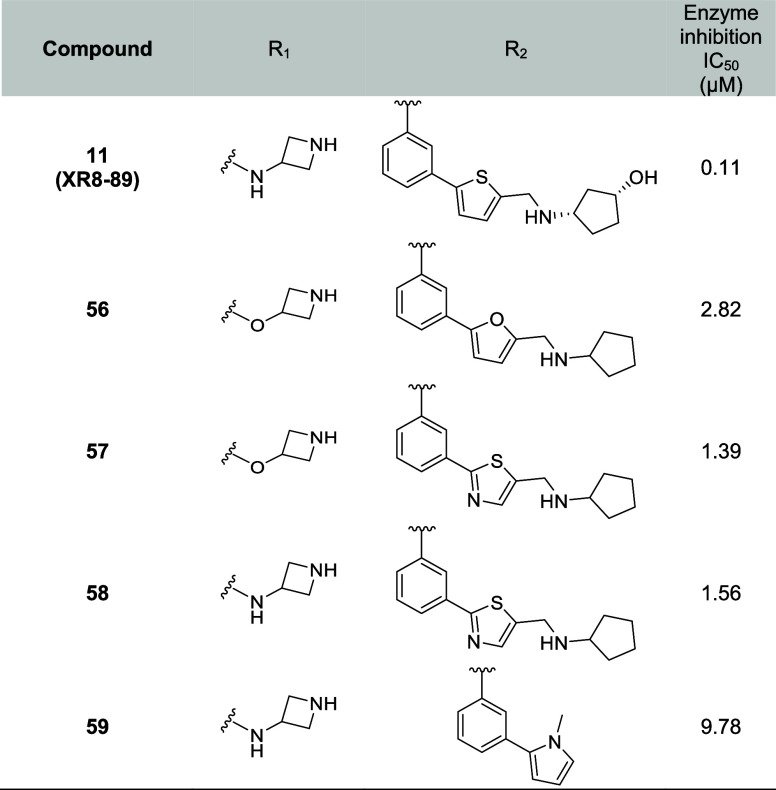
Structures and Potency for PLpro Enzyme Inhibition

### Prodrug Exploration

Several analogs containing hydrophilic substituents, extending from the BL2-groove, *Site V*, did not exhibit significant loss of enzyme inhibitory potency. The cyclopentanol derivative (**11**) was the most potent in enzyme assays of the first generation BL2-groove inhibitors, and replacement of the secondary alcohol with a carboxylic acid group in the aminoazetidine (**39**) and oxyazetidine series (**46**) maintained IC_50_ < 500 nM (Table 4). These observations are compatible with the potential for prodrug synthesis by extending PLpro inhibitor structures into the water-exposed region beyond *Site V*.

**Table 4:**
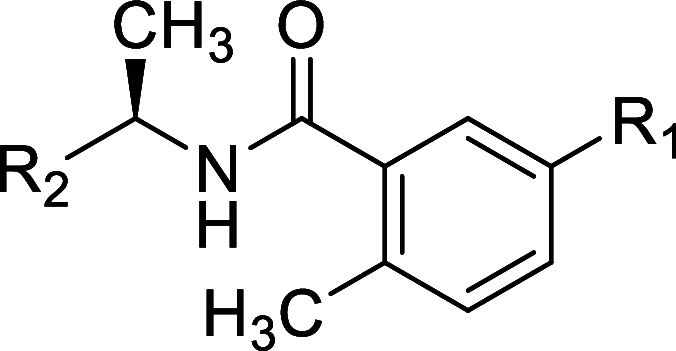

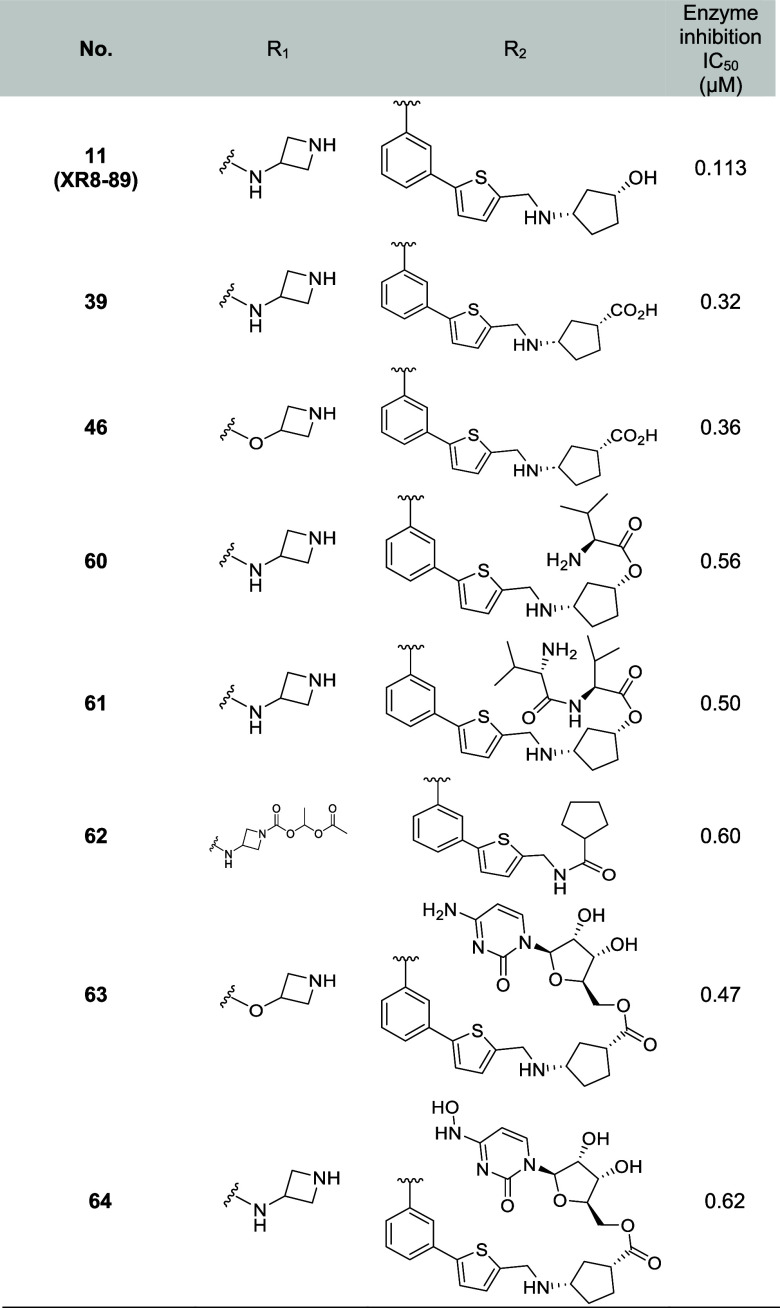
Structures and Potency for PLpro Enzyme Inhibition

Several FDA-approved, ester-linked, antiviral agents incorporate valine to improve the physicochemical and oral bioavailability characteristics; for example, valganciclovir.^43,44^ Such prodrugs use the solute carrier 15 transporters (SLC15s) that transport dipeptide and tripeptides.^45^ The valine (**60**) and Val–Val (**61**) modified ester prodrugs of **11** were synthesized and shown to maintain IC_50_ < 600 nM (Table 4). An alternative prodrug strategy that is more commonly used for ester-linked prodrugs utilizes activated acyloxyalkyl esters, including axetils. To prototype this approach for a carbamate prodrug (**62**), we used compound **32** that is a potent PLpro inhibitor with good bioavailability administered i.p. but without oral bioavailability (Figure S1). We did not interrogate enzyme inhibition assays for prodrug activation at this point, since we could not obtain a stable formulation for p.o. or i.p. administration using these prodrug approaches.

Given the ability to modify BL2-groove PLpro inhibitors beyond *Site V*, it was logical to further explore prodrugs using this ligation position. Two mutual prodrug strategies were tested, targeting the inhibition of two SARS-CoV-2 enzymes, PLpro and RdRp. Molnupiravir (**65**; EIDD-2801), used clinically to treat COVID-19, is a prodrug of the RdRp inhibitor (**66**; EIDD–OH). Despite the bulk of the molnupiravir pharmacophore, the mutual prodrug **64** and the related oxyazetidine (**63**), retained submicromolar potency for PLpro inhibition (Table 4). Mice were administered **64** (25 mg/kg i.p.) followed by measurement of the released bioactivation products, PLpro inhibitor (**39**) and the RdRp inhibitor (**66**), in plasma using LC–MS/MS. The mutual prodrug was undetectable in plasma between 0.5 to 5 h after administration; however, the bioactivation products, **39** and **66**, were both detected with *C*_max_ of 7910 and 4170 ng/mL, respectively, which represent approximately equimolar concentrations. The pharmacokinetics for exposure were identical with *t*_1/2_ = 2 h for both metabolites (Figure S2). Taken together, the PK data suggest the undesired breakdown of the prodrug in the i.p. cavity. The 1,3 relationship of the cyclopropyl amino and carboxyl groups of **56** sensitizes the ester to hydrolysis via anchimeric assistance, since this enables a 1,5 and 1,6 relationship with the carbonyl *C* and *O* of the ester group, respectively. The same ester reactivity issue in prodrugs **60** and **61**, which was observed for mutual prodrug **64**, likely led to the lack of stability of prodrug formulations.

## Antiviral Activity in Cell Cultures

The antiviral activity of selected inhibitors was tested using the plaque assay in Vero cells, infecting with the original Washington strain of SARS-CoV-2 followed by transfer of virus-laden supernatants to a second Vero cell culture for viral plaque counting. Vero cells are widely employed in antiviral assays and virology research for SARS-CoV-2 and viruses from other families, despite Vero cells having very high efflux capacity and high expression of the P-gp efflux transporter. Consequently, antiviral assays in Vero cells are commonly performed in the presence of the efflux pump inhibitor CP-100356 that itself may confer cytotoxicity. For example, the effect of CP-100356 in one study on SARS–CoV-2 infection of Vero cells was ca. 100-fold left-shift in the response to nirmatrelvir.^46^

**Figure 4:**
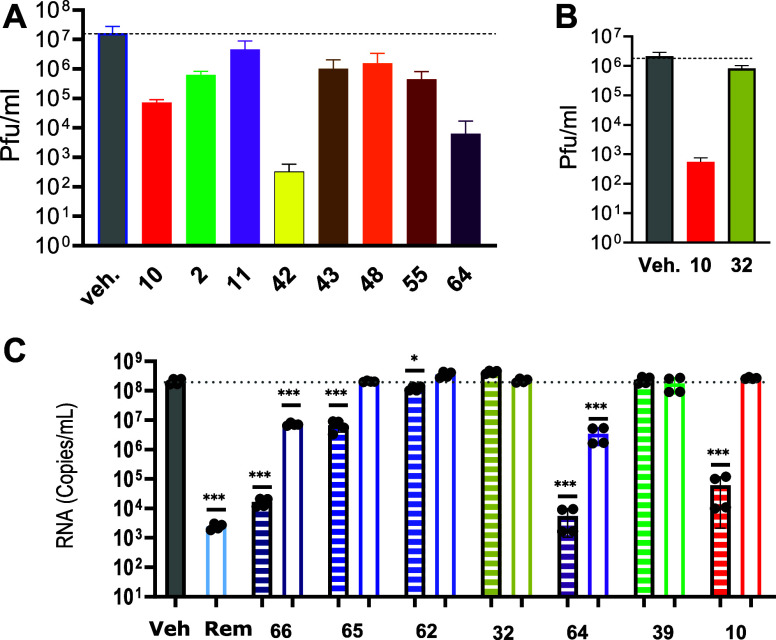
Activity of SARS-CoV-2 PLpro inhibitors in Vero cells. Cells were infected with SARS-CoV-2 (strain WA1, MOI = 0.01) in the presence of 2 μM (open bars), 10 μM (filled bars) 20 μM (striped bars) of the indicated compounds and 2 μM of the P-gp inhibitor CP-100356. Vehicle-treated cells served as controls. The amounts of infectious virions in the cell supernatants were determined 2 days later by plaque assay (A,B) or by RT-PCR of viral RNA (C): unpaired *t*-test vs vehicle *p < 0.05 ***p < 0.001.

As reported previously, the relative potency for enzyme inhibition of the first-generation inhibitors did not translate to efficacy in inhibiting viral replication in Vero cells, with **11**, a more potent PLpro inhibitor than **10** and **2**, being the least efficacious compound at 10 μM concentration in cells (Figure 4A). The oxyazetidine analogue (**42**) of the first-generation inhibitor **2**, is a weaker PLpro enzyme inhibitor but showed relatively higher efficacy in Vero cells. Conversely, the oxyethylamine congener (**48**) showed low efficacy. Furthermore, direct comparison of the amine **10** with its amide congener (**32**) demonstrated almost complete loss of antiviral activity (Figure 4B). Measurement of viral RNA by RT-PCR provides an alternative approach to quantifying antiviral activity (Figure 4C). The lack of activity of the amide (**32**) was recapitulated in this assay and was not greatly improved by its prodrug (**62**), discouraging further work on the prodrug.

The most potent PLpro inhibitors in cell-free assays (e.g., **39**) possess substituents extending from the BL2-groove into a solvent exposed region. The collected data shows that for inhibitors, bearing substituents extending from the BL2-groove, potency in the enzyme assay does not translate to cell-based antiviral assays. The implication is that the solvent exposed region adjacent to the BL2-groove is occluded in the cellular environment, leading to loss of activity in cells. Based on the antiviral data and the extended SAR presented herein, we select compound **10** and **42** for further pharmacokinetic studies.

## Bioavailability & Preclinical Efficacy in Mouse Models

Compound **42**, which showed impressive antiviral activity, was compared with **10** for in vivo exposure; however, **42** demonstrated significantly lower exposure compared to **10** (Figure S3) We have previously reported the stability of inhibitors **10** and **2** in liver microsomes and the plasma exposure as approximately 12–13 μM when delivered i.p. (at 50 mg/kg i.p. in male C57BL/6 mice, **10** and **2** gave *C*_max_ values of 6130 and 6403 ng/mL, respectively).^37^ Importantly, at this dose and route of administration, compound **2** displayed overt toxicity. Consequently, compound **2** was deselected and our focus shifted to further exploring inhibitor **10** using alternative routes of administration.

Human plasma stability for **10** was high, as expected, with estimated t_1/2_ = 60 h (compared to reference propantheline t_1/2_ = 24 min). Plasma protein binding (96.7%; compared to ketoconazole control 99.3%) was acceptable. A tolerability study was performed in male C57BL/6 mice administering **10** i.v (5–10 mg/kg) or s.c. (100 mg/kg), demonstrating 10 mg/kg qd and 5 mg/kg bid to be the maximum tolerable doses via i.v. administration. The PK of **10** was compared with drug delivered i.v. (2 mg/kg) or s.c. (50 mg/kg) to male C57BL/6 mice and i.v. (2 mg/kg) and s.c. (20 mg/kg) to male hamsters (Table 5; Figure S4). In accord with the previous observations on i.p. administration in mice, the bioavailability of **10** was good with minor species differences.

**Table 5:** PK Parameters for **10**

	plasma *C*_0_ or *C*_max_ ng/mL	plasma *C*@ 4 h ng/mL	plasma AUC_inf_ h × ng/mL	plasma clobs mL/min/kg	lung/plasma conc ratio
i.v. mouse^a^	734 ± 125	5.38 ± 0.28	305 ± 46	111 ± 16	
s.c. mouse^b^	3193 ± 667	1257 ± 153	15,067 ± 1298		
i.v. hamster^c^	2916 ± 474	4.0 ± 0.92	448 ± 32	75 ± 5	
s.c. hamster^d^	1217 ± 276	933 ± 168T	10,900 ± 564		
i.v. mouse^e^					5.4
s.c. mouse^f^					12.9

a 2 mg/kg.

b 50 mg/kg.

c 2 mg/kg.

d 20 mg/kg.

e 5 mg/kg @ 5 min.

f 100 mg/kg @ 30 min.

Since the lungs are a major organ for drug exposure in SARS-CoV-2 treatment, we extended the PK assessment to include measurement of drug in lung tissues yielding the lung/plasma exposure ratio. Measured 5 min after i.v. drug administration, the lung/plasma ratio for **10** was 3.3–5.3 (Tables S1–S4). After s.c. administration (100 mg/kg), the lung/plasma ratio measured at 30 min was 12.8, with the absolute drug concentration in lung tissues being 90 ± 1.8 μg/mL (Tables S5 and S6). The serendipitous accumulation of drug in lung tissues at tolerable doses is seen as positive and supporting in vivo testing in a mouse model.

**Figure 5:**
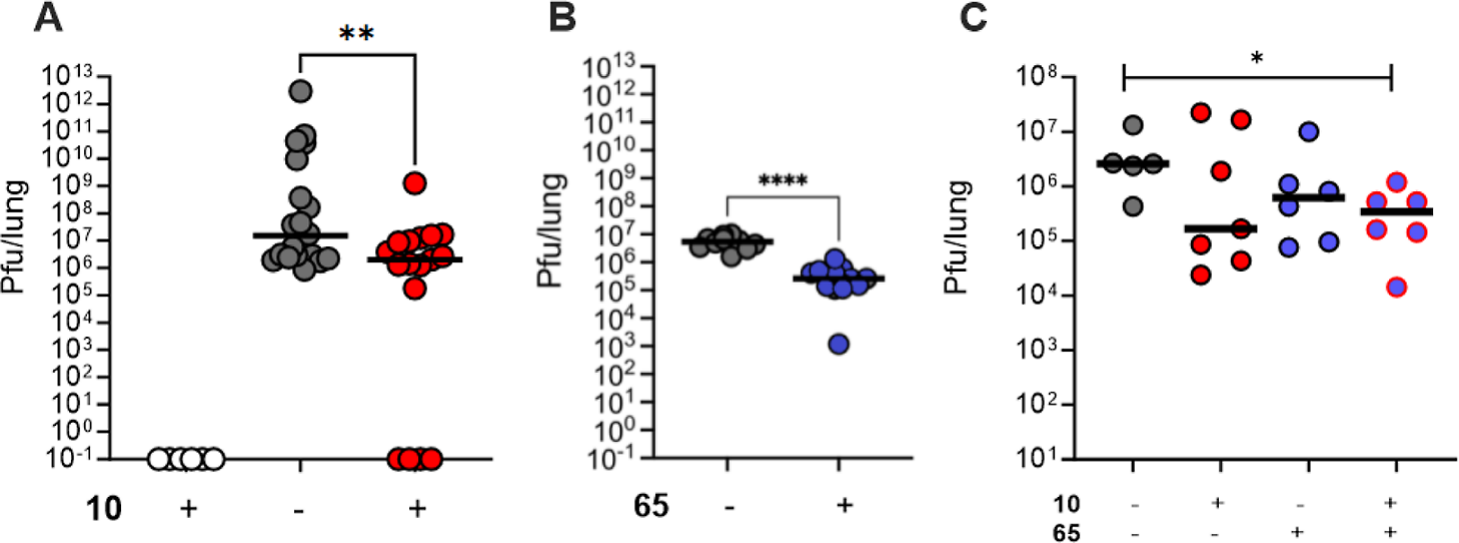
(A) Viral titers of infectious SARS-CoV-2 MA10 virions in the lungs of mice treated with **10** (10 mg/kg i.p. bid) or vehicle, compared to a sham control arm treated with **10** (10 mg/kg i.p. bid). Mice were sacrificed 24 h after intranasal infection. Viral titers of infectious virions in the lungs were measured after 2 days by serial dilutions of lung homogenates in viral plaque assays. Each dot represents one mouse. (B) Viral titers of infectious SARS-CoV-2 MA10 virions in the lungs of mice treated with **65** (Molnupiravir, 150 mg/kg p.o. bid) or vehicle. Mice were sacrificed 24 h after infection. (C) Viral titers of infectious SARS-CoV-2 MA10 virions in the lungs of mice treated with **10** (10 mg/kg i.p.), **65** (molnupiravir, 150 mg/kg p.o.), vehicle, or a combination of **10** (10 mg/kg i.p.) and **65** (molnupiravir, 150 mg/kg p.o.). Drug administration was immediately before infection and then at 6, 10, and 22 h prior to sacrifice at 24 h (each dot = 1 mouse). Vehicle-treated or mock-infected animals served as controls. By Mann–Whitney test: *p ≤ 0.05; **p ≤ 0.005; ****p ≤ 0.001.

In the first infection protocol, C57BL/6 mice were infected with 5 × 10^4^ Pfu of mouse-adapted SARS-CoV-2 MA10, intranasally under anesthesia, 2 h after drug or vehicle treatment. A sham control arm was treated with **10**. Viral titers of infectious virions in the lungs were measured by serial dilutions of lung homogenates in viral plaque assays following sacrifice of mice 48 h after infection. Drug treatment was **10** (10 mg/kg i.p.) or **65** (Molnupiravir, 150 mg/kg p.o.; according to the literature) twice daily for a total of four treatments. Both **10** and **65** (molnupiravir, as positive control) reduced viral loads in lung tissues significantly (Figure 5A,B). In the vehicle control group 2 days after infection, several mice had extremely high viral loads; therefore, we switched to a shorter viral exposure, measuring lung virions at an earlier time point, 24 h after infection. The same number of drug treatments were administered using this protocol and a combination arm was included treating with **10** and **65** (molnupiravir). After 24 h exposure to SARS-CoV-2 MA10, a significant reduction in viral load was observed in the combination arm (Figure 5C). This is the first in vivo proof-of-concept of the efficacy of a selective PLpro inhibitor that has submicromolar potency for enzyme inhibition. This antiviral efficacy was benchmarked against treatment with the RdRp inhibitor prodrug molnupiravir (**65**) at a dose previously reported as efficacious. The significant efficacy of the combination of an RdRp-targeted agent with a PLpro-targeted inhibitor supports this combination as a promising strategy for pharmacotherapy of SARS-CoV-2.

## Antiviral Activity Against SARS-CoV-2 Variants

**Figure 6:**
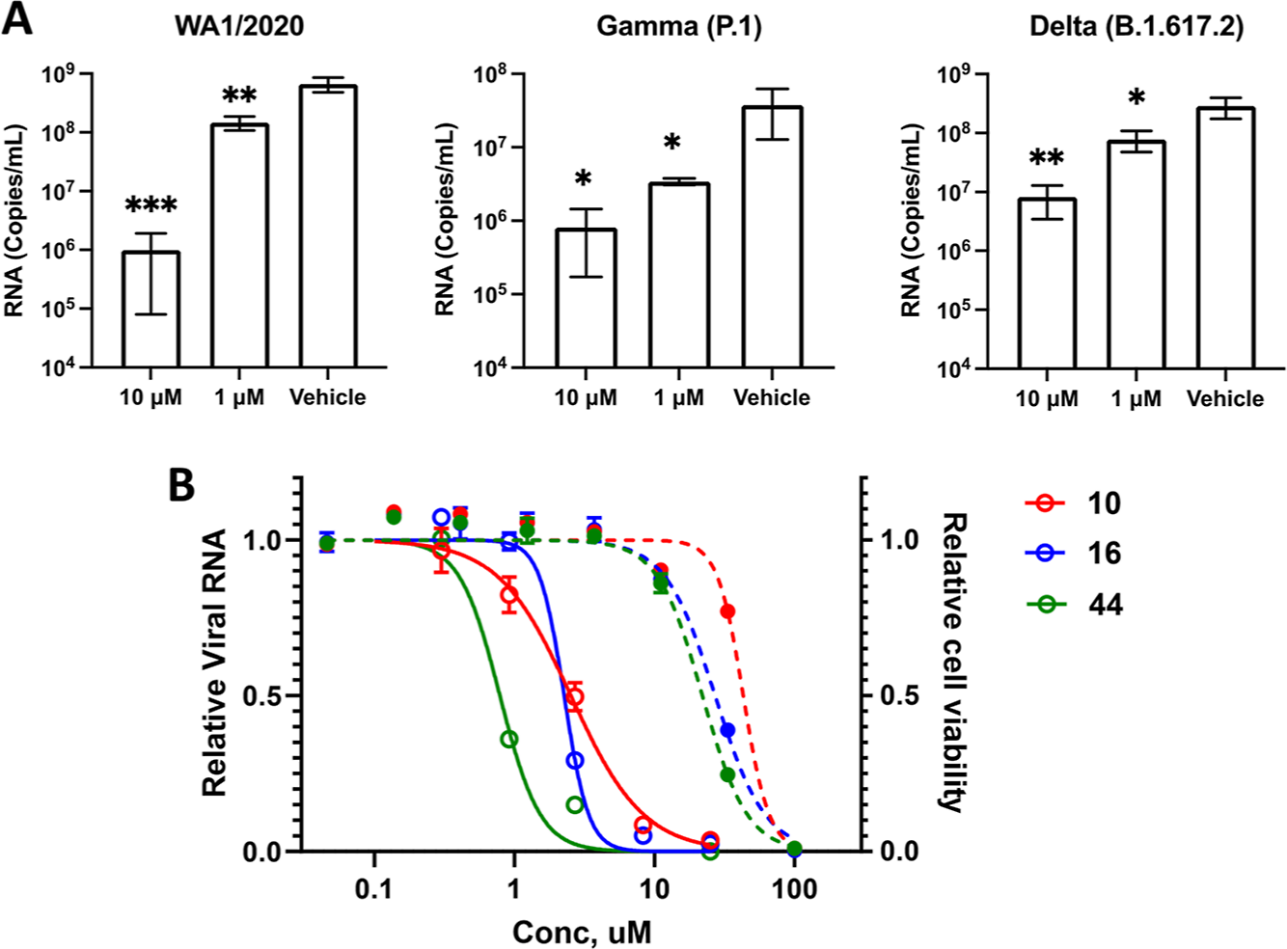
Potent antiviral efficacy in SARS-CoV-2 VOC. (A) To measure the reduction in virus yield, A549-hACE2 cells were infected with MOI = 0.01 of SARS-CoV-2 variants cultured in Vero E6 cells with and without various concentrations of **10**. After 48 h, supernatants were harvested, and RNA was isolated and quantified by reverse-transcription quantitative PCR (RT-qPCR). The data show mean ± SD from replicate measurements. The statistical significance of differences was calculated using Student’s *t*-test. ***p ≤ 0.001; **p ≤ 0.01; *p ≤ 0.05; compared to vehicle group. (B) To measure concentration–response and potency in reducing virus yield in an omicron VOC (BA.1), compound **10** was compared with PLpro inhibitors **16** and **44** in A549-hACE2 cells. Cells were infected with MOI = 0.03. After 48 h, cell lysate was collected and RNA was isolated and quantified by RT-qPCR. In parallel, cell cultures were treated with test compounds for 48 h and cell viability measure by CellTiter-Glo assay. The data show replicates for viral RNA and cell viability.

We next explored the efficacy of compound **10** against SARS-CoV-2 variants. Vero cells present a challenge for antiviral drug discovery, because of their highly efficient drug efflux, requiring coadministration of an efflux pump inhibitor. Conversely, the human lung epithelial cell line, stably expressing the SARS-CoV-2 accessible human ACE2 receptor, A549-hACE2 cells, does not impose such efficient drug efflux. Consequently, inhibitor **10** was tested in A549-hACE2 cells infected with the original Washington strain of SARS-CoV-2 and two VOC, Gamma (P.1) and Delta (B.1.617.2) (Figure 6A). The human lung epithelial cell line, stably expressing the SARS-CoV-2 accessible human ACE2 receptor, A549-hACE2 cells, does not impose the need for CP-100356 cotreatment. Consequently, inhibitor **10** was tested in A549-hACE2 cells infected with the original Washington strain of SARS-CoV-2 and two VOC, Gamma (P.1) and Delta (B.1.617.2) (Figure 6A). The noncovalent, BL2-loop inhibitor **10** did not lose activity against either VOC, suggesting the potential of PLpro as a broad-spectrum anti-COVID target.

Omicron VOC have largely replaced other strains in postpandemic circulation; therefore, **10** was tested in the Omicron BA.1 VOC of SARS-CoV-2 (Figure 6B). Compounds obtained in the SAR exploration described herein, were screened for antiviral activity in the BA.1 strain and full concentration–response was measured for **16**, the 2-methylated congener of compound **10**, and **44**, the oxypiperidine analogue of **10**. The enzyme inhibition potency of these two analogues was approximately 700 nM. All three analogues showed antiviral activity toward BA.1 in A549-hACE2 cells: **10** (EC_50_ = 2.5 ± 0.15 μM); **16** (EC_50_ = 2.3 ± 0.22 μM); **44** (EC_50_ = 787 ± 68 nM). Cell viability of **10** (CC_50_ = 43 ± 1.8 μM); **16** (CC_50_ = 27 ± 1.4 μM); **44** (CC_50_ = 22 ± 0.9 μM) was measured to assess the selectivity index: **10** (SI = 17); **16** (SI = 12); **44** (SI = 28).

## Chemistry

**Scheme 1:**
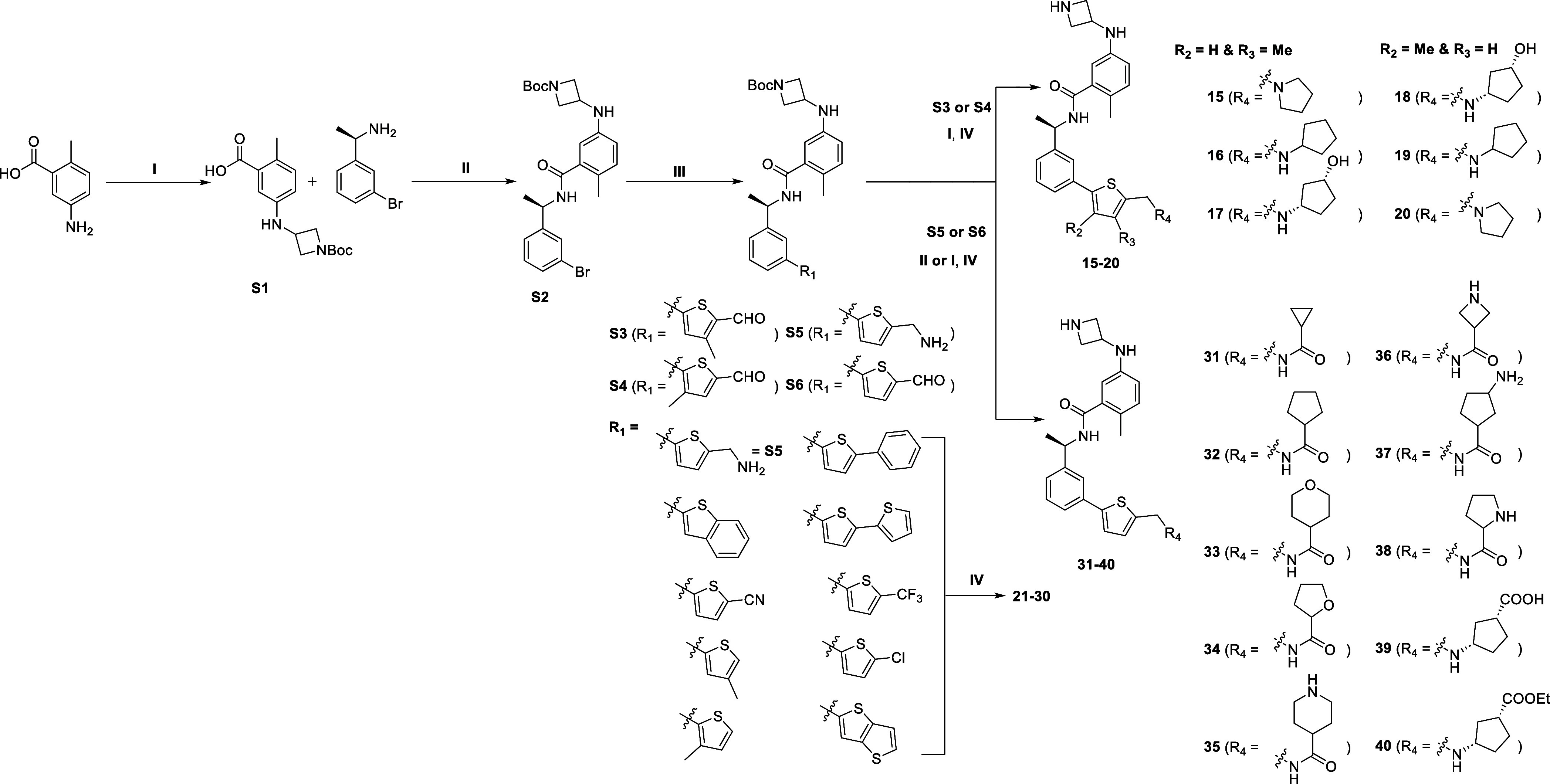
Synthesis of **15–40** Reagents and conditions: (I) amines, aldehydes, or ketones, HOAc, NaBH_3_CN, MeOH, overnight; (II) amine, carboxylic acid, HATU, DMAP, DMF, 0 °C-rt, overnight; (IV) arylboronic acids, XPhos Pd G2, K_3_PO_4_, DMF/EtOH/H_2_O, 95 °C, overnight; (IV) HCl (4 M in 1,4-dioxane), DCM, rt, 2 h.

The synthesis of PLpro inhibitors described in this paper generally followed the routes developed and optimized in our previous report.^37^ The convergent synthesis of PLpro inhibitors is mainly based on reductive amination, amine coupling, and Suzuki–Miyaura cross-coupling reactions. The synthesis of azetidine derivatives **15–40** (Scheme 1) was based upon the synthon **S2** obtained by reductive amination of the aniline with 1-Boc-3-azetidinone, followed by amine coupling. Compounds **21–30** are directly synthesized from **S2** by Suzuki–Miyaura coupling with substituted thienylboronic acids using XPhos Pd G2 as the catalyst followed by Boc deprotection in DCM. Similarly, **S3–S6** were obtained via Suzuki–Miyaura coupling with methyl/formyl substituted thienylboronic acids. Compounds **15–20**, **39**, and **40** were obtained by reductive amination followed by *N*-Boc deprotection. Likewise, amine coupling, and removal of *N*-Boc gave the corresponding derivatives **31–38**.

**Scheme 2:**
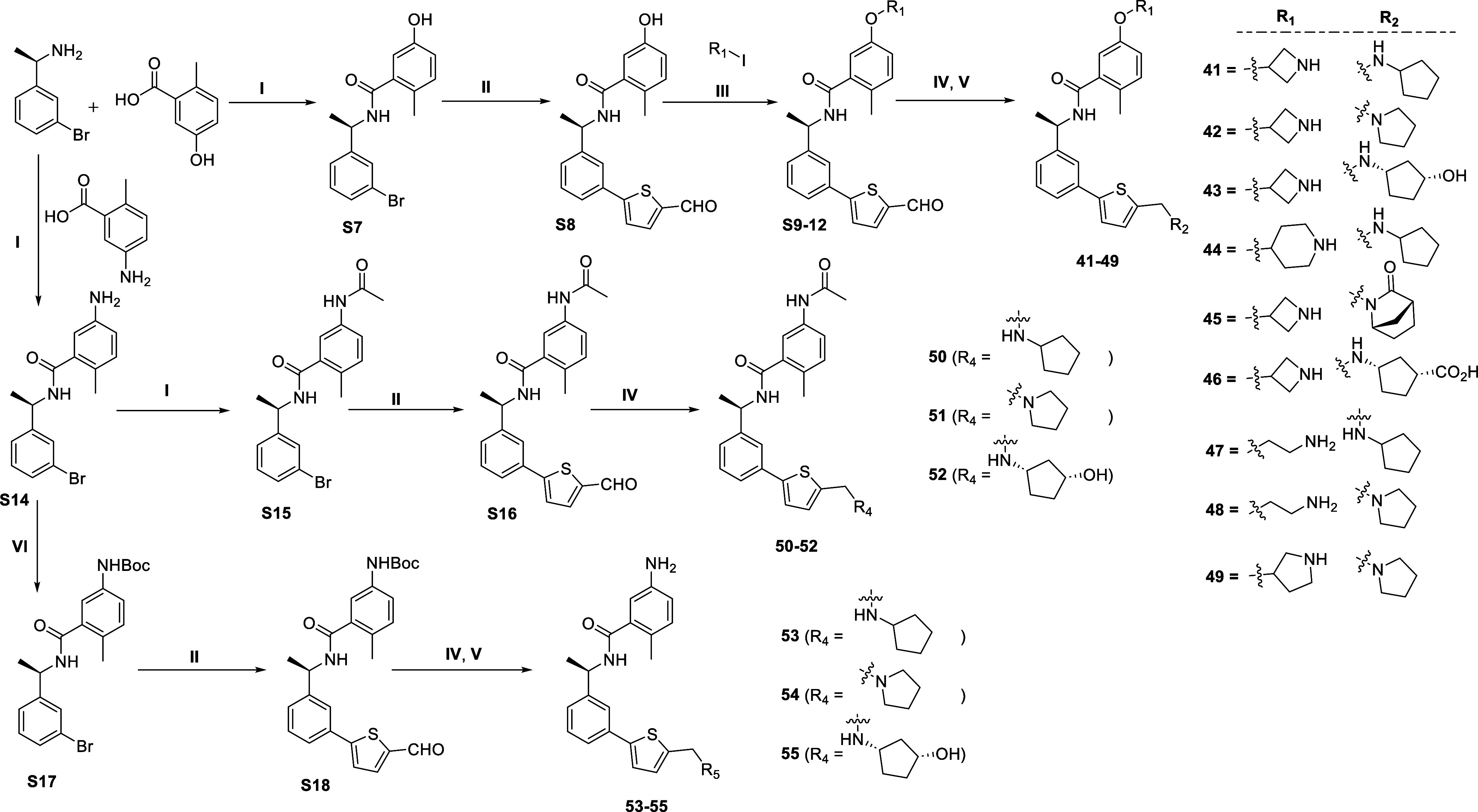
Synthesis of **41–55** Reagents and conditions: (I) amines or carboxylic acids, HATU, DMAP, DMF, 0 °C-rt, overnight; (II) XPhos Pd G2, K_3_PO_4_, DMF/EtOH/H_2_O, 95 °C, overnight; (III) iodo substituted cyclic amines or 2-iodoethan-1-amine, Cs_2_CO_3_, DMF, 100 °C, 12 h; (IV) amines, aldehydes, HOAc, NaBH_3_CN, MeOH, overnight; (V) HCl (4 M in 1,4-dioxane), DCM, rt, 2 h; (VI) (Boc)_2_O, NaOH, H_2_O-1,4-dioxane, rt, 12 h.

Compounds **41–55** were synthesized from **S7**, **S14**, and **S15**, which were obtained by amine coupling of commercially available acids and amines (Scheme 2). Synthon **S7** was subjected to cross-coupling reaction with formyl thioboronic acid to give **S8**, which further reacts with different alicyclic/acyclic iodo compounds via nucleophilic substitution to afford **S9–S12**. Reductive amination between **S9–S12** and substituted cyclic/heterocyclic amines proceeded in moderate yield to provide **41–49** after removal of the *N*-Boc group. Similarly, **50–52** were directly obtained from **S15** by cross-coupling reaction followed by reductive amination. Subsequently, the intermediate **S14** undergoes the *N*-Boc protection and the synthesis of **53–55** were accomplished by the same route described above.

**Scheme 3:**
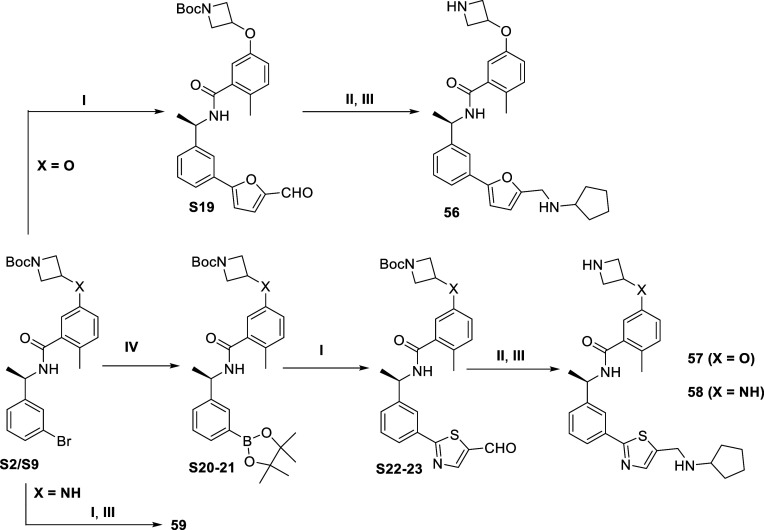
Synthesis of **48–51** Reagents and conditions: (I) XPhos Pd G2, K_3_PO_4_, DMF/EtOH/H_2_O, 95 °C, overnight; (II) amines, aldehydes, HOAc, NaBH_3_CN, MeOH, overnight; (III) HCl (4 M in 1,4-dioxane), DCM, rt, 2 h; (IV) Pd(dppf)Cl_2_, Na_2_CO_3_, 1,4-dioxane, rt-85 °C, 6 h.

Compounds **56–59** were synthesized from **S2/S9** (Scheme 3). The intermediate **S19** was obtained via Suzuki–Miyaura cross-coupling reaction between (5-formylthiophen-2-yl)boronic acid and **S9**. The aldehyde synthon **S19** was readily reacted with amines through a reductive amination, followed by Boc deprotection using 4 M HCl (in 1,4-dioxane) to afford **56** with good yield. Previously synthesized intermediates were used to prepare pinacol esters **S20–S21** by the Miyaura borylation followed by Suzuki–Miyaura cross coupling to form **S22–S23**. These resulting intermediates were subjected to reductive amination to provided the **57–58** after *N*-Boc deprotection. Likewise **59** directly achieved from **S2** by cross-coupling reaction followed by the same *N*-Boc deprotection reaction.

**Scheme 4:**
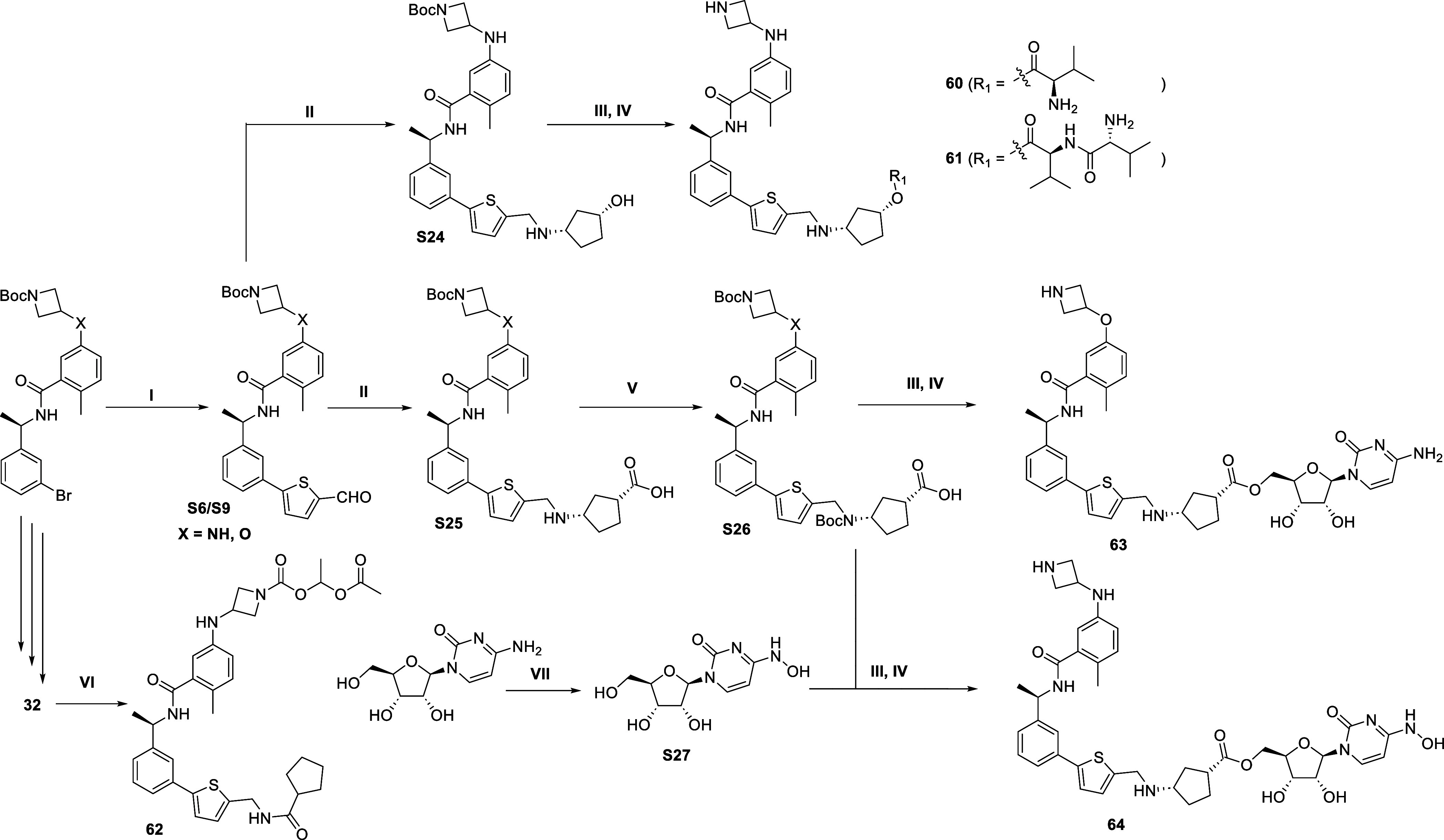
Synthesis of **60–64** Reagents and conditions: (I) XPhos Pd G2, K_3_PO_4_, DMF/EtOH/H_2_O, 95 °C, overnight; (II) amines, aldehydes, HOAc, NaBH_3_CN, MeOH, overnight; (III) amines or carboxylic acids, EDC, DMAP, DMF, 0 °C-rt, overnight; (IV) HCl (4 M in 1,4-dioxane), DCM, rt, 2 h; (V) (Boc)_2_O, NaOH, H_2_O-1,4-dioxane, rt, 12 h; (VI) 1-{[(4-nitrophenoxy)carbonyl]oxy}ethyl acetate, DIPEA, CH_3_CN, rt, overnight; (VII) NH_2_OH·H_2_SO_4_, 70% ipa, 78 °C, 20 h.

The synthetic routes to prepare prodrugs (**60–64**) are illustrated in Scheme 4. Intermediates **S24–S25** were prepared from the previously synthesized synthons **S6** and **S9** via reductive amination reaction where **S24** further reacted with acid to gives the ester through HATU condensation reaction to afford the intermediates for preparation of **60–61** by *N*-Boc deprotection. Compound **62** was obtained from **32** in a single step with good yield. The acid intermediate **S25** was protected by reacting with di*tert*-butyl dicarbonate to provide key intermediate **S26**. Further reaction with cytidine or *N*-hydroxycytidine, **S27**, gave the ester intermediates, which were converted to **63**, **64** by *N*-Boc deprotection in DCM with 4 M HCl (in 1,4-dioxane).

## Summary

The SARS-CoV-2 cysteine proteases, Mpro and PLpro, are excellent therapeutic targets for treatment of future outbreaks of both SARS-CoV-2 VOC and other novel coronaviruses. Inhibition of cysteine proteases by covalent modification of the active site cysteine, is a common approach to inhibition of these enzyme targets and has been successfully translated to the clinic in the form of the Mpro inhibitor nirmatrelvir. Nirmatrelivir compounded with ritonavir is available as the FDA-approved oral antiviral Paxlovid. Theoretically, the drug–drug interactions caused by ritonavir-mediated CYP inhibition should cause adverse effects in the highly drug-treated populations that are most susceptible to morbidity from SARS-CoV-2 infection. A more recently discovered noncovalent Mpro inhibitor that does not require ritonavir coadministration, ensitrelvir,^47^ has not yet gained FDA approval. Dozens of covalent Mpro inhibitors and fewer noncovalent inhibitors have been reported with submicromolar potency in enzyme and cell-based assays, attesting to the success in targeting Mpro, especially with covalent inhibitors.

A similar strategy is ineffective for PLpro owing to the featureless P1 and P2 sites (Gly–Gly recognition). The noncovalent SARS-CoV PLpro inhibitor, GRL0617, lacks sufficient potency for development as an antiviral agent, but cocrystal structures have provided a structural template for design of more potent SARS-CoV-2 PLpro inhibitors. The most notable such approach used GRL0617 as a template to access the active site cysteine via a ≈7 Å linker. This led to covalent inhibitors with double-digit nanomolar potency.^27^ The best of these inhibitors covalently modified the active site cysteine with an ethyl fumarate Michael-acceptor warhead. Unfortunately, the ADME properties of this warhead were reported as unsuitable for study in animal models and further progression.

The absence of a potent, submicromolar, selective inhibitor of SARS-CoV-2 PLpro is a significant weakness in our antiviral armory to tackle SARS-CoV-2 and coronovirus infections in general. An in vivo chemical probe to study PLpro pharmacological inhibition is needed to understand the role of PLpro in regulating innate immune response and host-mediated post-translational modifications in response to viral infection via the actions of PLpro as a DUB/deISGylase. An in vivo chemical probe is also needed to define the benefits of combination therapy with antivirals that target Mpro, RdRp and other viral targets. The effect of PLpro inhibition in both restricting accumulation of viral polypetide products and the disruption of innate immune response may be relevant to “long-COVID”.^48,49^

We previously discovered the BL2-groove, engagement of which contributes to an induced fit mechanism of BL2-loop closure, which blocks access of substrates to the active site of PLpro. Extensive exploration of the SAR for the resulting novel, noncovalent PLpro inhibitors gave little improvement of potency in biochemical assays, with an apparent ceiling at IC_50_ = 100 nM. Furthermore, the most potent inhibitors did not show comparable activity in preventing infection in cell cultures. Most significantly, we demonstrated that one noncovalent PLpro inhibitor, **10**, reduced infection in a mouse model of SARS-CoV-2 infection and was efficacious in cell cultures infected with VOC. This compound is the only reported selective PLpro inhibitor with both efficacy in animal models and submicromolar enzyme inhibition. The observed accumulation of drug in lung tissues at tolerable doses also commends the use of this antiviral agent as a chemical probe for PLpro inhibition in SARS-CoV-2 infection.

## Experimental Section

### Chemical Synthesis

Detailed methods are provided in Supporting Information, including characterization and purity. Unless otherwise specified, reactions were performed under an inert atmosphere of argon and monitored by thin-layer chromatography and/or LCMS. All reagents and solvents were purchased from commercial suppliers (Sigma-Aldrich, Fisher Scientific, Ambeed, Combi-Blocks, Enamine) and used as provided. Synthetic intermediates were purified using a CombiFlash chromatography system on 230–400 mesh silica gel or Shimadzu prep-HPLC system. ^1^H and ^13^C NMR spectra were obtained using Bruker DPX-400, AVANCE-400, 500 and JEOL spectrometer at 500, 400, 125, and 100 MHz, respectively. NMR chemical shifts were described in δ (ppm) using residual solvent peaks as standard. High resolution mass spectral data were measured in-house using a Shimadzu IT-TOF LC/MS for all final compounds. Optical rotations were measured with a PerkinElmer 241 polarimeter operating on the mercury lamp line (546 nm), using a 100 mm path length cell. All compounds submitted for biochemical and biological testing were confirmed to be ≥95% pure by analytical HPLC.

#### (*R*)-5-(Azetidin-3-ylamino)-2-methyl-*N*-(1-(3-(4-methyl-5-(pyrrolidin-1-ylmethyl)thiophen-2-yl)phenyl)ethyl)benzamide (**15**)

To a solution of *tert*-butyl (*R*)-3-((3-((1-(3-(5-formyl-4-methylthiophen-2-yl)phenyl)ethyl)carbamoyl)-4-methylphenyl)amino)azetidine-1-carboxylate (**S3**) (53 mg, 0.1 mmol) and pyrrolidine (10.6 mg, 0.15 mmol) in MeOH, HOAc (100 μL) was added. After stirring at 50 °C 30 min, NaBH_3_CN (19 mg, 0.3 mmol) was added and stirred overnight at room temperature. The reaction mixture was dissolved in ethyl acetate and washed with water and brine solution. After that, the organic layer was dried over Na_2_SO_4_, and the residue was purified by silica gel column chromatography. The obtained product was subjected to general *N*-Boc deprotection procedure with HCl (4 M in dioxane, 100 μL) and DCM (2 mL). The purification by Prep-HPLC afforded the **15** (21 mg, yield 66% for 2 steps) as a white solid. ^1^H NMR (500 MHz, methanol-*d*_*4*_, δ): 8.54 (s, 1H), 7.67 (d, *J* = 1.8 Hz, 1H), 7.52 (dt, *J* = 7.2, 1.8 Hz, 1H), 7.41–7.34 (m, 2H), 7.24 (s, 1H), 7.03 (d, *J* = 8.0 Hz, 1H), 6.60–6.55 (m, 2H), 5.21 (q, *J* = 7.0 Hz, 1H), 4.50 (p, *J* = 7.0 Hz, 1H), 4.35–4.31 (m, 2H), 3.97–3.90 (m, 2H), 3.21 (dt, *J* = 6.9, 3.1 Hz, 4H), 2.32 (s, 3H), 2.21 (s, 3H), 2.05–2.01 (m, 4H), 1.55 (d, *J* = 7.1 Hz, 3H). ^13^C NMR (126 MHz, methanol-*d*_*4*_, δ): 172.57, 146.49, 145.68, 145.51, 141.28, 138.85, 135.26, 132.63, 130.35, 128.40, 127.56, 126.83, 125.68, 125.36, 124.62, 115.55, 112.82, 54.85, 54.83, 54.51, 51.42, 50.52, 47.75, 46.87, 23.94, 22.37, 18.64, 14.26, 9.17. HRMS (ESI) calcd for C_29_H_37_N_4_OS [M + H]^+^, 489.2688; found, 489.2682.

#### (*R*)-5-(Azetidin-3-ylamino)-*N*-(1-(3-(5-((cyclopentylamino)methyl)-4-methylthiophen-2-yl)phenyl)ethyl)-2 Methylbenzamide (**16**)

This compound was obtained by a procedure similar to the preparation of **15** (19 mg, yield 65% for 2 steps). ^1^H NMR (500 MHz, methanol-*d*_*4*_, δ): 8.53 (s, 1H), 7.67 (d, *J* = 2.1 Hz, 1H), 7.52 (dt, *J* = 7.1, 2.0 Hz, 1H), 7.39 (d, *J* = 7.0 Hz, 2H), 7.26 (s, 1H), 7.03 (d, *J* = 8.2 Hz, 1H), 6.61–6.52 (m, 2H), 5.20 (q, *J* = 6.9 Hz, 1H), 4.49 (d, *J* = 8.4 Hz, 1H), 4.38–4.29 (m, 3H), 3.97–3.87 (m, 1H), 3.63 (h, *J* = 6.5 Hz, 1H), 2.33 (s, 3H), 2.20 (s, 3H), 2.18–2.13 (m, 2H), 1.88–1.78 (m, 2H), 1.73–1.63 (m, 4H), 1.55 (d, *J* = 7.1 Hz, 3H). ^13^C NMR (126 MHz, methanol-*d*_*4*_, δ): 172.58, 146.54, 145.94, 145.52, 141.54, 138.83, 135.17, 132.63, 130.38, 127.63, 127.38, 126.86, 125.69, 125.39, 124.71, 115.57, 112.83, 60.03, 54.84, 50.53, 46.88, 43.62, 31.00, 25.08, 22.34, 18.63, 14.04. HRMS (ESI) calcd for C_30_H_39_N_4_OS [M + H]^+^, 503.2845; found, 503.2839.

#### 5-(Azetidin-3-ylamino)-*N*-((*R*)-1-(3-(5-((((1*S*,3*R*)-3-hydroxycyclopentyl)amino)methyl)-4-methylthiophen-2-yl)phenyl)ethyl)-2-methylbenzamide (**17**)

This compound was obtained by a procedure similar to the preparation of **15** (22 mg, yield 68% for 2 steps). ^1^H NMR (500 MHz, methanol-*d*_*4*_, δ): 8.54 (s, 1H), 7.66 (t, *J* = 1.8 Hz, 1H), 7.52 (dt, *J* = 7.1, 1.8 Hz, 1H), 7.41–7.32 (m, 2H), 7.24 (s, 1H), 7.03 (d, *J* = 8.2 Hz, 1H), 6.59–6.53 (m, 2H), 5.20 (q, *J* = 7.0 Hz, 1H), 4.49 (t, *J* = 7.1 Hz, 1H), 4.38–4.28 (m, 5H), 3.92 (dd, *J* = 10.4, 6.5 Hz, 2H), 3.63 (ddt, *J* = 11.1, 7.8, 3.9 Hz, 1H), 2.31 (s, 3H), 2.28–2.21 (m, 1H), 2.22–2.18 (m, 4H), 2.14 (dt, *J* = 13.6, 6.8 Hz, 1H), 1.99–1.90 (m, 1H), 1.83 (dtd, *J* = 17.3, 7.9, 4.4 Hz, 3H), 1.55 (d, *J* = 7.1 Hz, 3H). ^13^C NMR (126 MHz, methanol-*d*_*4*_, δ): 172.59, 170.30, 146.50, 145.52, 140.99, 138.85, 135.29, 132.63, 130.35, 128.46, 127.61, 126.77, 125.68, 125.35, 124.64, 115.59, 112.78, 72.75, 58.75, 54.84, 50.52, 46.91, 43.54, 39.79, 34.46, 28.96, 22.35, 18.63, 14.02. HRMS (ESI) calcd for C_30_H_39_N_4_O_2_S [M + H]^+^, 519.2794; found, 519.2788.

#### 5-(Azetidin-3-ylamino)-*N*-((*R*)-1-(3-(5-((((1*S*,3*R*)-3-hydroxycyclopentyl)amino)methyl)-3-methylthiophen-2-yl)phenyl)ethyl)-2-methylbenzamide (**18**)

This compound was obtained by a procedure similar to the preparation of **15** (17 mg, yield 63% for 2 steps). ^1^H NMR (500 MHz, methanol-*d*_*4*_, δ): 8.54 (s, 1H), 7.52–7.48 (m, 1H), 7.44–7.39 (m, 2H), 7.35 (dt, *J* = 7.0, 1.8 Hz, 1H), 7.08–6.96 (m, 2H), 6.58 (dd, *J* = 8.3, 2.6 Hz, 1H), 6.53 (d, *J* = 2.6 Hz, 1H), 5.22 (q, *J* = 6.9 Hz, 1H), 4.47 (p, *J* = 7.0 Hz, 1H), 4.35–4.27 (m, 3H), 4.23 (s, 2H), 3.90 (dd, *J* = 10.8, 6.8 Hz, 2H), 3.59–3.48 (m, 1H), 2.30 (s, 3H), 2.22–2.17 (m, 4H), 2.15–2.06 (m, 1H), 1.87–1.79 (m, 3H), 1.73 (dt, *J* = 14.2, 4.7 Hz, 1H), 1.55 (d, *J* = 7.1 Hz, 3H). HRMS (ESI) calcd for C_30_H_39_N_4_O_2_S [M + H]^+^, 519.2794; found, 519.2788.

#### (*R*)-5-(Azetidin-3-ylamino)-*N*-(1-(3-(5-((cyclopentylamino)methyl)-3-methylthiophen-2-yl)phenyl)ethyl)-2-methylbenzamide (**19**)

This compound was obtained by a procedure similar to the preparation of **15** (18 mg, yield 65% for 2 steps). ^1^H NMR (500 MHz, methanol-*d*_*4*_, δ): 8.55 (s, 1H), 7.50 (d, *J* = 1.8 Hz, 1H), 7.45–7.39 (m, 2H), 7.35 (dt, *J* = 7.0, 1.9 Hz, 1H), 7.05 (s, 1H), 7.02 (d, *J* = 8.2 Hz, 1H), 6.58 (dd, *J* = 8.2, 2.6 Hz, 1H), 6.54 (d, *J* = 2.5 Hz, 1H), 5.22 (q, *J* = 7.0 Hz, 1H), 4.47 (p, *J* = 7.1 Hz, 1H), 4.33–4.27 (m, 2H), 4.25 (s, 2H), 3.89 (dd, *J* = 10.7, 6.8 Hz, 2H), 3.53–3.44 (m, 1H), 2.30 (s, 3H), 2.20 (s, 3H), 2.13–2.04 (m, 2H), 1.83–1.75 (m, 2H), 1.69–1.57 (m, 2H), 1.55 (d, *J* = 7.1 Hz, 3H). HRMS (ESI) calcd for C_30_H_39_N_4_OS [M + H]^+^, 503.2845; found, 503.2839.

#### (*R*)-5-(Azetidin-3-ylamino)-2-methyl-*N*-(1-(3-(3-methyl-5-(pyrrolidin-1-ylmethyl)thiophen-2-yl)phenyl)ethyl)benzamide (**20**)

This compound was obtained by a procedure similar to the preparation of **15** (22 mg, yield 66% for 2 steps). ^1^H NMR (500 MHz, methanol-*d*_*4*__SPE, δ): 8.53 (s, 1H), 7.51 (d, *J* = 1.9 Hz, 1H), 7.45–7.40 (m, 2H), 7.36 (dt, *J* = 7.0, 1.9 Hz, 1H), 7.07–6.99 (m, 2H), 6.57 (dd, *J* = 8.1, 2.6 Hz, 1H), 6.55 (d, *J* = 2.5 Hz, 1H), 5.22 (q, *J* = 7.0 Hz, 1H), 4.49 (p, *J* = 7.0 Hz, 1H), 4.37–4.29 (m, 2H), 4.28–4.25 (m, 1H), 3.96–3.88 (m, 2H), 3.18–2.90 (m, 5H), 2.30 (s, 3H), 2.20 (s, 3H), 2.05–1.96 (m, 4H), 1.55 (d, *J* = 7.0 Hz, 3H). HRMS (ESI) calcd for C_29_H_37_N_4_OS [M + H]^+^, 489.2688; found, 489.2680.

#### (*R*)-*N*-(1-(3-(5-(Aminomethyl)thiophen-2-yl)phenyl)ethyl)-5-(azetidin-3-ylamino)-2-methylbenzamide (**21**)

A flask fitted with a rubber septum was charged with *tert*-butyl (*R*)-3-((3-((1-(3-bromophenyl)ethyl)carbamoyl)-4-methylphenyl)amino)azetidine-1-carboxylate (**S2**) (48.8 mg, 0.1 mmol), (5-formylthiophen-2-yl)boronic acid (17 mg, 0.15 mmol), XPhos Pd G2 (4 mg, 0.05 mmol), K_3_PO_4_ (53.1 mg, 0.25 mmol), DMF/EtOH/H_2_O (1 mL/1 mL/0.5 mL) and then purged with argon. The mixture was stirred at 95 °C overnight. The reaction mixture was then cooled to room temperature, diluted with ethyl acetate (50 mL), filtered through Celite, and concentrated in vacuo. The purification by flash column chromatography to afford compound **S5** as a white solid. The obtained product was subjected to general *N*-Boc deprotection procedure with HCl (4 M in dioxane, 100 μL) and DCM (2 mL). The purification by Prep-HPLC afforded the **21** (21 mg, yield 66% for 2 steps) as a white solid. ^1^H NMR (500 MHz, methanol-*d*_*4*_, δ): 8.41 (s, 2H), 7.69 (d, *J* = 1.9 Hz, 1H), 7.53 (dt, *J* = 7.0, 1.9 Hz, 1H), 7.37 (s, 0H), 7.35 (d, *J* = 3.7 Hz, 1H), 7.21 (d, *J* = 3.7 Hz, 1H), 7.02 (d, *J* = 8.0 Hz, 1H), 6.57 (d, *J* = 8.2 Hz, 2H), 5.21 (q, *J* = 7.1 Hz, 1H), 4.49 (q, *J* = 7.0 Hz, 1H), 3.94 (dd, *J* = 10.8, 6.7 Hz, 2H), 1.55 (d, *J* = 7.1 Hz, 3H). HRMS (ESI) calcd for C_24_H_29_N_4_OS [M + H]^+^, 421.2062; found, 421.2056.

#### (*R*)-5-(Azetidin-3-ylamino)-*N*-(1-(3-(benzo[*b*]thiophen-2-yl)phenyl)ethyl)-2-methylbenzamide (**22**)

This compound was obtained by a procedure similar to the preparation of **21** (23 mg, yield 69% for 2 steps). ^1^H NMR (400 MHz, methanol-*d*_*4*_, δ): 8.55 (s, 1H), 7.85 (d, *J* = 7.6 Hz, 1H), 7.80 (d, *J* = 7.0 Hz, 2H), 7.71–7.65 (m, 2H), 7.47–7.29 (m, 4H), 7.03 (dd, *J* = 8.2, 2.3 Hz, 1H), 6.57 (dd, *J* = 8.2, 2.6 Hz, 1H), 6.54 (d, *J* = 2.7 Hz, 1H), 5.29–5.21 (m, 1H), 4.45 (q, *J* = 6.7 Hz, 1H), 4.26 (td, *J* = 8.0, 3.9 Hz, 2H), 3.85 (td, *J* = 7.7, 3.6 Hz, 2H), 2.23 (s, 3H), 1.58 (d, *J* = 7.1 Hz, 3H). ^13^C NMR (101 MHz, methanol-*d*_*4*_, δ): 172.66, 146.47, 145.60, 145.20, 142.23, 140.73, 138.90, 135.84, 130.36, 127.31, 126.11, 125.59, 125.27, 124.75, 123.22, 120.86, 115.62, 112.68, 54.95, 50.48, 47.20, 22.43, 18.62. HRMS (ESI) calcd for C_27_H_28_N_3_OS [M + H]^+^, 442.1953; found, 442.1947.

#### (*R*)-5-(Azetidin-3-ylamino)-*N*-(1-(3-(5-cyanothiophen-2-yl)phenyl)ethyl)-2-methylbenzamide (**23**)

This compound was obtained by a procedure similar to the preparation of **21** (25 mg, yield 70% for 2 steps). ^1^H NMR (500 MHz, methanol-*d*_*4*_, δ): 8.56 (s, 1H), 7.77–7.73 (m, 2H), 7.64–7.59 (m, 1H), 7.52–7.45 (m, 3H), 7.05–7.00 (m, 1H), 6.61–6.54 (m, 2H), 5.23 (q, *J* = 6.9 Hz, 1H), 4.47 (p, *J* = 7.0 Hz, 1H), 4.32–4.18 (m, 2H), 3.85 (dd, *J* = 10.7, 6.8 Hz, 2H), 2.21 (s, 3H), 1.56 (d, *J* = 7.1 Hz, 3H). ^13^C NMR (126 MHz, methanol-*d*_*4*_, δ): 172.67, 153.16, 147.00, 145.68, 140.31, 138.82, 133.81, 132.63, 130.72, 128.46, 126.16, 125.69, 125.49, 125.32, 125.22, 125.15, 115.56, 115.06, 112.71, 108.99, 55.03, 55.02, 50.45, 47.34, 22.35, 18.60. HRMS (ESI) calcd for C_24_H_25_N_4_OS [M + H]^+^, 417.1749; found, 417.1743.

#### (*R*)-5-(Azetidin-3-ylamino)-2-methyl-*N*-(1-(3-(4-methylthiophen-2-yl)phenyl)ethyl)benzamide (**24**)

This compound was obtained by a procedure similar to the preparation of **21** (24 mg, yield 69% for 2 steps). ^1^H NMR (500 MHz, methanol-*d*_*4*_, δ): 8.56 (s, 1H), 7.65 (d, *J* = 1.9 Hz, 1H), 7.50 (dt, *J* = 7.6, 1.5 Hz, 1H), 7.35 (t, *J* = 7.6 Hz, 1H), 7.31 (dt, *J* = 7.7, 1.4 Hz, 1H), 7.22 (d, *J* = 1.4 Hz, 1H), 7.03 (d, *J* = 8.2 Hz, 1H), 6.94 (s, 1H), 6.57 (dd, *J* = 8.2, 2.5 Hz, 1H), 6.54 (d, *J* = 2.5 Hz, 1H), 5.21 (q, *J* = 7.0 Hz, 1H), 4.47 (p, *J* = 7.0 Hz, 1H), 4.30 (dd, *J* = 10.5, 7.4 Hz, 2H), 3.89 (dd, *J* = 10.6, 6.6 Hz, 2H), 2.27 (s, 3H), 2.22 (s, 3H), 1.54 (d, *J* = 7.1 Hz, 3H). HRMS (ESI) calcd for C_24_H_28_N_3_OS [M + H]^+^, 406.1953; found, 406.1949.

#### (*R*)-5-(Azetidin-3-ylamino)-2-methyl-*N*-(1-(3-(3-methylthiophen-2-yl)phenyl)ethyl)benzamide (**25**)

This compound was obtained by a procedure similar to the preparation of **21** (22 mg, yield 67% for 2 steps). ^1^H NMR (500 MHz, methanol-*d*_*4*_, δ): 8.55 (s, 1H), 7.50 (d, *J* = 1.8 Hz, 1H), 7.43–7.34 (m, 3H), 7.28 (d, *J* = 5.1 Hz, 1H), 7.03 (d, *J* = 8.2 Hz, 1H), 6.94 (d, *J* = 5.1 Hz, 1H), 6.57 (dd, *J* = 8.2, 2.5 Hz, 1H), 6.53 (d, *J* = 2.5 Hz, 1H), 5.23 (q, *J* = 7.0 Hz, 1H), 4.47 (p, *J* = 6.9 Hz, 1H), 4.31 (dd, *J* = 10.7, 7.5 Hz, 2H), 3.90 (dd, *J* = 10.7, 6.7 Hz, 2H), 2.31 (s, 3H), 2.21 (s, 3H), 1.55 (d, *J* = 7.0 Hz, 3H). HRMS (ESI) calcd for C_24_H_28_N_3_OS [M + H]^+^, 406.1953; found, 406.1947.

#### (*R*)-5-(Azetidin-3-ylamino)-2-methyl-*N*-(1-(3-(5-phenylthiophen-2-yl)phenyl)ethyl)benzamide (**26**)

This compound was obtained by a procedure similar to the preparation of **21** (20 mg, yield 66% for 2 steps). ^1^H NMR (500 MHz, methanol-*d*_*4*_, δ): 8.57 (s, 1H), 7.72 (t, *J* = 1.8 Hz, 1H), 7.67–7.62 (m, 2H), 7.56 (dt, *J* = 7.5, 1.6 Hz, 1H), 7.41–7.31 (m, 6H), 7.28 (t, *J* = 7.4 Hz, 1H), 7.02 (d, *J* = 8.0 Hz, 1H), 6.56 (d, *J* = 8.4 Hz, 2H), 5.23 (q, *J* = 7.0 Hz, 1H), 4.45 (p, *J* = 7.0 Hz, 1H), 4.29–4.22 (m, 2H), 3.86 (dd, *J* = 10.5, 6.5 Hz, 2H), 2.23 (s, 3H), 1.56 (d, *J* = 7.1 Hz, 3H). HRMS (ESI) calcd for C_29_H_30_N_3_OS [M + H]^+^, 468.2110; found, 468.2104.

#### (*R*)-*N*-(1-(3-([2,2′-Bithiophen]-5-yl)phenyl)ethyl)-5-(azetidin-3-ylamino)-2-methylbenzamide (**27**)

This compound was obtained by a procedure similar to the preparation of **21** (26 mg, yield 71% for 2 steps). ^1^H NMR (500 MHz, methanol-*d*_*4*_, δ): 8.56 (s, 1H), 7.69 (t, *J* = 1.9 Hz, 1H), 7.53 (dt, *J* = 7.7, 1.5 Hz, 1H), 7.38 (t, *J* = 7.6 Hz, 1H), 7.35–7.30 (m, 3H), 7.24 (dd, *J* = 3.6, 1.1 Hz, 1H), 7.19 (d, *J* = 3.8 Hz, 1H), 7.07–6.99 (m, 2H), 6.60–6.51 (m, 2H), 5.22 (q, *J* = 7.0 Hz, 1H), 4.47 (p, *J* = 7.1 Hz, 1H), 4.33–4.26 (m, 2H), 3.89 (dd, *J* = 10.6, 6.7 Hz, 2H), 2.23 (s, 3H), 1.55 (d, *J* = 7.0 Hz, 3H). HRMS (ESI) calcd for C_27_H_28_N_3_OS_2_ [M + H]^+^, 474.1674; found, 474.1668.

#### (*R*)-5-(Azetidin-3-ylamino)-2-methyl-*N*-(1-(3-(5-(trifluoromethyl)thiophen-2-yl)phenyl)ethyl)benzamide (**28**)

This compound was obtained by a procedure similar to the preparation of **21** (27 mg, yield 74% for 2 steps). ^1^H NMR (500 MHz, methanol-*d*_*4*_, δ): 8.56 (s, 1H), 7.74–7.71 (m, 1H), 7.58 (d, *J* = 2.5 Hz, 1H), 7.53 (dq, *J* = 3.7, 1.2 Hz, 1H), 7.46–7.42 (m, 3H), 7.03 (d, *J* = 8.1 Hz, 1H), 6.59–6.54 (m, 2H), 5.23 (q, *J* = 7.1 Hz, 1H), 4.46 (p, *J* = 7.1 Hz, 1H), 4.28–4.20 (m, 2H), 3.84 (dd, *J* = 10.4, 6.8 Hz, 2H), 2.21 (s, 3H), 1.56 (d, *J* = 7.1 Hz, 3H). ^13^C NMR (101 MHz, DMSO-*d*_6_, δ): 168.75, 148.27, 146.38, 146.36, 144.69, 144.59, 137.94, 131.97, 131.31 (q, ^3^*J*_C,F_ = 4 Hz, CH-C–CF_3_), 131.05, 129.37, 127.46 (q, ^2^*J*_C,F_ = 38 Hz, C–CF_3_), 126.88, 124.34, 123.98, 123.75, 123.24 (q, ^1^*J*_C,F_ = 269 Hz, CF_3_) 121.17, 113.08, 111.56, 52.74, 48.07, 45.77, 22.55, 18.21.

#### (*R*)-5-(Azetidin-3-ylamino)-*N*-(1-(3-(5-chlorothiophen-2-yl)phenyl)ethyl)-2-methylbenzamide (**29**)

This compound was obtained by a procedure similar to the preparation of **21** (22 mg, yield 66% for 2 steps). ^1^H NMR (500 MHz, methanol-*d*_*4*_, δ): 8.55 (s, 1H), 7.61 (t, *J* = 1.8 Hz, 1H), 7.48 (dt, *J* = 7.3, 1.8 Hz, 1H), 7.41–7.34 (m, 2H), 7.23 (d, *J* = 3.9 Hz, 1H), 7.04 (d, *J* = 8.2 Hz, 1H), 6.98 (d, *J* = 3.9 Hz, 1H), 6.58 (dd, *J* = 8.2, 2.6 Hz, 1H), 6.53 (d, *J* = 2.6 Hz, 1H), 5.21 (q, *J* = 7.0 Hz, 1H), 4.47 (p, *J* = 7.0 Hz, 1H), 4.34–4.27 (m, 2H), 3.89 (dd, *J* = 10.7, 6.7 Hz, 2H), 2.21 (s, 3H), 1.54 (d, *J* = 7.1 Hz, 3H). HRMS (ESI) calcd for C_23_H_25_ClN_3_OS [M + H]^+^, 426.1407; found, 426.1401.

#### (*R*)-5-(Azetidin-3-ylamino)-2-methyl-*N*-(1-(3-(thieno[3,2-*b*]thiophen-2-yl)phenyl)ethyl)benzamide (**30**)

This compound was obtained by a procedure similar to the preparation of **21** (17 mg, yield 64% for 2 steps). ^1^H NMR (500 MHz, methanol-*d*_*4*_, δ): 8.56 (s, 1H), 7.73 (t, *J* = 1.8 Hz, 1H), 7.68–7.63 (m, 1H), 7.59 (dt, *J* = 7.6, 1.6 Hz, 1H), 7.49 (d, *J* = 5.3 Hz, 1H), 7.40 (t, *J* = 7.6 Hz, 1H), 7.36 (dt, *J* = 7.7, 1.5 Hz, 1H), 7.31 (d, *J* = 5.3 Hz, 1H), 7.03 (d, *J* = 8.2 Hz, 1H), 6.57 (dd, *J* = 8.1, 2.6 Hz, 1H), 6.54 (d, *J* = 2.5 Hz, 1H), 5.24 (q, *J* = 7.0 Hz, 1H), 4.45 (p, *J* = 7.1 Hz, 1H), 4.24 (ddd, *J* = 10.2, 5.5, 3.8 Hz, 2H), 3.83 (ddd, *J* = 10.9, 6.7, 1.7 Hz, 2H), 2.23 (s, 3H), 1.56 (d, *J* = 7.1 Hz, 3H). ^13^C NMR (126 MHz, methanol-*d*_*4*_, δ): 172.66, 170.36, 147.30, 146.45, 145.65, 141.53, 139.85, 138.93, 136.43, 132.61, 130.34, 128.41, 126.77, 125.54, 125.43, 124.52, 120.57, 116.63, 115.61, 112.64, 55.06, 50.52, 47.40, 22.38, 18.63. HRMS (ESI) calcd for C_25_H_26_N_3_OS_2_ [M + H]^+^, 448.1517; found, 448.1511.

#### (*R*)-5-(Azetidin-3-ylamino)-*N*-(1-(3-(5-(cyclopropanecarboxamidomethyl)thiophen-2-yl)phenyl)ethyl)-2-methylbenzamide (**31**)

Compound **31** was obtained by using the regular esterification reaction of **S5** with HATU followed by *N*-Boc deprotection in 4 M HCl (in 1,4-dioxane) (18 mg, yield 65% for 2 steps). ^1^H NMR (500 MHz, methanol-*d*_*4*_, δ): 8.53 (s, 1H), 7.65 (s, 1H), 7.55–7.44 (m, 2H), 7.40–7.30 (m, 2H), 7.27–7.22 (m, 1H), 6.97 (dd, *J* = 14.9, 5.8 Hz, 2H), 6.61–6.53 (m, 2H), 5.26–5.17 (m, 1H), 4.54 (s, 2H), 4.11–4.03 (m, 1H), 3.96–3.87 (m, 1H), 3.73–3.64 (m, 1H), 3.63–3.53 (m, 1H), 3.28–3.19 (m, 3H), 2.44–2.40 (m, 1H), 2.20 (s, 3H), 1.59–1.57 (m, 1H), 1.54 (d, *J* = 7.1 Hz, 3H), 0.91–0.87 (m, 2H), 0.81–0.74 (m, 2H). HRMS (ESI) calcd for C_28_H_33_N_4_O_2_S [M + H]^+^, 489.2324; found, 489.2318.

#### (*R*)-5-(Azetidin-3-ylamino)-*N*-(1-(3-(5-(cyclopentanecarboxamidomethyl)thiophen-2-yl)phenyl)ethyl)-2-methylbenzamide (**32**)

This compound was obtained by a procedure similar to the preparation of **31** (21 mg, yield 66% for 2 steps). ^1^H NMR (500 MHz, methanol-*d*_*4*_, δ): 8.51 (s, 1H), 7.63 (d, *J* = 1.8 Hz, 1H), 7.50 (dt, *J* = 7.7, 1.5 Hz, 1H), 7.36 (t, *J* = 7.6 Hz, 1H), 7.31 (dt, *J* = 7.9, 1.5 Hz, 1H), 7.23 (d, *J* = 3.5 Hz, 1H), 7.03 (d, *J* = 8.3 Hz, 1H), 6.94 (d, *J* = 3.6 Hz, 1H), 6.59 (dd, *J* = 8.2, 2.5 Hz, 1H), 6.52 (d, *J* = 2.5 Hz, 1H), 5.20 (q, *J* = 7.0 Hz, 1H), 4.52 (s, 2H), 4.48 (q, *J* = 7.0 Hz, 1H), 4.35 (dd, *J* = 10.9, 7.5 Hz, 2H), 3.93 (dd, *J* = 11.0, 6.8 Hz, 2H), 2.70–2.62 (m, 1H), 2.22 (s, 3H), 1.91–1.82 (m, 2H), 1.79–1.69 (m, 4H), 1.65–1.56 (m, 2H), 1.54 (d, *J* = 7.0 Hz, 3H). ^13^C NMR (126 MHz, methanol-*d*_*4*_, δ): 179.03, 172.59, 146.33, 145.46, 144.85, 143.15, 138.95, 136.06, 132.65, 130.21, 127.58, 126.44, 125.74, 125.19, 124.19, 123.84, 115.75, 112.51, 54.95, 54.92, 50.48, 46.90, 46.45, 39.15, 31.45, 31.43, 27.02, 22.42, 18.63. HRMS (ESI) calcd for C_30_H_37_N_4_O_2_S [M + H]^+^, 517.2637; found, 517.2631.

#### (*R*)-*N*-((5-(3-(1-(5-(Azetidin-3-ylamino)-2-methylbenzamido)ethyl)phenyl)thiophen-2-yl)methyl)tetrahydro-2*H*-pyran-4-carboxamide (**33**)

This compound was obtained by a procedure similar to the preparation of **31** (19 mg, yield 66% for 2 steps). ^1^H NMR (500 MHz, methanol-*d*_*4*_, δ): 8.54 (s, 1H), 7.63 (d, *J* = 6.8 Hz, 1H), 7.49 (t, *J* = 7.4 Hz, 1H), 7.36 (td, *J* = 7.6, 1.8 Hz, 1H), 7.31 (d, *J* = 7.8 Hz, 1H), 7.23 (d, *J* = 3.6 Hz, 1H), 7.07–6.97 (m, 1H), 6.95 (d, *J* = 3.6 Hz, 1H), 6.61–6.56 (m, 1H), 6.53 (dd, *J* = 17.5, 2.3 Hz, 1H), 5.20 (q, *J* = 7.0 Hz, 1H), 4.53 (s, 2H), 4.47 (q, *J* = 7.0 Hz, 1H), 4.34 (dd, *J* = 10.8, 7.4 Hz, 1H), 4.00–3.88 (m, 3H), 3.43 (td, *J* = 11.7, 2.4 Hz, 2H), 3.30–3.19 (m, 2H), 2.53–2.44 (m, 1H), 1.84–1.74 (m, 2H), 1.77 (dd, *J* = 14.1, 10.1 Hz, 2H), 1.74–1.63 (m, 2H), 1.54 (d, *J* = 7.1 Hz, 3H). HRMS (ESI) calcd for C_30_H_37_N_4_O_3_S [M + H]^+^, 533.2586; found, 533.2580.

#### *N*-((5-(3-((*R*)-1-(5-(Azetidin-3-ylamino)-2-methylbenzamido)ethyl)phenyl)thiophen-2-yl)methyl)tetrahydrofuran-2-carboxamide (**34**)

This compound was obtained by a procedure similar to the preparation of **31** (23 mg, yield 67% for 2 steps). ^1^H NMR (500 MHz, methanol-*d*_*4*_, δ): 8.51 (s, 1H), 7.63 (d, *J* = 6.8 Hz, 1H), 7.53–7.47 (m, 1H), 7.36 (td, *J* = 7.6, 2.1 Hz, 1H), 7.31 (d, *J* = 7.8 Hz, 1H), 7.23 (d, *J* = 3.6 Hz, 1H), 7.04 (d, *J* = 8.3 Hz, 1H), 7.01–6.94 (m, 2H), 6.61–6.56 (m, 1H), 6.56–6.49 (m, 1H), 5.20 (q, *J* = 6.8 Hz, 1H), 4.61–4.51 (m, 3H), 4.48 (q, *J* = 7.0 Hz, 1H), 4.34 (q, *J* = 8.1 Hz, 3H), 3.99 (q, *J* = 7.0 Hz, 1H), 3.93 (dd, *J* = 11.0, 6.7 Hz, 1H), 3.86 (q, *J* = 7.2 Hz, 1H), 2.21 (d, *J* = 14.4 Hz, 4H), 2.03–1.95 (m, 0H), 1.94–1.84 (m, *J* = 6.1 Hz, 2H), 1.54 (d, *J* = 7.1 Hz, 3H). HRMS (ESI) calcd for C_29_H_35_N_4_O_3_S [M + H]^+^, 519.2430; found, 519.2427.

#### (*R*)-*N*-((5-(3-(1-(5-(Azetidin-3-ylamino)-2-methylbenzamido)ethyl)phenyl)thiophen-2-yl)methyl)piperidine-4-carboxamide (**35**)

This compound was obtained by a procedure similar to the preparation of **31** (20 mg, yield 65% for 2 steps). ^1^H NMR (400 MHz, methanol-*d*_*4*_, δ): 8.53 (s, 1H), 7.63 (s, 1H), 7.48 (d, *J* = 7.4 Hz, 1H), 7.40–7.29 (m, 2H), 7.24 (d, *J* = 3.6 Hz, 1H), 7.03 (d, *J* = 8.3 Hz, 1H), 6.97 (d, *J* = 3.7 Hz, 1H), 6.61–6.51 (m, 2H), 5.49 (d, *J* = 1.6 Hz, 1H), 5.20 (d, *J* = 7.2 Hz, 1H), 4.54 (s, 2H), 4.48 (t, *J* = 7.1 Hz, 1H), 4.36–4.27 (m, 1H), 3.93–3.86 (m, 1H), 3.50–3.37 (m, 2H), 3.16–3.10 (m, 1H), 2.99 (t, *J* = 12.4 Hz, 2H), 2.60–2.51 (m, 1H), 2.21 (s, 3H), 2.01 (d, *J* = 14.6 Hz, 2H), 1.91 (dd, *J* = 14.7, 10.8 Hz, 2H), 1.54 (d, *J* = 6.4 Hz, 3H). HRMS (ESI) calcd for C_30_H_38_N_5_O_2_S [M + H]^+^, 532.2746; found, 532.2740.

#### (*R*)-*N*-((5-(3-(1-(5-(Azetidin-3-ylamino)-2-methylbenzamido)ethyl)phenyl)thiophen-2-yl)methyl)azetidine-3-carboxamide (**36**)

This compound was obtained by a procedure similar to the preparation of **31** (17 mg, yield 62% for 2 steps). ^1^H NMR (500 MHz, methanol-*d*_*4*_, δ): 8.54 (s, 1H), 7.63 (s, 2H), 7.52–7.47 (m, 1H), 7.35 (dt, *J* = 15.5, 7.7 Hz, 2H), 7.03 (d, *J* = 8.3 Hz, 1H), 6.99 (d, *J* = 3.7 Hz, 1H), 6.58 (dd, *J* = 8.2, 2.6 Hz, 1H), 6.54 (d, *J* = 2.5 Hz, 1H), 5.20 (q, *J* = 7.0 Hz, 1H), 4.58 (s, 2H), 4.48 (p, *J* = 7.0 Hz, 1H), 4.31 (dd, *J* = 10.8, 7.6 Hz, 2H), 4.20–4.08 (m, 3H), 3.89 (dd, *J* = 10.9, 6.7 Hz, 2H), 3.68–3.59 (m, 1H), 2.21 (s, 3H), 1.54 (d, *J* = 7.1 Hz, 3H). HRMS (ESI) calcd for C_28_H_34_N_5_O_2_S [M + H]^+^, 504.2433; found, 504.2428.

#### *N*-((1*R*)-1-(3-(5-((3-Aminocyclopentane-1-carboxamido)methyl)thiophen-2-yl)phenyl)ethyl)-5-(azetidin-3-ylamino)-2-methylbenzamide (**37**)

This compound was obtained by a procedure similar to the preparation of **31** (18 mg, yield 64% for 2 steps). ^1^H NMR (500 MHz, methanol-*d*_*4*_, δ): 8.55 (s, 2H), 7.63 (q, *J* = 2.1 Hz, 1H), 7.49 (dt, *J* = 7.5, 1.6 Hz, 1H), 7.38–7.30 (m, 2H), 7.24 (t, *J* = 3.6 Hz, 1H), 7.03 (d, *J* = 8.2 Hz, 1H), 6.96 (dd, *J* = 6.7, 3.6 Hz, 1H), 6.56 (s, 0H), 6.54 (d, *J* = 2.4 Hz, 1H), 5.20 (q, *J* = 7.1 Hz, 1H), 4.54 (dd, *J* = 9.0, 3.1 Hz, 2H), 4.47 (p, *J* = 7.0 Hz, 1H), 4.28 (ddd, *J* = 10.9, 6.1, 2.6 Hz, 2H), 3.90–3.83 (m, 2H), 3.71 (q, *J* = 5.8 Hz, 1H), 3.01–2.92 (m, 1H), 2.26–2.16 (m, 4H), 2.15–2.04 (m, 1H), 2.01–1.93 (m, 1H), 1.93–1.78 (m, 2H), 1.54 (d, *J* = 7.1 Hz, 3H). HRMS (ESI) calcd for C_30_H_38_N_5_O_2_S [M + H]^+^, 532.2746; found, 532.2742.

#### (*S*)-*N*-((5-(3-((*R*)-1-(5-(Azetidin-3-ylamino)-2-methylbenzamido)ethyl)phenyl)thiophen-2-yl)methyl)pyrrolidine-2-carboxamide (**38**)

This compound was obtained by a procedure similar to the preparation of **31** (19 mg, yield 63% for 2 steps). ^1^H NMR (500 MHz, methanol-*d*_*4*_, δ): 8.54 (s, 1H), 7.63 (d, *J* = 1.9 Hz, 1H), 7.50 (dt, *J* = 7.6, 1.6 Hz, 1H), 7.37 (t, *J* = 7.6 Hz, 1H), 7.33 (dt, *J* = 7.7, 1.5 Hz, 1H), 7.25 (d, *J* = 3.6 Hz, 1H), 7.04 (d, *J* = 8.3 Hz, 1H), 6.99 (d, *J* = 3.7 Hz, 1H), 6.59 (dd, *J* = 8.3, 2.6 Hz, 1H), 6.53 (d, *J* = 2.6 Hz, 1H), 5.20 (q, *J* = 7.0 Hz, 1H), 4.65–4.54 (m, 2H), 4.49 (p, *J* = 7.1 Hz, 1H), 4.39–4.30 (m, 2H), 4.02 (dd, *J* = 8.4, 6.0 Hz, 1H), 3.95–3.88 (m, 2H), 3.28–3.20 (m, 1H), 3.16 (dt, *J* = 11.5, 6.7 Hz, 1H), 2.35–2.24 (m, 1H), 2.21 (s, 3H), 1.99–1.87 (m, 3H), 1.54 (d, *J* = 7.0 Hz, 3H). HRMS (ESI) calcd for C_29_H_36_N_5_O_2_S [M + H]^+^, 518.2590; found, 518.2584.

#### (1*R*,3*S*)-3-(((5-(3-((*R*)-1-(5-(Azetidin-3-ylamino)-2-methylbenzamido)ethyl)phenyl)thiophen-2-yl)methyl)amino)cyclopentane-1-carboxylic Acid (**39**)

This compound was obtained by a procedure similar to the preparation of **15** (16 mg, yield 61% for 2 steps). ^1^H NMR (500 MHz, methanol-*d*_*4*_, δ): 8.52 (s, 1H), 7.69 (d, *J* = 1.8 Hz, 1H), 7.55 (dt, *J* = 7.6, 1.6 Hz, 1H), 7.40 (t, *J* = 7.5 Hz, 1H), 7.38–7.35 (m, 2H), 7.27 (d, *J* = 3.8 Hz, 1H), 7.04 (d, *J* = 8.3 Hz, 1H), 6.61 (dd, *J* = 8.3, 2.5 Hz, 1H), 6.45 (d, *J* = 2.5 Hz, 1H), 5.22 (q, *J* = 7.0 Hz, 1H), 4.48–4.40 (m, 2H), 4.36 (d, *J* = 14.0 Hz, 1H), 4.33–4.24 (m, 2H), 3.90 (ddd, *J* = 10.9, 6.3, 4.4 Hz, 2H), 3.76–3.70 (m, 1H), 2.97–2.90 (m, 1H), 2.30–2.18 (m, 4H), 2.13 (ddd, *J* = 12.0, 10.3, 5.1 Hz, 2H), 2.06–1.94 (m, 3H), 1.55 (d, *J* = 7.0 Hz, 3H). ^13^C NMR (126 MHz, methanol-*d*_*4*_, δ): 185.54, 172.59, 169.97, 148.01, 146.65, 145.56, 138.84, 135.38, 134.01, 132.69, 132.35, 130.38, 127.37, 125.73, 125.56, 124.76, 124.24, 116.15, 112.14, 61.38, 54.61, 54.43, 50.41, 47.05, 46.97, 44.72, 34.54, 31.20, 30.52, 22.41, 18.59. HRMS (ESI) calcd for C_30_H_37_N_4_O_3_S [M + H]^+^, 533.2586; found, 533.2579.

#### Ethyl (1*R*,3*S*)-3-(((5-(3-((*R*)-1-(5-(Azetidin-3-ylamino)-2-methylbenzamido)ethyl)phenyl) Thiophen-2-yl)methyl)amino)cyclopentane-1-carboxylate (**40**)

This compound was obtained by a procedure similar to the preparation of **15** (19 mg, yield 66% for 2 steps). ^1^H NMR (500 MHz, methanol-*d*_*4*_, δ): 8.53 (s, 1H), 7.69 (t, *J* = 1.8 Hz, 1H), 7.55 (dt, *J* = 7.4, 1.7 Hz, 1H), 7.42–7.33 (m, 3H), 7.17 (d, *J* = 3.7 Hz, 1H), 7.05 (d, *J* = 8.3 Hz, 1H), 6.59 (dd, *J* = 8.1, 2.6 Hz, 1H), 6.55 (d, *J* = 2.6 Hz, 1H), 5.22 (q, *J* = 7.0 Hz, 1H), 4.50 (p, *J* = 6.9 Hz, 1H), 4.35 (dd, *J* = 11.0, 8.0 Hz, 2H), 4.25 (s, 2H), 4.16 (q, *J* = 7.1 Hz, 2H), 3.97–3.90 (m, 2H), 3.51 (q, *J* = 7.6 Hz, 1H), 2.91 (p, *J* = 8.2 Hz, 1H), 2.37 (dt, *J* = 14.2, 7.5 Hz, 1H), 2.22 (s, 3H), 2.11 (dq, *J* = 13.6, 7.4 Hz, 1H), 2.03–1.99 (m, 2H), 1.86 (dt, *J* = 13.1, 8.6 Hz, 1H), 1.73 (dq, *J* = 15.4, 7.6 Hz, 1H), 1.56 (d, *J* = 7.1 Hz, 3H), 1.27 (t, *J* = 7.1 Hz, 3H). HRMS (ESI) calcd for C_32_H_41_N_4_O_3_S [M + H]^+^, 561.2899; found, 561.2893.

#### (*R*)-5-(Azetidin-3-yloxy)-*N*-(1-(3-(5-((cyclopentylamino)methyl)thiophen-2-yl)phenyl)ethyl)-2-methylbenzamide (**41**)

This compound was obtained by a procedure similar to the preparation of **15** (25 mg, yield 71% for 2 steps). ^1^H NMR (500 MHz, methanol-*d*_*4*_, δ): 7.69 (t, *J* = 1.9 Hz, 1H), 7.55 (dt, *J* = 7.3, 1.7 Hz, 1H), 7.44–7.35 (m, 3H), 7.25 (d, *J* = 3.6 Hz, 1H), 7.19 (dd, *J* = 8.4, 4.6 Hz, 1H), 6.86–6.77 (m, 2H), 5.22 (q, *J* = 7.1 Hz, 1H), 5.13 (dh, *J* = 10.1, 5.4 Hz, 1H), 4.45 (dd, *J* = 11.6, 6.6 Hz, 2H), 4.39 (d, *J* = 5.7 Hz, 2H), 4.07 (dd, *J* = 11.6, 4.7 Hz, 2H), 3.61–3.53 (m, 1H), 2.26 (s, 3H), 2.19–2.10 (m, 2H), 1.87–1.77 (m, 2H), 1.73–1.61 (m, 4H), 1.56 (d, *J* = 7.1 Hz, 3H). ^13^C NMR (126 MHz, methanol-*d*_*4*_, δ): 155.36, 147.83, 146.49, 139.38, 135.30, 134.31, 133.18, 132.28, 130.48, 130.02, 126.88, 125.63, 124.88, 124.70, 117.15, 114.67, 69.39, 59.87, 54.44, 50.64, 45.66, 31.04, 25.04, 22.30, 18.72. HRMS (ESI) calcd for C_29_H_36_N_3_O_2_S [M + H]^+^, 490.2528; found, 490.2522.

#### (*R*)-5-(Azetidin-3-yloxy)-2-methyl-*N*-(1-(3-(5-(pyrrolidin-1-ylmethyl)thiophen-2-yl)phenyl)ethyl)benzamide (**42**)

This compound was obtained by a procedure similar to the preparation of **15** (26 mg, yield 72% for 2 steps). ^1^H NMR (500 MHz, methanol-*d*_*4*_, δ): 8.52 (s, 1H), 7.69 (t, *J* = 1.8 Hz, 1H), 7.56 (dt, *J* = 7.3, 1.8 Hz, 1H), 7.44–7.36 (m, 3H), 7.26 (d, *J* = 3.7 Hz, 1H), 7.18 (t, *J* = 7.6 Hz, 1H), 6.82 (dtd, *J* = 8.6, 5.8, 2.7 Hz, 2H), 5.22 (q, *J* = 7.1 Hz, 1H), 5.12 (dtt, *J* = 15.4, 6.4, 4.5 Hz, 1H), 4.52–4.45 (m, 4H), 4.13–4.09 (m, 2H), 3.27 (dt, *J* = 6.9, 4.0 Hz, 4H), 2.27 (s, 3H), 2.07–2.03 (m, 4H), 1.56 (dd, *J* = 7.2, 1.9 Hz, 3H). HRMS (ESI) calcd for C_28_H_34_N_3_O_2_S [M + H]^+^, 476.2372; found, 476.2366.

#### 5-(Azetidin-3-yloxy)-*N*-((*R*)-1-(3-(5-((((1*S*,3*R*)-3-hydroxycyclopentyl)amino)methyl) Thiophen-2-yl)phenyl)ethyl)-2-methylbenzamide (**43**)

This compound was obtained by a procedure similar to the preparation of **15** (24 mg, yield 69% for 2 steps). ^1^H NMR (500 MHz, methanol-*d*_*4*_, δ): 8.52 (s, 1H), 7.69 (t, *J* = 1.9 Hz, 1H), 7.55 (dt, *J* = 7.4, 1.7 Hz, 1H), 7.44–7.35 (m, 3H), 7.24 (d, *J* = 3.7 Hz, 1H), 7.19 (d, *J* = 8.3 Hz, 1H), 6.84 (dd, *J* = 8.3, 2.8 Hz, 1H), 6.81 (d, *J* = 2.7 Hz, 1H), 5.22 (d, *J* = 7.1 Hz, 1H), 5.13 (ddd, *J* = 6.6, 4.8, 1.7 Hz, 1H), 4.50–4.43 (m, 2H), 4.38 (s, 2H), 4.34–4.30 (m, 1H), 4.12–4.04 (m, 2H), 3.67–3.57 (m, 1H), 2.26 (s, 3H), 2.25–2.19 (m, 1H), 2.18–2.10 (m, 1H), 1.98–1.89 (m, 1H), 1.86–1.77 (m, 3H), 1.56 (d, *J* = 7.0 Hz, 3H). ^13^C NMR (126 MHz, methanol-*d*_*4*_, δ): 170.17, 153.91, 146.40, 145.07, 137.99, 133.91, 131.78, 130.85, 129.07, 128.66, 125.46, 124.22, 123.46, 123.29, 115.76, 113.26, 71.24, 67.88, 57.05, 53.02, 49.23, 44.05, 38.28, 33.05, 27.32, 20.90, 17.31. HRMS (ESI) calcd for C_29_H_36_N_3_O_3_S [M + H]^+^, 506.2477; found, 506.2471.

#### (*R*)-*N*-(1-(3-(5-((Cyclopentylamino)methyl)thiophen-2-yl)phenyl)ethyl)-2-methyl-5-(piperidin-4-yloxy)benzamide (**44**)

This compound was obtained by a procedure similar to the preparation of **15** (22 mg, yield 67% for 2 steps). ^1^H NMR (500 MHz, methanol-*d*_*4*_, δ): 8.52 (s, 1H), 7.68 (d, *J* = 1.9 Hz, 1H), 7.54 (dt, *J* = 7.5, 1.7 Hz, 1H), 7.39 (d, *J* = 7.4 Hz, 1H), 7.37 (d, *J* = 7.9 Hz, 1H), 7.34 (d, *J* = 3.6 Hz, 1H), 7.18 (d, *J* = 8.4 Hz, 1H), 7.16 (d, *J* = 3.8 Hz, 1H), 6.99 (dd, *J* = 8.4, 2.7 Hz, 1H), 6.96 (d, *J* = 2.7 Hz, 1H), 5.22 (q, *J* = 7.1 Hz, 1H), 4.68 (tt, *J* = 6.6, 3.3 Hz, 1H), 4.26 (s, 2H), 3.42–3.33 (m, 2H), 3.18 (ddd, *J* = 12.9, 6.7, 4.1 Hz, 2H), 3.04 (d, *J* = 6.4 Hz, 4H), 2.27 (s, 3H), 2.13 (ddt, *J* = 13.2, 8.0, 3.7 Hz, 2H), 2.04–1.95 (m, 7H), 1.56 (d, *J* = 7.0 Hz, 3H).

#### 5-(Azetidin-3-yloxy)-2-methyl-*N*-((*R*)-1-(3-(5-(((1*S*,4*R*)-3-oxo-2-azabicyclo[2.2.1]heptan-2-yl)methyl)thiophen-2-yl)phenyl)ethyl)benzamide (**45**)

This compound was obtained by a procedure similar to the preparation of **15** (18 mg, yield 65% for 2 steps). ^1^H NMR (500 MHz, methanol-*d*_*4*_, δ): 8.55 (s, 1H), 7.67–7.62 (m, 1H), 7.52 (dt, *J* = 7.6, 1.6 Hz, 1H), 7.37 (t, *J* = 7.6 Hz, 1H), 7.33 (dt, *J* = 7.7, 1.5 Hz, 1H), 7.26 (d, *J* = 3.6 Hz, 1H), 7.18 (d, *J* = 8.4 Hz, 1H), 7.01 (d, *J* = 3.6 Hz, 1H), 6.82 (dd, *J* = 8.4, 2.7 Hz, 1H), 6.79 (d, *J* = 2.7 Hz, 1H), 5.21 (q, *J* = 7.0 Hz, 1H), 5.10 (tt, *J* = 6.4, 4.9 Hz, 1H), 4.65 (d, *J* = 15.6 Hz, 1H), 4.40 (d, *J* = 15.6 Hz, 1H), 4.38–4.31 (m, 2H), 4.03–3.97 (m, 2H), 3.96 (t, *J* = 1.8 Hz, 1H), 2.81–2.77 (m, 1H), 1.97–1.88 (m, 1H), 1.83 (dq, *J* = 9.1, 2.1 Hz, 1H), 1.76 (dddd, *J* = 11.7, 9.7, 3.7, 2.2 Hz, 1H), 1.55 (d, *J* = 7.1 Hz, 4H), 1.53–1.46 (m, 2H), 1.44 (dt, *J* = 9.6, 1.4 Hz, 1H). HRMS (ESI) calcd for C_30_H_34_N_3_O_3_S [M + H]^+^, 516.2321; found, 516.2315.

#### (1*R*,3*S*)-3-(((5-(3-((*R*)-1-(5-(Azetidin-3-yloxy)-2-methylbenzamido)ethyl)phenyl)thiophen-2-yl)methyl)amino)cyclopentane-1-carboxylic Acid (**46**)

This compound was obtained by a procedure similar to the preparation of **15** (20 mg, yield 65% for 2 steps). ^1^H NMR (500 MHz, methanol-*d*_*4*_, δ): 8.53 (s, 1H), 7.68 (t, *J* = 1.8 Hz, 1H), 7.55 (dt, *J* = 7.5, 1.7 Hz, 1H), 7.40 (t, *J* = 7.6 Hz, 1H), 7.38–7.35 (m, 2H), 7.27 (d, *J* = 3.7 Hz, 1H), 7.20 (d, *J* = 8.5 Hz, 1H), 6.86 (dd, *J* = 8.4, 2.7 Hz, 1H), 6.72 (d, *J* = 2.7 Hz, 1H), 5.22 (q, *J* = 7.0 Hz, 1H), 5.10 (tt, *J* = 6.5, 4.8 Hz, 1H), 4.48–4.38 (m, 3H), 4.35 (d, *J* = 14.0 Hz, 1H), 4.10–4.02 (m, 2H), 3.74 (ddt, *J* = 6.5, 4.7, 2.2 Hz, 1H), 2.93 (tdd, *J* = 8.7, 3.9, 2.0 Hz, 1H), 2.30 (s, 3H), 2.28–2.23 (m, 1H), 2.12 (ddd, *J* = 11.6, 9.4, 4.8 Hz, 2H), 2.05–1.96 (m, 3H), 1.55 (d, *J* = 7.1 Hz, 3H).

#### (*R*)-5-(2-Aminoethoxy)-*N*-(1-(3-(5-((cyclopentylamino)methyl)thiophen-2-yl)phenyl)ethyl)-2-methylbenzamide (**47**)

This compound was obtained by a procedure similar to the preparation of **15** (21 mg, yield 67% for 2 steps). ^1^H NMR (500 MHz, methanol-*d*_*4*_, δ): 8.53 (s, 1H), 7.69 (d, *J* = 1.8 Hz, 1H), 7.55 (dt, *J* = 7.1, 1.8 Hz, 1H), 7.44–7.35 (m, 3H), 7.23 (dd, *J* = 8.0, 3.6 Hz, 1H), 7.19 (d, *J* = 8.4 Hz, 1H), 6.99 (dd, *J* = 8.3, 2.8 Hz, 1H), 6.96 (d, *J* = 2.7 Hz, 1H), 5.23 (q, *J* = 6.9 Hz, 1H), 4.36 (d, *J* = 15.7 Hz, 2H), 4.20 (t, *J* = 5.0 Hz, 2H), 3.54 (h, *J* = 7.5 Hz, 1H), 2.27 (s, 2H), 2.12 (dt, *J* = 12.2, 8.4 Hz, 2H), 1.81 (d, *J* = 6.7 Hz, 2H), 1.72–1.59 (m, 4H), 1.56 (d, *J* = 7.2 Hz, 3H). HRMS (ESI) calcd for C_28_H_36_N_3_O_2_S [M + H]^+^, 478.2528; found, 478.2524.

#### (*R*)-5-(2-Aminoethoxy)-2-methyl-*N*-(1-(3-(5-(pyrrolidin-1-ylmethyl)thiophen-2-yl)phenyl)ethyl)benzamide (**48**)

This compound was obtained by a procedure similar to the preparation of **15** (18 mg, yield 64% for 2 steps). ^1^H NMR (400 MHz, methanol-*d*_*4*_, δ): 8.49 (s, 1H), 7.66 (s, 1H), 7.52 (dt, *J* = 7.3, 1.9 Hz, 1H), 7.44–7.29 (m, 3H), 7.15 (dd, *J* = 13.6, 5.9 Hz, 2H), 6.99–6.90 (m, 2H), 5.21 (q, *J* = 7.0 Hz, 1H), 4.33–4.10 (m, 4H), 3.33–3.29 (m, 2H), 3.13–2.94 (m, 4H), 2.25 (s, 3H), 2.00–1.92 (m, 4H), 1.54 (d, *J* = 7.0 Hz, 3H). ^13^C NMR (101 MHz, methanol-*d*_*4*_, δ): 171.84, 157.35, 147.10, 146.39, 139.18, 135.55, 132.91, 131.45, 130.40, 129.46, 126.76, 125.53, 124.74, 124.39, 116.95, 114.30, 65.74, 54.60, 54.13, 50.61, 40.33, 24.09, 22.33, 18.72. HRMS (ESI) calcd for C_27_H_34_N_3_O_2_S [M + H]^+^, 464.2372; found, 464.2366.

#### 2-Methyl-*N*-((*R*)-1-(3-(5-(pyrrolidin-1-ylmethyl)thiophen-2-yl)phenyl)ethyl)-5-(pyrrolidin-3-yloxy)benzamide (**49**)

This compound was obtained by a procedure similar to the preparation of **15** (19 mg, yield 65% for 2 steps). ^1^H NMR (500 MHz, methanol-*d*_*4*_, δ): 8.51 (s, 1H), 7.69 (d, *J* = 1.8 Hz, 1H), 7.55 (dt, *J* = 7.2, 1.7 Hz, 1H), 7.43–7.38 (m, 2H), 7.36 (d, *J* = 3.7 Hz, 1H), 7.24–7.17 (m, 2H), 6.97 (dd, *J* = 8.4, 2.7 Hz, 1H), 6.93 (d, *J* = 2.7 Hz, 1H), 5.23 (q, *J* = 7.1 Hz, 1H), 5.18 (q, *J* = 3.3 Hz, 1H), 4.37 (s, 2H), 3.52 (dt, *J* = 12.8, 1.3 Hz, 1H), 3.49–3.41 (m, 3H), 3.18–3.13 (m, 4H), 2.33–2.22 (m, 5H), 2.05–1.98 (m, 4H), 1.56 (d, *J* = 7.1 Hz, 3H). HRMS (ESI) calcd for C_29_H_36_N_3_O_2_S [M + H]^+^, 490.2528; found, 490.2521.

#### (*R*)-5-Acetamido-*N*-(1-(3-(5-((cyclopentylamino)methyl)thiophen-2-yl)phenyl)ethyl)-2-methylbenzamide (**50**)

This compound was obtained by a procedure similar to the preparation of **15** (20 mg, yield 66% for 2 steps). ^1^H NMR (500 MHz, methanol-*d*_*4*_, δ): 8.55 (s, 1H), 7.69 (d, *J* = 1.8 Hz, 1H), 7.65 (d, *J* = 2.3 Hz, 1H), 7.53 (dt, *J* = 7.2, 1.8 Hz, 1H), 7.43–7.37 (m, 3H), 7.35 (d, *J* = 3.7 Hz, 1H), 7.19–7.15 (m, 2H), 5.22 (q, *J* = 7.0 Hz, 1H), 4.25 (s, 2H), 3.45 (p, *J* = 7.2 Hz, 1H), 2.30 (s, 3H), 2.11 (s, 3H), 2.09–2.01 (m, 2H), 1.84–1.73 (m, 2H), 1.61 (tdd, *J* = 14.6, 6.8, 3.0 Hz, 4H), 1.55 (d, *J* = 7.1 Hz, 3H). HRMS (ESI) calcd for C_28_H_34_N_3_O_2_S [M + H]^+^, 476.2372; found, 476.2366.

#### (*R*)-5-Acetamido-2-methyl-*N*-(1-(3-(5-(pyrrolidin-1-ylmethyl)thiophen-2-yl)phenyl)ethyl)benzamide (**51**)

This compound was obtained by a procedure similar to the preparation of **15** (24 mg, yield 68% for 2 steps). ^1^H NMR (500 MHz, methanol-*d*_*4*_, δ): 7.68 (t, *J* = 1.8 Hz, 1H), 7.60 (d, *J* = 2.3 Hz, 1H), 7.52 (dt, *J* = 7.5, 1.6 Hz, 1H), 7.46 (dd, *J* = 8.2, 2.3 Hz, 1H), 7.40–7.36 (m, 1H), 7.34 (dt, *J* = 7.7, 1.6 Hz, 1H), 7.29 (d, *J* = 3.6 Hz, 1H), 7.20–7.17 (m, 1H), 7.01 (d, *J* = 3.6 Hz, 1H), 5.23 (q, *J* = 7.0 Hz, 1H), 3.94 (s, 2H), 2.72 (d, *J* = 5.9 Hz, 4H), 2.31 (s, 3H), 2.12 (s, 3H), 1.87 (p, *J* = 3.2 Hz, 4H), 1.56 (d, *J* = 7.1 Hz, 3H). ^13^C NMR (126 MHz, methanol-*d*_*4*_, δ): 171.89, 171.70, 146.28, 145.58, 140.72, 138.31, 137.58, 135.95, 132.26, 132.06, 130.28, 129.42, 126.41, 125.31, 124.38, 124.00, 122.41, 119.87, 55.02, 54.63, 50.51, 24.21, 23.73, 22.42, 19.06. HRMS (ESI) calcd for C_27_H_31_N_3_O_2_S [M + H]^+^, 461.2137; found, 462.2209.

#### 5-Acetamido-*N*-((*R*)-1-(3-(5-((((1*S*,3*R*)-3-hydroxycyclopentyl)amino)methyl)thiophen-2-yl)phenyl)ethyl)-2-methylbenzamide (**52**)

This compound was obtained by a procedure similar to the preparation of **15** (22 mg, yield 66% for 2 steps). ^1^H NMR (500 MHz, methanol-*d*_*4*_, δ): 8.55 (s, 1H), 7.69 (s, 1H), 7.65 (d, *J* = 2.2 Hz, 1H), 7.53 (dt, *J* = 7.3, 1.9 Hz, 1H), 7.43–7.36 (m, 3H), 7.35 (d, *J* = 3.7 Hz, 1H), 7.20–7.15 (m, 2H), 5.22 (q, *J* = 7.0 Hz, 1H), 4.31–4.24 (m, 3H), 3.51 (h, *J* = 6.2 Hz, 1H), 2.30 (s, 3H), 2.20 (ddd, *J* = 13.6, 7.8, 5.5 Hz, 1H), 2.11 (s, 3H), 2.09–2.04 (m, 1H), 1.92–1.84 (m, 1H), 1.81 (td, *J* = 7.3, 4.6 Hz, 2H), 1.74 (dt, *J* = 14.0, 4.9 Hz, 1H), 1.55 (d, *J* = 7.1 Hz, 3H). HRMS (ESI) calcd for C_28_H_34_N_3_O_3_S [M + H]^+^, 492.2321; found, 492.2315.

#### (*R*)-5-Amino-*N*-(1-(3-(5-((cyclopentylamino)methyl)thiophen-2-yl)phenyl)ethyl)-2-methylbenzamide (**53**)

This compound was obtained by a procedure similar to the preparation of **15** (25 mg, yield 71% for 2 steps). ^1^H NMR (500 MHz, methanol-*d*_*4*_, δ): 8.55 (s, 1H), 7.68 (t, *J* = 1.7 Hz, 1H), 7.53 (dt, *J* = 7.1, 1.8 Hz, 1H), 7.41–7.35 (m, 2H), 7.34 (d, *J* = 3.7 Hz, 1H), 7.16 (d, *J* = 3.7 Hz, 1H), 6.96 (d, *J* = 7.9 Hz, 1H), 6.74–6.68 (m, 2H), 5.20 (q, *J* = 7.1 Hz, 1H), 4.26 (s, 2H), 3.45 (p, *J* = 7.3 Hz, 1H), 2.20 (s, 3H), 2.13–2.01 (m, 2H), 1.86–1.74 (m, 2H), 1.69–1.56 (m, 4H), 1.55 (d, *J* = 7.1 Hz, 3H). HRMS (ESI) calcd for C_26_H_32_N_3_OS [M + H]^+^, 434.2266; found, 434.2260.

#### (*R*)-5-Amino-2-methyl-*N*-(1-(3-(5-(pyrrolidin-1-ylmethyl)thiophen-2-yl)phenyl)ethyl)benzamide (**54**)

This compound was obtained by a procedure similar to the preparation of **15** (19 mg, yield 65% for 2 steps). ^1^H NMR (500 MHz, DMSO-*d*_6_, δ): 8.55 (d, *J* = 8.2 Hz, 1H), 8.25 (s, 1H), 7.56 (q, *J* = 1.9 Hz, 1H), 7.41 (dt, *J* = 7.7, 1.5 Hz, 1H), 7.31–7.20 (m, 4H), 6.88 (d, *J* = 3.6 Hz, 1H), 6.78 (d, *J* = 8.1 Hz, 1H), 6.48 (d, *J* = 2.4 Hz, 1H), 6.45 (dd, *J* = 8.1, 2.4 Hz, 1H), 5.05 (q, *J* = 7.2 Hz, 1H), 2.02 (s, 3H), 1.64 (t, *J* = 3.6 Hz, 4H), 1.35 (d, *J* = 7.1 Hz, 3H). ^13^C NMR (126 MHz, DMSO-*d*_6_, δ): 168.94, 146.16, 145.95, 143.11, 142.37, 137.78, 133.92, 130.69, 128.94, 126.09, 125.11, 123.26, 122.84, 122.74, 121.22, 114.66, 112.59, 54.10, 53.29, 48.01, 23.18, 22.49, 18.25. HRMS (ESI) calcd for C_25_H_30_N_3_OS [M + H]^+^, 420.2110; found, 420.2104.

#### 5-Amino-*N*-((*R*)-1-(3-(5-((((1*S*,3*R*)-3-hydroxycyclopentyl)amino)methyl)thiophen-2-yl)phenyl)ethyl)-2-methylbenzamide (**55**)

This compound was obtained by a procedure similar to the preparation of **15** (21 mg, yield 67% for 2 steps). ^1^H NMR (500 MHz, methanol-*d*_*4*_, δ): 8.52 (s, 1H), 7.71–7.66 (m, 1H), 7.54 (dt, *J* = 6.8, 2.0 Hz, 1H), 7.43–7.34 (m, 3H), 7.22 (d, *J* = 3.7 Hz, 1H), 6.98 (dd, *J* = 14.3, 7.9 Hz, 1H), 6.76–6.68 (m, 2H), 5.20 (q, *J* = 7.1 Hz, 1H), 4.37 (s, 2H), 4.32 (p, *J* = 4.2 Hz, 1H), 3.60 (p, *J* = 7.3 Hz, 1H), 2.26–2.21 (m, 1H), 2.20 (s, 3H), 2.18–2.10 (m, 1H), 1.97–1.88 (m, 1H), 1.87–1.81 (m, 2H), 1.79 (dt, *J* = 14.1, 4.6 Hz, 1H), 1.55 (dd, *J* = 7.0, 2.1 Hz, 3H). HRMS (ESI) calcd for C_26_H_32_N_3_O_2_S [M + H]^+^, 450.2215; found, 450.2209.

#### (*R*)-5-(Azetidin-3-yloxy)-*N*-(1-(3-(5-((cyclopentylamino)methyl)furan-2-yl)phenyl)ethyl)-2-methylbenzamide (**56**)

This compound was obtained by a procedure similar to the preparation of **15** (24 mg, yield 70% for 2 steps). ^1^H NMR (500 MHz, methanol-*d*_*4*_, δ): 8.55 (s, 1H), 7.78 (t, *J* = 1.8 Hz, 1H), 7.64 (dt, *J* = 7.8, 1.4 Hz, 1H), 7.41 (t, *J* = 7.7 Hz, 1H), 7.37–7.32 (m, 1H), 7.18 (d, *J* = 8.2 Hz, 1H), 6.84–6.76 (m, 3H), 6.59 (d, *J* = 3.4 Hz, 1H), 5.23 (q, *J* = 7.0 Hz, 1H), 5.10 (p, *J* = 5.9 Hz, 1H), 4.38–4.27 (m, 2H), 4.14 (s, 2H), 4.02–3.91 (m, 2H), 3.46–3.37 (m, 1H), 2.26 (s, 3H), 2.11–2.01 (m, 2H), 1.84–1.73 (m, 2H), 1.68–1.53 (m, 4H), 1.56 (d, *J* = 7.1 Hz, 3H). ^13^C NMR (126 MHz, methanol-*d*_*4*_, δ): 171.60, 156.02, 155.52, 149.34, 146.10, 139.33, 133.10, 131.97, 130.18, 129.79, 126.52, 123.69, 122.95, 117.04, 114.70, 113.68, 107.31, 69.90, 60.01, 54.45, 50.66, 44.27, 31.72, 31.68, 25.04, 22.29, 18.69.

#### (*R*)-5-(Azetidin-3-yloxy)-*N*-(1-(3-(5-((cyclopentylamino)methyl)oxazol-2-yl)phenyl)ethyl)-2-methylbenzamide (**57**)

This compound was obtained by a procedure similar to the preparation of **15** (20 mg, yield 66% for 2 steps). ^1^H NMR (500 MHz, methanol-*d*_*4*_, δ): 8.51 (s, 1H), 8.02 (d, *J* = 1.8 Hz, 1H), 7.96 (s, 1H), 7.85 (dt, *J* = 7.6, 1.5 Hz, 1H), 7.57–7.53 (m, 1H), 7.50 (t, *J* = 7.7 Hz, 1H), 7.19 (d, *J* = 8.1 Hz, 1H), 6.86–6.80 (m, 2H), 5.25 (q, *J* = 7.1 Hz, 1H), 5.15 (tt, *J* = 6.5, 4.7 Hz, 1H), 4.51 (dd, *J* = 12.1, 6.7 Hz, 2H), 4.47 (s, 2H), 4.11 (dd, *J* = 12.4, 4.7 Hz, 2H), 3.56 (p, *J* = 7.3 Hz, 1H), 2.27 (s, 3H), 2.18–2.07 (m, 2H), 1.83 (tq, *J* = 10.0, 4.7 Hz, 2H), 1.68 (qd, *J* = 7.3, 3.4 Hz, 4H), 1.58 (d, *J* = 7.1 Hz, 3H). ^13^C NMR (126 MHz, methanol-*d*_*4*_, δ): 171.98, 171.60, 169.95, 155.23, 146.77, 146.45, 139.23, 134.50, 133.20, 131.11, 130.62, 130.16, 129.79, 126.46, 125.27, 117.27, 114.63, 69.13, 60.19, 54.40, 50.54, 42.99, 31.15, 25.02, 22.23, 18.74.

#### (*R*)-5-(Azetidin-3-ylamino)-*N*-(1-(3-(5-((cyclopentylamino)methyl)thiazol-2-yl)phenyl)ethyl)-2-methylbenzamide (**58**)

This compound was obtained by a procedure similar to the preparation of **15** (26 mg, yield 73% for 2 steps). ^1^H NMR (500 MHz, methanol-*d*_*4*_, δ): 8.50 (s, 2H), 8.00 (t, *J* = 1.9 Hz, 1H), 7.92 (s, 1H), 7.83 (dt, *J* = 7.7, 1.5 Hz, 1H), 7.53 (dt, *J* = 7.8, 1.5 Hz, 1H), 7.47 (t, *J* = 7.7 Hz, 1H), 7.01 (d, *J* = 7.9 Hz, 1H), 6.56 (d, *J* = 7.9 Hz, 2H), 5.22 (q, *J* = 7.0 Hz, 1H), 4.48 (p, *J* = 7.0 Hz, 1H), 4.44 (s, 2H), 4.37–4.30 (m, 2H), 3.96–3.88 (m, 2H), 3.53 (p, *J* = 7.4 Hz, 1H), 2.18 (s, 3H), 2.14–2.05 (m, 2H), 1.83–1.75 (m, 2H), 1.69–1.61 (m, 4H), 1.55 (d, *J* = 7.1 Hz, 3H). ^13^C NMR (126 MHz, methanol-*d*_*4*_, δ): 172.65, 171.95, 170.06, 146.93, 146.39, 145.51, 138.76, 134.55, 132.65, 131.30, 130.57, 129.79, 126.37, 125.74, 125.30, 115.67, 112.75, 60.15, 54.83, 50.45, 46.86, 42.99, 31.18, 25.04, 22.28, 18.63. HRMS (ESI) calcd for C_28_H_36_N_5_OS [M + H]^+^, 490.2641; found, 490.2635.

#### (*R*)-5-(Azetidin-3-ylamino)-2-methyl-*N*-(1-(3-(1-methyl-1*H*-pyrrol-2-yl)phenyl)ethyl)benzamide (**59**)

Compound **59** was obtained by using the Suzuki–Miyura cross coupling of **S2** followed by *N*-Boc deprotection in 4 M HCl (in 1,4-dioxane) (22 mg, yield 69% for 2 steps). ^1^H NMR (500 MHz, methanol-*d*_*4*_, δ): 8.54 (s, 1H), 7.44 (t, *J* = 1.8 Hz, 1H), 7.39 (t, *J* = 7.6 Hz, 1H), 7.33 (dt, *J* = 7.8, 1.6 Hz, 1H), 7.30 (dt, *J* = 7.5, 1.6 Hz, 1H), 7.03 (d, *J* = 8.2 Hz, 1H), 6.74 (dd, *J* = 2.7, 1.8 Hz, 1H), 6.57 (dd, *J* = 8.2, 2.6 Hz, 1H), 6.51 (d, *J* = 2.6 Hz, 1H), 6.15 (dd, *J* = 3.5, 1.8 Hz, 1H), 6.10 (dd, *J* = 3.6, 2.7 Hz, 1H), 5.22 (q, *J* = 7.0 Hz, 1H), 4.46 (p, *J* = 7.0 Hz, 1H), 4.34–4.27 (m, 1H), 3.89 (dd, *J* = 10.9, 6.6 Hz, 2H), 3.66 (s, 3H), 2.21 (s, 3H), 1.55 (d, *J* = 7.1 Hz, 3H). HRMS (ESI) calcd for C_24_H_29_N_4_O [M + H]^+^, 389.2341; found, 389.2335.

#### (1*R*,3*S*)-3-(((5-(3-((*R*)-1-(5-(Azetidin-3-ylamino)-2-methylbenzamido)ethyl)phenyl)thiophen-2-yl)methyl)amino)cyclopentyl l-valinate (**60**)

Compound **60** was obtained by using the regular esterification reaction of **S24** with EDC followed by *N*-Boc deprotection in 4 M HCl (in 1,4-dioxane) (16 mg, yield 63% for 2 steps). ^1^H NMR (500 MHz, methanol-*d*_*4*_, δ): 8.46 (s, 1H), 7.69 (d, *J* = 5.9 Hz, 1H), 7.60–7.47 (m, 1H), 7.39 (h, *J* = 5.9 Hz, 3H), 7.28 (s, 1H), 7.03 (dd, *J* = 9.5, 4.9 Hz, 1H), 6.63–6.48 (m, 2H), 5.36–5.13 (m, 2H), 4.59–4.28 (m, 5H), 4.04–3.78 (m, 3H), 3.74–3.59 (m, 1H), 2.62 (dq, *J* = 14.6, 6.9 Hz, 1H), 2.36–2.09 (m, 5H), 2.08–1.88 (m, 4H), 1.55 (t, *J* = 6.6 Hz, 3H), 1.14–0.99 (m, 6H). ^13^C NMR (126 MHz, methanol-*d*_*4*_, δ): 172.58, 170.08, 169.41, 148.00, 146.61, 145.50, 138.81, 135.25, 133.72, 132.66, 130.43, 126.98, 125.69, 125.56, 124.76, 124.67, 115.59, 112.81, 77.92, 59.49, 57.65, 54.83, 50.52, 49.51, 46.82, 45.68, 37.02, 31.94, 31.16, 29.05, 22.39, 18.64, 18.46, 18.35. HRMS (ESI) calcd for C_34_H_46_N_5_O_3_S [M + H]^+^, 604.3321; found, 604.3315.

#### (1*R*,3*S*)-3-(((5-(3-((*R*)-1-(5-(Azetidin-3-ylamino)-2-methylbenzamido)ethyl)phenyl)thiophen-2-yl)methyl)amino)cyclopentyl l-valyl-l-valinate (**61**)

This compound was obtained by a procedure similar to the preparation of **60** (15 mg, yield 61% for 2 steps). ^1^H NMR (500 MHz, methanol-*d*_*4*_, δ): 8.46 (s, 1H), 7.69 (s, 1H), 7.58–7.50 (m, 1H), 7.44–7.34 (m, 3H), 7.28 (d, *J* = 3.8 Hz, 1H), 7.03 (d, *J* = 8.1 Hz, 1H), 6.68–6.51 (m, 2H), 5.22 (t, *J* = 6.9 Hz, 2H), 4.58–4.40 (m, 3H), 4.40–4.29 (m, 3H), 3.94 (s, 2H), 3.80 (t, *J* = 9.4 Hz, 1H), 3.66 (t, *J* = 7.6 Hz, 1H), 2.63 (dd, *J* = 14.1, 7.3 Hz, 1H), 2.21 (d, *J* = 9.4 Hz, 6H), 2.03–1.82 (m, 4H), 1.55 (d, *J* = 7.1 Hz, 3H), 1.10–0.97 (m, 12H). HRMS (ESI) calcd for C_39_H_55_N_6_O_4_S [M + H]^+^, 703.4006; found, 703.3995.

#### 1-Acetoxyethyl 3-((3-(((*R*)-1-(3-(5-(cyclopentanecarboxamidomethyl)thiophen-2-yl)phenyl)ethyl)carbamoyl)-4-methylphenyl)amino)azetidine-1-carboxylate (**62**)

(*R*)-5-(azetidin-3-ylamino)-*N*-(1-(3-(5-(cyclopentanecarboxamidomethyl)thiophen-2-yl)phenyl)ethyl)-2-methylbenzamide (**32**, 26 mg, 0.05 mmol) and 1-(((4-Nitrophenoxy)carbonyl)oxy)ethyl acetate (27 mg, 0.1 mmol) are dissolved in 5 mL of acetonitrile. Diisopropylethylamine (13 μL, 0.075 mmol) is added to the reaction mixture and stirred for overnight at room temperature. The resulting mixture is washed with brine and extracted with Ethyl acetate. The resulting organic layer is concentrated under a reduced pressure and purified by Prep-HPLC afforded **62** (20 mg, yield 61%). ^1^H NMR (500 MHz, methanol-*d*_*4*_, δ): 7.64 (s, 1H), 7.49 (dt, *J* = 7.6, 1.5 Hz, 1H), 7.36 (t, *J* = 7.7 Hz, 1H), 7.31 (d, *J* = 7.7 Hz, 1H), 7.23 (d, *J* = 3.6 Hz, 1H), 7.01 (d, *J* = 8.2 Hz, 1H), 6.94 (d, *J* = 3.6 Hz, 1H), 6.71 (q, *J* = 5.4 Hz, 1H), 6.55 (dd, *J* = 12.3, 4.2 Hz, 2H), 5.20 (q, *J* = 7.0 Hz, 1H), 4.52 (s, 2H), 4.38–4.21 (m, 3H), 3.84–3.72 (m, 2H), 2.70–2.61 (m, 1H), 2.21 (s, 3H), 2.03 (s, 3H), 1.87 (tq, *J* = 6.5, 3.1 Hz, 2H), 1.75 (p, *J* = 8.6 Hz, 4H), 1.64–1.56 (m, 2H), 1.54 (d, *J* = 7.1 Hz, 3H), 1.44 (d, *J* = 5.5 Hz, 3H). HRMS (ESI) calcd for C_35_H_43_N_4_O_6_S [M + H]^+^, 647.2903; found, 647.2897.

#### ((2*R*,3*S*,4*R*,5*R*)-5-(4-Amino-2-oxopyrimidin-1(2*H*)-yl)-3,4-dihydroxytetrahydrofuran-2-yl)methyl (1*R*,3*S*)-3-(((5-(3-((*R*)-1-(5-(azetidin-3-yloxy)-2-methylbenzamido)ethyl)phenyl) Thiophen-2-yl)methyl)amino)cyclopentane-1-carboxylate (**63**)

Compound **63** was obtained by using the regular esterification reaction of **S26** with EDC followed by *N*-Boc deprotection in 4 M HCl (in 1,4-dioxane) (16 mg, yield 63% for 2 steps). ^1^H NMR (500 MHz, DMSO-*d*_6_) 8.85–8.73 (m, 1H), 8.23 (s, 2H), 7.63–7.57 (m, 1H), 7.52–7.47 (m, 1H), 7.39–7.25 (m, 3H), 7.18–7.10 (m, 2H), 6.98 (d, *J* = 3.9 Hz, 1H), 6.85–6.73 (m, 2H), 5.80–5.66 (m, 2H), 5.12 (p, *J* = 7.2 Hz, 1H), 5.06 (s, 1H), 4.27 (dd, *J* = 12.1, 3.0 Hz, 2H), 4.19 (dd, *J* = 12.2, 5.5 Hz, 2H), 4.15–4.03 (m, 3H), 4.01–3.95 (m, 2H), 3.94–3.86 (m, 4H), 3.13 (t, *J* = 7.0 Hz, 1H), 2.80 (dt, *J* = 14.2, 7.9 Hz, 1H), 2.21 (d, *J* = 2.3 Hz, 3H), 2.16–2.06 (m, 1H), 1.91–1.82 (m, 1H), 1.80 (dd, *J* = 14.0, 6.8 Hz, 2H), 1.59 (dt, *J* = 11.9, 8.1 Hz, 1H), 1.44 (dd, *J* = 7.2, 3.1 Hz, 3H). HRMS (ESI) calcd for C_39_H_47_N_6_O_8_S [M + H]^+^, 759.3176; found, 759.3164.

#### ((3*S*,4*R*,5*R*)-3,4-Dihydroxy-5-(4-(hydroxyamino)-2-oxopyrimidin-1(2*H*)-yl)tetrahydrofuran-2-yl)methyl (1*R*,3*S*)-3-(((5-(3-((*R*)-1-(5-(azetidin-3-ylamino)-2-methylbenzamido)ethyl)phenyl)thiophen-2-yl)methyl)amino)cyclopentane-1-carboxylate (**64**)

Compound **64** was obtained by using the regular esterification reaction of **S27** with EDC followed by *N*-Boc deprotection in 4 M HCl (in 1,4-dioxane) (18 mg, yield 66% for 2 steps). ^1^H NMR (400 MHz, DMSO-*d*_6_, δ): 8.70 (dd, *J* = 8.3, 4.1 Hz, 1H), 8.33 (s, 2H), 7.62 (d, *J* = 4.6 Hz, 1H), 7.50 (t, *J* = 7.2 Hz, 1H), 7.39–7.25 (m, 3H), 7.07–6.93 (m, 2H), 6.52–6.34 (m, 3H), 5.75 (dd, *J* = 13.1, 6.3 Hz, 1H), 5.11 (p, *J* = 7.3 Hz, 1H), 4.30 (d, *J* = 12.7 Hz, 1H), 4.13 (d, *J* = 9.3 Hz, 2H), 4.03–3.84 (m, 4H), 3.83–3.74 (m, 1H), 3.72–3.61 (m, 2H), 3.61–3.46 (m, 2H), 3.25–3.04 (m, 1H), 2.12 (s, 3H), 1.87–1.67 (m, 3H), 1.57–1.46 (m, 1H), 1.43 (d, *J* = 7.0 Hz, 3H). HRMS (ESI) calcd for C_39_H_48_N_7_O_8_S [M + H]^+^, 774.3285; found, 774.3279. 13C NMR (126 MHz, methanol-*d*_*4*_, δ): 167.65, 162.89, 160.03, 142.20, 137.21, 136.72, 136.03, 129.44, 125.80, 123.17, 122.66, 122.59, 120.97, 117.42, 116.17, 116.02, 115.30, 115.18, 106.03, 103.36, 89.88, 80.02, 76.65, 65.09, 62.33, 53.35, 49.75, 45.36, 40.98, 37.32, 36.34, 33.71, 25.09, 20.81, 19.02, 12.98, 9.22. HRMS (ESI) calcd for C_39_H_48_N_7_O_8_S [M + H]^+^, 774.3285; found, 774.32796.

### SARS-CoV-2 PLpro Expression and Purification

pET11a vector containing SARS-CoV-2 PLpro protein (pp1ab aa 1564–1878) with *N*-terminal, TEV-cleavable His-tag was transformed into BL21(DE3) cells and maintained in media containing 100 μg/mL carbenicillin. Protein expression was induced using an autoinduction protocol modified from Studier et al.^50^ Briefly, 1 mL day cultures were used to inoculate a 2 L flask of 500 mL of Super LB containing 100 μg/mL carbenicillin. Cells were grown for 24 h at 25 °C and then harvested by centrifugation. All steps of SARS-CoV-2 PLpro purification were performed at 4 °C. Protein yield at each step was monitored by Bradford assay using BSA as a standard. Frozen cells pellets were lysed by sonication in Buffer A (50 mM HEPES, pH 8, 0.5 M NaCl) containing 10 μg/mL lysozyme. The lysate was clarified by centrifugation and loaded onto a 2 mL HiTrap Talon crude column equilibrated with buffer A. Bound His6-PLpro was eluted with a linear gradient of 0–150 mM imidazole in buffer A, and fractions containing His6-PLpro were pooled and exchanged into cleavage buffer (20 mM Tris-HCl pH 8.5, 5 mM DTT, 0.5 mM EDTA, 5% glycerol). A 1:100 molar ratio of TEV protease to PLpro was incubated at 4 °C overnight to cleave the His6-tag. To remove the tag and TEV protease, the reaction was loaded onto a UNO-Q column equilibrated with 20 mM Tris HCl, pH 8.5, 3 mM DTT. Cleaved PLpro eluted first in a gradient from 0 to 150 mM NaCl over 20 column volumes. Fractions containing cleaved PLpro were pooled and concentrated to 12 mg/mL, frozen in liquid nitrogen, and stored at −80 °C.

### PLpro Primary Assay

The PLpro primary assay, which measures protease activity with the short peptide substrate Z-RLRGG-AMC (Bachem), was performed in black, flat-bottom 384-well plates containing a final reaction volume of 50 μL. The assays were assembled at room temperature as follows: 40 μL of 50 nM PLpro in Buffer B (50 mM HEPES, pH 7.5, 0.1 mg/mL BSA, 0.01% Triton-X 100, and 5 mM DTT) was dispensed into wells containing 0.1–1 μL of inhibitor in DMSO or appropriate controls. The enzyme was incubated with inhibitor for 10 min prior to substrate addition. Reactions were initiated with 10 μL of 62.5 μM RLRGG-AMC in buffer B. Plates were shaken vigorously for 30 s, and fluorescence from the release of AMC from peptide was monitored continuously for 15 min on a Tecan Infinite M200 Pro plate reader (λ_excitation_ = 360 nm; λ_emission_ = 460 nm). Slopes from the linear portions of each progress curve were recorded and normalized to plate-based controls. Positive control wells, representing 100% inhibition, included 10 μM GRL0617; negative control wells, representing 0% inhibition, included vehicle alone.

### Cell Culture and Cytotoxicity

The human alveolar epithelial cell line (A549) that stably expresses hACE2 receptor was obtained from BEI Resources (NR-53821). The cells were grown in DMEM supplemented with 10% fetal bovine serum (Gibco), 100 units of penicillin, 100 μg/mL streptomycin (Invitrogen), and 1% nonessential amino acids, with 100 μg/mL blasticidin S. HCl for selection. All cells were grown at 37 °C and 5% CO_2_. Low passage A549 cells (5000 cells/well) were seeded in 96-well plates and incubated at 37 °C and 5% CO_2_ for 24 h prior to a 48 h treatment. All compounds were dissolved in DMSO and final DMSO concentrations never exceeded 1%. The cytotoxicity of compounds (100 μM to 1 μM, 3-fold dilution) was examined using the CellTiter-Glo Luminescent Cell Viability Assay (Promega). Cell cytotoxicity data was normalized to DMSO control as 100% cell viability.

### Virus Production

SARS-CoV-2 strain WA1 (isolate USA-WA1/2020) and strain B.1.617–2 (isolate USA/PHC658/2021) were obtained from BEI Resources and propagated in Vero E6 cells. For the production of viral stocks, cells were infected at an MOI of 0.005 and cultured for 48 h. The cells were harvested with a cell scraper and together with the culture medium spun at 3000 rpm for 10 min. Supernatants were set aside, while the resuspended cell pallets were treated with a Dounce homogenizer and subjected to two freeze–thaw cycles. The homogenates were then recombined with the original supernatants. Following an additional centrifugation step, the supernatants were aliquoted, frozen, and subsequently tittered in serial dilutions by viral plaque assay. All work with SARS-CoV-2 was performed under BSL3 conditions in a facility with negative pressure and PPE that included Tyvek suits and N95 masks for respiratory protection.

### Antiviral Activity Assay

A549-hACE2 cells were seeded at 1.5 × 10^5^ cells/well in DMEM complete into 24-well plates (0.5 mL/well) then incubated for 16 h at 37 °C and 5% CO_2_. Cells were pretreated with compound for 1 h prior to infection performed using a clinical isolate of SARS-CoV-2. Test and control compounds were added to the same volume of SARS-CoV-2 (WA1 final MOI = 0.01; BA.1 final MOI = 0.03) and the mixture was added to the monolayer cells and incubated for 1 h at 37 °C and 5% CO_2_. The mixture was removed and replaced with 0.5 mL of infection media and incubated at 37 °C in 5% CO_2_. After 48 h, supernatants and/or lysates were harvested and processed for RT-qPCR.

### RNA Extraction and RT-qPCR

250 μL of culture fluids were mixed with 750 μL of TRIzol LS Reagent (Thermo Fisher Scientific). RNA was purified following phase separation by chloroform as recommended by the manufacturer. RNA in the aqueous phase was collected and further purified using PureLink RNA Mini Kits (Invitrogen) according to manufacturer’s protocol. Viral RNA was quantified by RT-qPCR using a 7500 Real-Time PCR System (Applied Biosystems) using TaqMan Fast Virus 1-Step Master Mix chemistry (Applied Bio-systems). SARS-CoV-2 N1 gene RNA was amplified using forward (5′-GACCCCAAAATCAGCGAAAT) and reverse (5′-TCTGGTTACTGCCAGTTGAATCTG) primers and probe (5′-FAM-ACCCCGCATTACGTTTGGTGGACC-BHQ1) designed by the United States Centers for Disease Control and Prevention (oligonucleotides produced by IDT, cat# 10006713). RNA copy numbers were determined from a standard curve produced with serial 10-fold dilutions of RNA standard material of the amplicon region from BEI Resources (NR-52358). All data was normalized to virus alone. All error bars represent SD from three replicates.

### Viral Plaque Assay and Other Virus Quantification

Infectious virions were quantified by viral plaque assay. To this end, cells were incubated with SARS-CoV-2 for 2 h and subsequently overlaid with 1% methylcellulose in culture medium. After 3–4 days, the cells were fixed in 10% formalin for 30 min, washed under tap water, and stained with crystal violet. The number of plaques was counted on a light table.

### Animal Studies

All mouse studies with infectious virus were carried out in the ABSL3 facility of the University of Arizona in strict accordance with the recommendations in the Guide for the Care and Use of Laboratory Animals of the National Institutes of Health. The University of Arizona is an AAALAC international accredited animal care organization, and all experiments were approved by the Institutional Animal Care and Use Committee (IUACUC) of the University of Arizona under protocol 14-521. Pharmaron Inc. is accredited with AAALAC. All the procedures related to animal handling, care, and treatment in this study were performed according to guidelines and animal use protocols (ON-CELL-XEN-06012023 and PK-M-07182022) approved by the IACUC of Pharmaron.

### PK and Tolerability Studies in Mice

C57B/6 mice (6–8 weeks) were provided by Vital River Corp. (China) and were used for PK or tolerability studies, which were carried out by Pharmaron Inc., Beijing, China. All animals were group-housed at 3/cage. Access to food and water was provided ad libitum. Animals were monitored for body weight daily, pain or distress or other vital signs throughout the study. All blood collection was done using series bleeding via dorsal metatarsal veins using an EDTA-K2 tube at predetermined time points and stored on ice until centrifugation to obtain plasma, which was stored frozen at −20 °C or lower. At the completion of the study, animals were euthanized by overdose of inhaled anesthesia followed by exsanguination. In PK studies, male mice were used. **10** (free base) was used for PK studies by PO, IV or SC injection. Vehicle for PO was 10%PEG-400 in 90% (20%HP-beta-cyclodextran in water). In the IV and SC studies, vehicle was 20%HP-beta-cyclodextran in water. In the tolerability study, **10** (HCl) salt was used. Female mice were dosed by either IV via tail-vein injection (at 5 or 10 mg/kg) at one dose, or by subcutaneous injection at 100 mg/kg QD for 2 days. Vehicle for IV injection was 3%Solutol, 5%DMSO, 20%PEG-400, 72% saline. Saline was used as vehicle for SC dosing. Plasma and lungs were collected at 1 min post dosing (10 mg/kg IV) or 5 min post dosing (5 mg/kg IV), or 30 min post dosing (100 mg/kg, SC). Lungs were collected and immediately frozen in liquid nitrogen. Plasma and lung homogenates were extracted with acetonitrile, analyzed and quantified by LC–MS/MS (see Supporting Information Section). A noncompartment model was used to obtain PK parameters in WinNonlin 8.3.

### Mice, Drug Treatment, and SARS-CoV-2 Infection In Vivo

C57BL/6 mice were bred and kept under SPF conditions in the animal facility of the University of Arizona. At an age of 8–12 weeks, mice of both sexes were injected i.p. twice daily for the duration of the experiment with the selected compounds at the indicated concentrations, starting 1 day prior to infection. The mice were then infected intranasally with 5 × 10^4^ Pfu of mouse-adapted SARS-CoV-2 MA10 (BEI Bioscience) 2 h after receiving the last compound injection. Vehicle-treated or mock-infected animals served as controls.^51^ Viral titers of infectious virions were measured 2 days post infected by serial dilutions of lung homogenates in viral plaque assays.

### Statistical Analysis

GraphPad Prism 8 software package (GraphPad Software, USA) was used to perform statistical analysis. All data were presented as the mean ± SD unless otherwise noted. One-way analysis of variance with appropriate posthoc tests (3+ groups) and Student’s *t*-test (2 groups) were used to calculate statistical significance: *P < 0.05, **P < 0.01, ***P < 0.001.

## Supporting Information Available

synthesis and compound characterization; and further experimental details of biophysical, cytotoxic, pharmacokinetic properties and HPLC of representative compounds (PDF)
PLpro SMILES (CSV)

## Abbreviations

3CLpro: 3C-like protease
ACE2: angiotensin-converting enzyme 2
ADME: absorption, distribution, metabolism and excretion
ANOVA: one-way analysis of variance
Arg: arginine
Asp: aspartic acid
Boc: tertbutyloxycarbonyl
BSA: bovine serum albumin
CYP: cytochrome P450
COVID-19: coronavirus disease 2019
DMF: dimethylformamide
DCM: dichloromethane
DMAP: 4-dimethylaminopyridine
DTT: dithiothreitol
DUB: deubiquitinase
DMEM: Dulbecco’s Modified Eagle Medium
EDTA: ethylenediamine tetraacetic acid
EUA: emergency use authorization
FDA: Food and Drug Administration
Gln: glutamine
Glu: glutamic acid
HIV: human immunodeficiency virus
HOAc: acetic acid
HATU: hexafluorophosphate azabenzotriazole tetramethyl uronium
HATU: hexafluorophosphate azabenzotriazole tetramethyl uronium
HEPES: 4-(2-hydroxyethyl)-1-piperazineethanesulfonic acid
i.v.: intravenous
i.p.: intraperitoneal
ISG15: interferon-stimulated gene 15
LCMS: liquid chromatography–mass spectrometry
MERS-CoV: middle East respiratory syndrome coronavirus
Mpro: SARS coronavirus main proteinase
NADPH: nicotinamide adenine dinucleotide phosphate hydrogen
PLpro: papain-like protease
PCC: post-COVID-19 condition
PDB: protien data bank
PEG-400: polyethylene glycol 400
p.o.: oral
Pro248: proline 248
RdRp: RNA-dependent RNA polymerase
RT-PCR: reverse transcription polymerase chain reaction
RBD: receptor-binding domain
RTqPCR: reverse transcription quantitative polymerase chain reaction
SAR: structure activity relationship
s.c.: subcutaneous
SARS-CoV-2: severe acute respiratory syndrome coronavirus 2
SD: standard deviation
SPR: surface plasmon resonance
TEV: tobacco etch virus
TLC: thin-layer chromatography
TMPRSS2: transmembrane protease serine 2
Tyr: tyrosine
Ub: ubiquitin
UbL: ubiquitin-like proteins
VOC: variants of concern
XPhos: 2-dicyclohexylphosphino-2′,4′,6′-triisopropylbiphenyl
