# Supplementary material for: Non-Covalent Inhibitors of SARS-CoV-2 Papain-Like Protease (PLpro): In Vitro and In Vivo Antiviral Activity
Source: J Med Chem. 2024 Aug 5;67(16):13681–702. doi: 10.1021/acs.jmedchem.4c00378 (PMC11345844; doi:10.1021/acs.jmedchem.4c00378)
Supplement: Supplementary file 1 — jm4c00378_si_001.pdf [file jm4c00378_si_001.pdf]

SUPPORTING INFORMATION

Non-Covalent Inhibitors of SARS-CoV-2 Papain-Like Protease (PLpro): In Vitro and In Vivo Antiviral Activity

Ganga Reddy Velma[1≠], Zhengnan Shen[1≠], Cameron Holberg[1], Jiqiang Fu[1], Farinaz Soleymani[2], Laura Cooper[3], Omar Lozano Ramos[1], Divakar Indukuri[1], Soumya Reddy Musku[1], Pavel Rychetsky[1], Steve Slilaty[4], Zuomei Li[4], Kiira Ratia[5], Lijun Rong[3], Dominik Schenten[6], Rui Xiong[1]*, Gregory R J Thatcher[1,2]*

[1]Department of Pharmacology & Toxicology, R. Ken Coit College of Pharmacy, University of Arizona, Tucson, AZ 85721, USA; [2]Department of Chemistry & Biochemistry, Colleges of Science and Medicine, University of Arizona, Tucson, AZ 85721, USA; [3]Department of Microbiology, College of Medicine, University of Illinois at Chicago (UIC), Chicago, IL 60612, USA; [4]Sunshine Biopharma Inc., 333 Las Olas Way, CU4 Suite 433, Fort Lauderdale, Florida 33301, USA; [5]Research Resources Center, University of Illinois at Chicago (UIC), Chicago, IL 60612, USA; [6]Department of Immunology, College of Medicine, University of Arizona, Tucson, AZ 85721, USA

*Corresponding authors: Gregory Thatcher, grjthatcher@arizona.edu

Rui Xiong, xiongr@arizona.edu

Keywords: SARS, cysteine protease, non-covalent inhibitor, PLpro, antiviral agent

Table of Contents

1. Figures (PK data).. S1-S5: pp 1-2
2. Tables (Pharmacokinetic profiles)……………………………………..…………T1-T6: pp 3-4
3. General Chemical Experimental Procedures……………………………………….pp 4-5
4. NMR Spectra..pp 6-39
5. HPLC Traces...pp 40-46

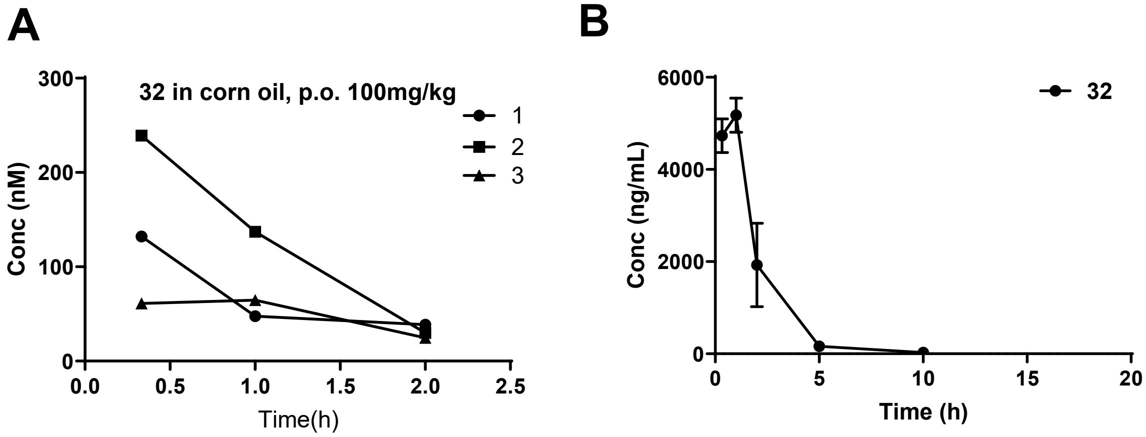

Figure S1. Plasma PK of **32**: **A** by gavage (3 individual mice shown) and **B** (25 mg/kg i.p.).

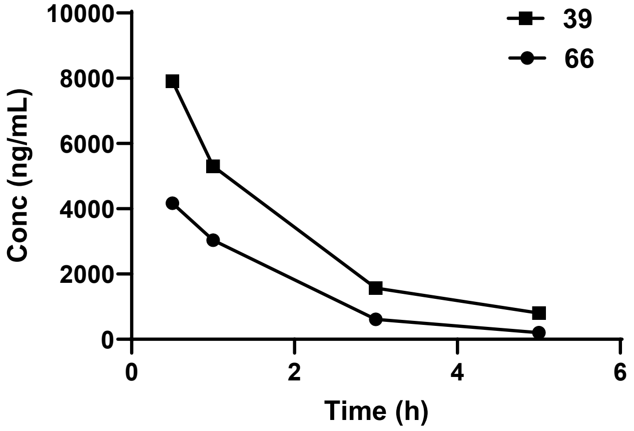

Figure S2. Bioactivation products **39** and **66** were detected in plasma PK of **64** (25 mg/kg i.p.), whereas **64** was not detectable.

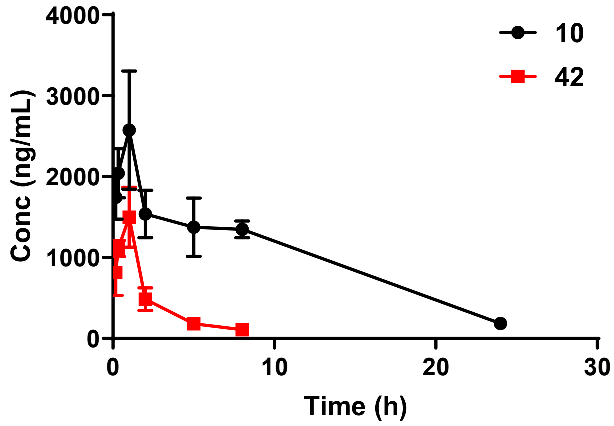

Figure S3. Comparison of plasma PK of **10** and oxo-analogue **42** (25 mg/kg i.p.).

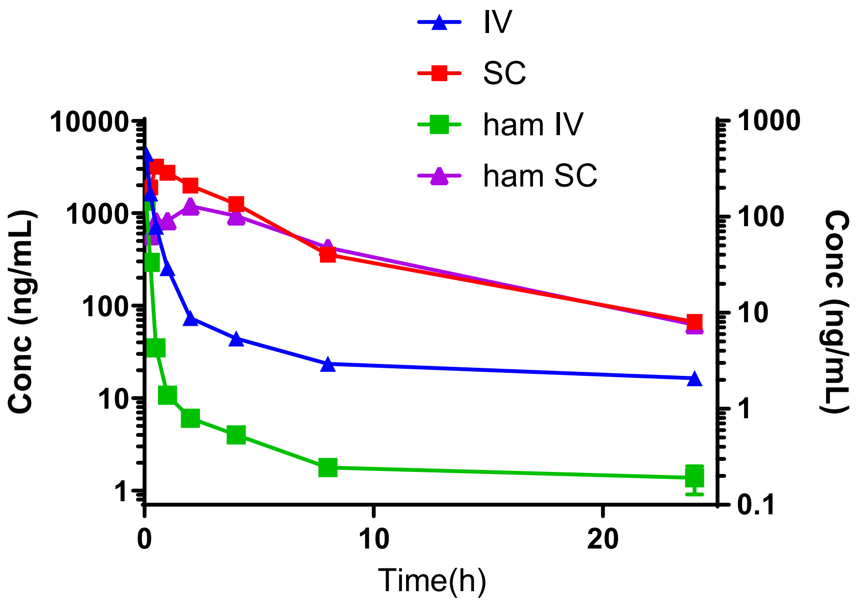

Figure S4. Plasma concentration vs time profile of **10** after 2 mg/kg, IV in C57BL/67 mouse and hamster; 50 mg/kg, SC in C57BL/67 mouse; and 20 mg/kg, SC in hamster

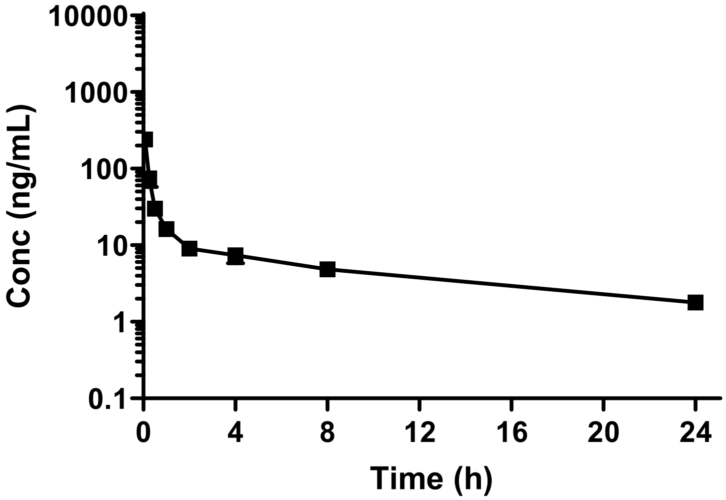

Figure S5. Plasma concentration vs time profile of **42** after 2 mg/kg, IV in hamster

**Table S1.** Plasma and lung concentration after final injection of **10** (HCl salt) administered at 5 mg/kg (i.v. bid) for 0.5 days							
	**Time**	**Concentration (ng/mL)**			**Mean**	**SD**	**CV**
	(h)	Mouse 16	Mouse 24	Mouse 36	(ng/mL)	(ng/mL)	(%)
Plasma	0.083	1920	1800	2010	1910	105	5.52
Lung	0.083	9650	10700	10300	10217	530	5.19

**Table S2.** Lung/Plasma ratio profile from Table S1						
**Time**	**Lung/Plasma ratio**			**Mean**	**SD**	**CV**
(h)	Mouse 16	Mouse 24	Mouse 36			(%)
0.083	5.03	5.94	5.12	5.36	0.50	9.40

**Table S3.** Plasma and lung concentration after final injection of **10** (HCl salt) administered at 10 mg/kg (i.v. qd) for 2 days							
	**Time**	**Concentration (ng/mL)**			**Mean**	**SD**	**CV**
	(h)	Mouse 28	Mouse 32		(ng/mL)	(ng/mL)	(%)
Plasma	0.0167	12300	9830		11065	NA	NA
Lung	0.0167	39250	32800		36025	NA	NA

**Table S4.** Lung/Plasma ratio profile from Table S3						
**Time**	**Lung/Plasma ratio**			**Mean**	**SD**	**CV**
(h)	Mouse 16	Mouse 24	Mouse 36			(%)
0.0167	3.19	3.34		3.26	NA	NA

**Table S5.** Plasma and lung concentration after injection of **10** (HCl salt) administered at 10 mg/kg (s.c. qd) for 1 days							
	**Time**	**Concentration (ng/mL)**			**Mean**	**SD**	**CV**
	(h)	Mouse 4	Mouse 8	Mouse 30	(ng/mL)	(ng/mL)	(%)
Plasma	0.5	6170	7180	7730	7027	791	11.3
Lung	0.5	89500	92000	88500	90000	1803	2.00

**Table S6.** Lung/Plasma ratio profile from Table S5 data						
**Time**	**Lung/Plasma ratio**			**Mean**	**SD**	**CV**
(h)	Mouse 4	Mouse 8	Mouse 30			(%)
0.5	14.5	12.8	11.4	12.9	1.5	11.8

Chemical Experimental Procedures

Majority of the synthons were prepared and characterized similarly as reported in our privously published work[1] and Compound **S26** was prepared using a method reported in the literature.[2]

General Procedure for Reductive Amination. To a solution of amine compound and ketone (or aldehyde) compound in MeOH, HOAc was added. After stirring at the indicated temperature for 2 h and cooldown, $NaBH_3CN$ was added carefully. The reaction was continued at room temperature overnight and then concentrated under vacuum. Dissolve the mixture in EA and wash with water and brine. After that, the organic layer was dried over Na_2SO_4, filtered, and concentrated. The residue was purified by silica gel column chromatography or Prep-HPLC to provide the amination compound.

General Procedure for Amine Coupling/Esterification. Amine compound/alcohol, acid compound, HATU or EDC, TEA (or DIPEA) and DMAP were dissolved in dry DMF or DCM and stirred at room temperature overnight. The mixture was diluted with ethyl acetate and was then washed with saturated aq. $NaHCO_3$, water, and brine, respectively. The organic layer was dried over Na_2SO_4, filtered, and concentrated. The residue was purified by silica gel column chromatography or Prep-HPLC to provide the desired amide/ester.

General Procedure for N-Boc Deprotection. To a solution of Boc protected compound in DCM was added HCl (4M in dioxane) at 0 °C, and then warmed up to room temperature. After stirring for another 2 h, the reaction was dried under vacuum. The residue was purified by Prep-HPLC to provide the deprotected compound.

References:

1. Shen, Z.; Ratia, K.; Cooper, L.; Kong, D.; Lee, H.; Kwon, Y.; Li, Y.; Alqarni, S.; Huang, F.; Dubrovskyi, O.; Rong, L.; Thatcher, G. R. J.; Xiong, R. Design of SARS-CoV-2 PLpro

Inhibitors for COVID-19 Antiviral Therapy Leveraging Binding Cooperativity. *J Med Chem* **2022,** 65, 2940-2955.

2. Paymode, D. J.; Vasudevan, N.; Ahmad, S.; Kadam, A. L.; Cardoso, F. S. P.; Burns, J. M.; Cook, D. W.; Stringham, R. W.; Snead, D. R. Toward a practical, two-step process for Molnupiravir: direct hydroxamination of cytidine followed by selective esterification. *Org. Process Res. Dev.* **2021**, *25*, 1822– 1830.

NMR Spectra

Compound **15**

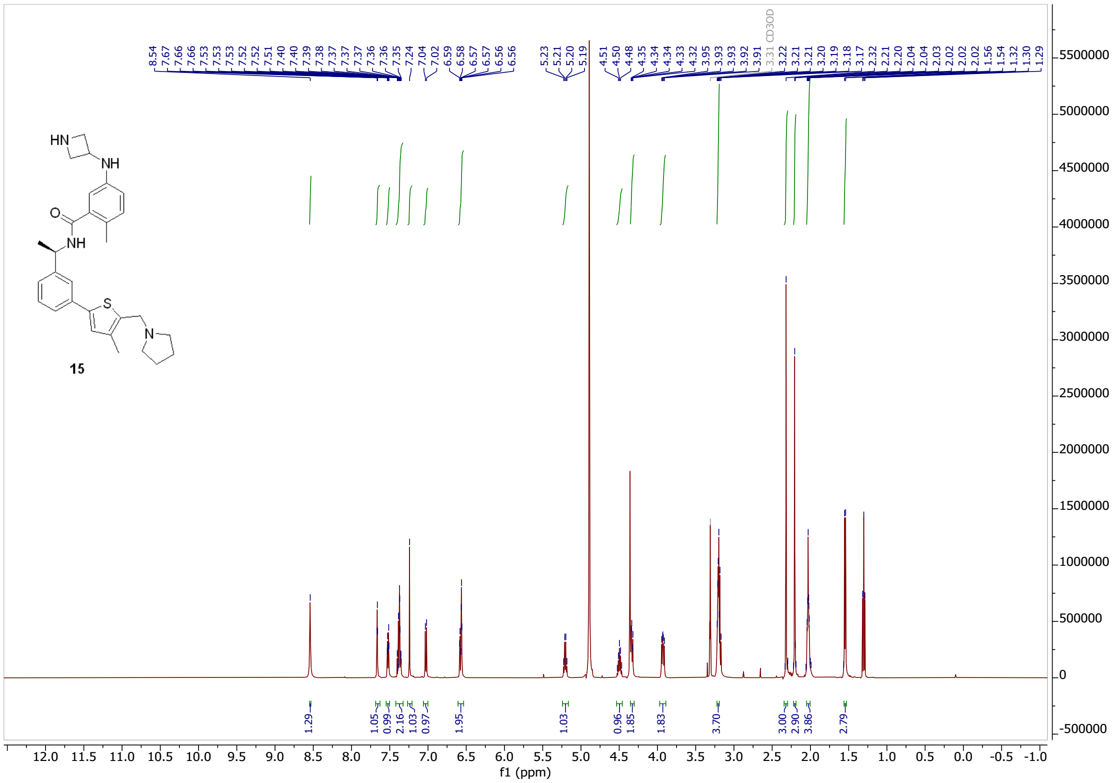

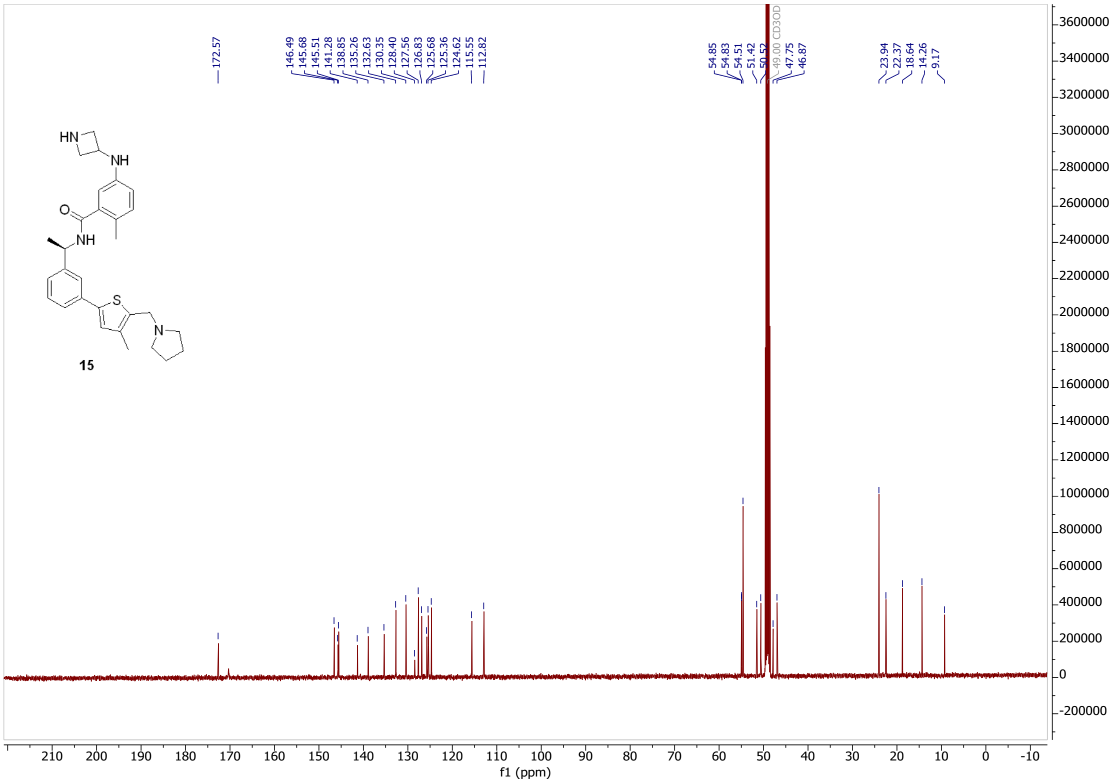

Compound **16**

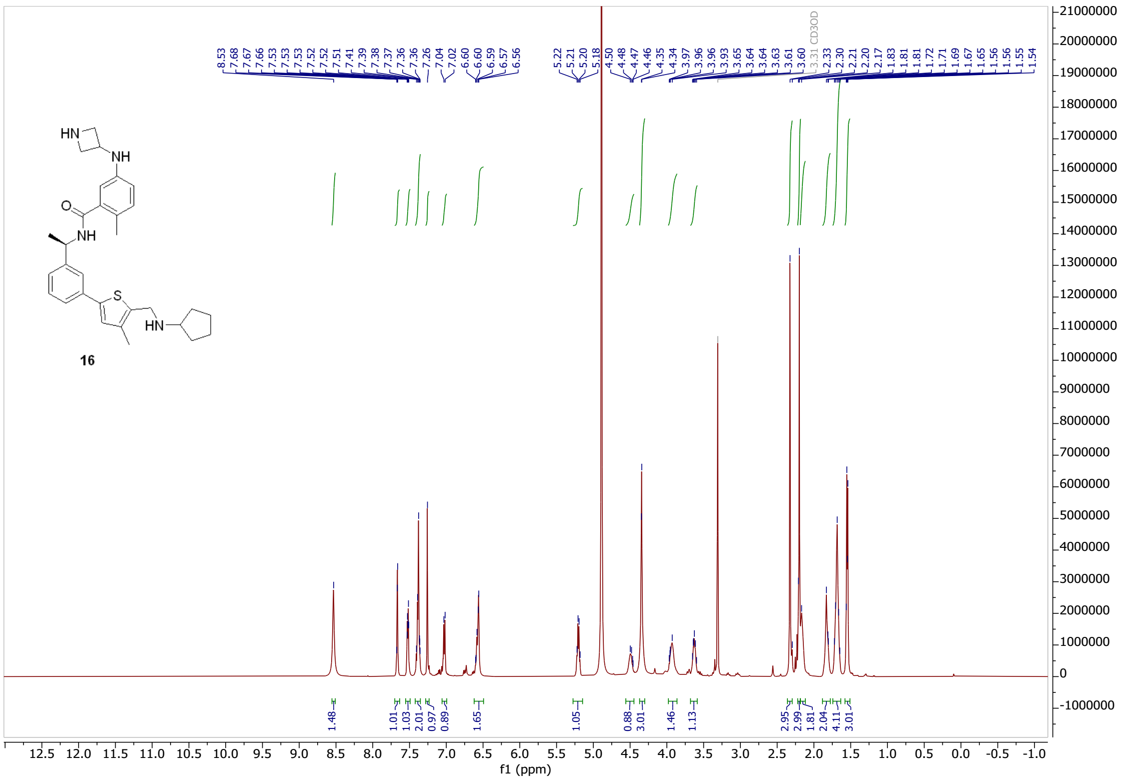

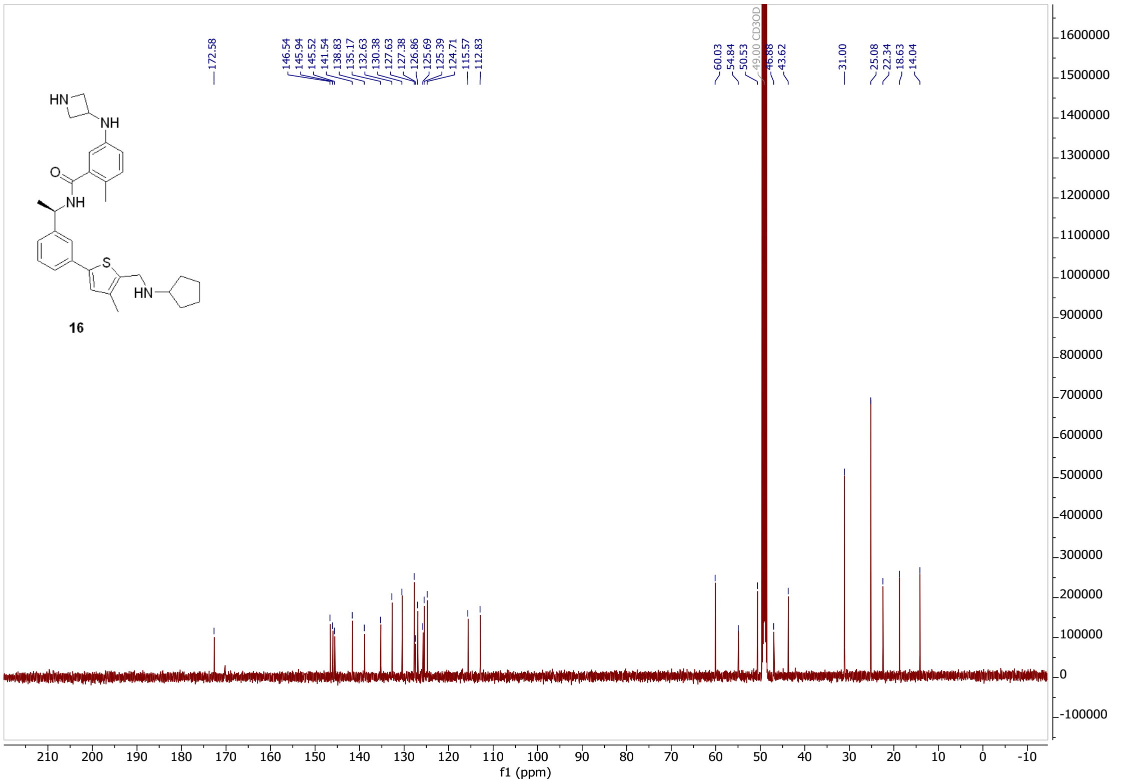

Compound **17**

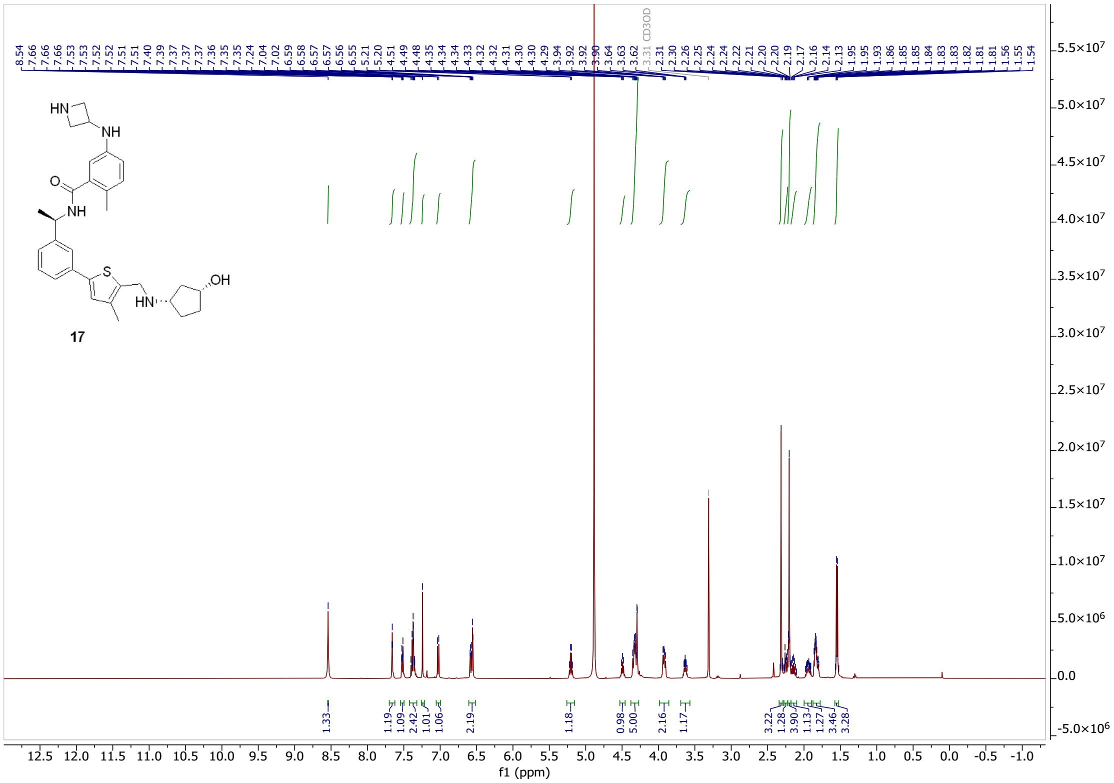

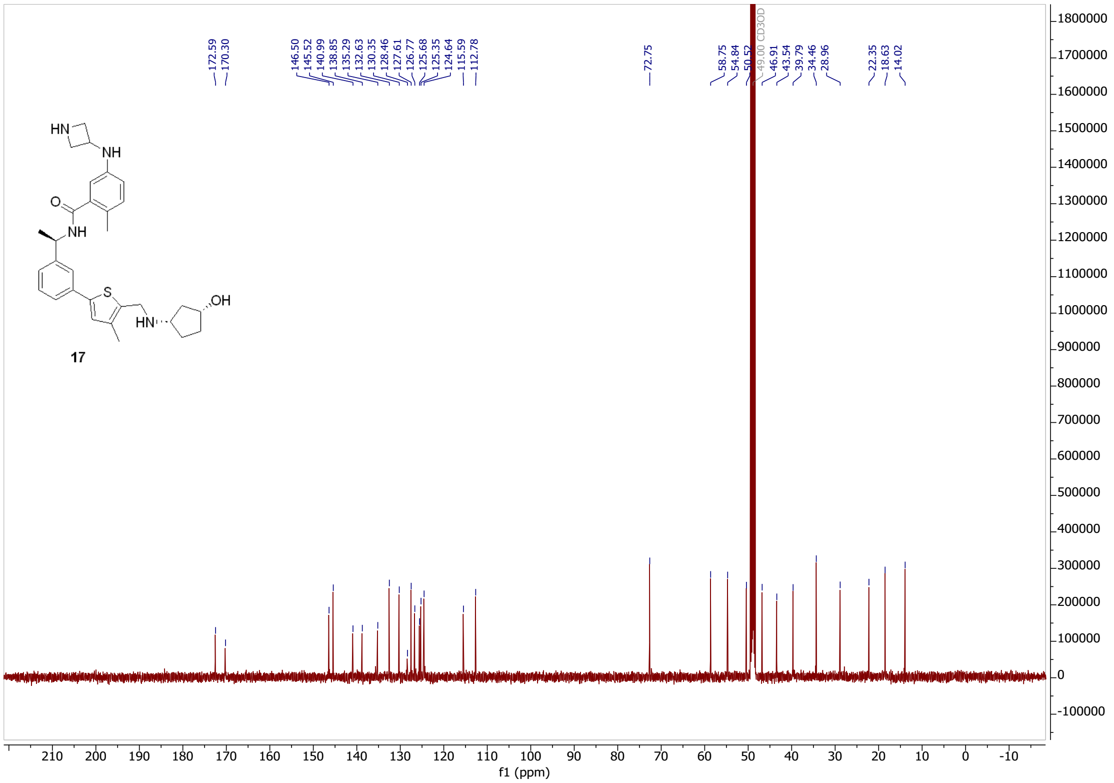

Compound **18**

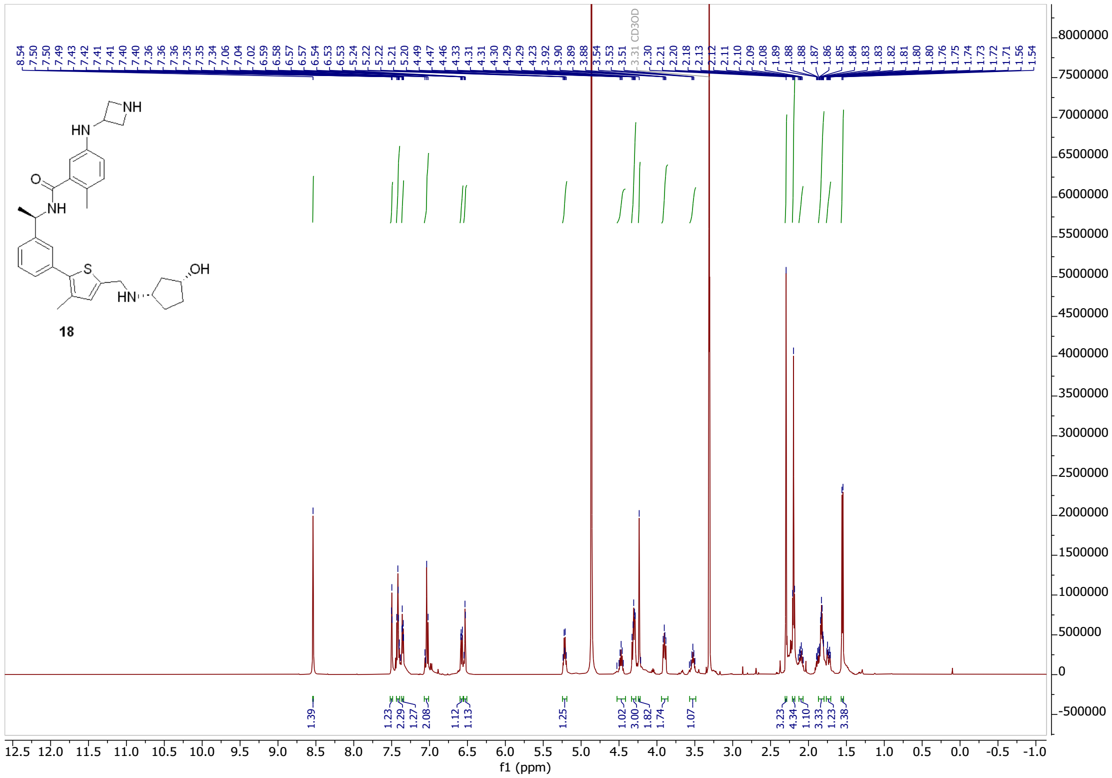

Compound **19**

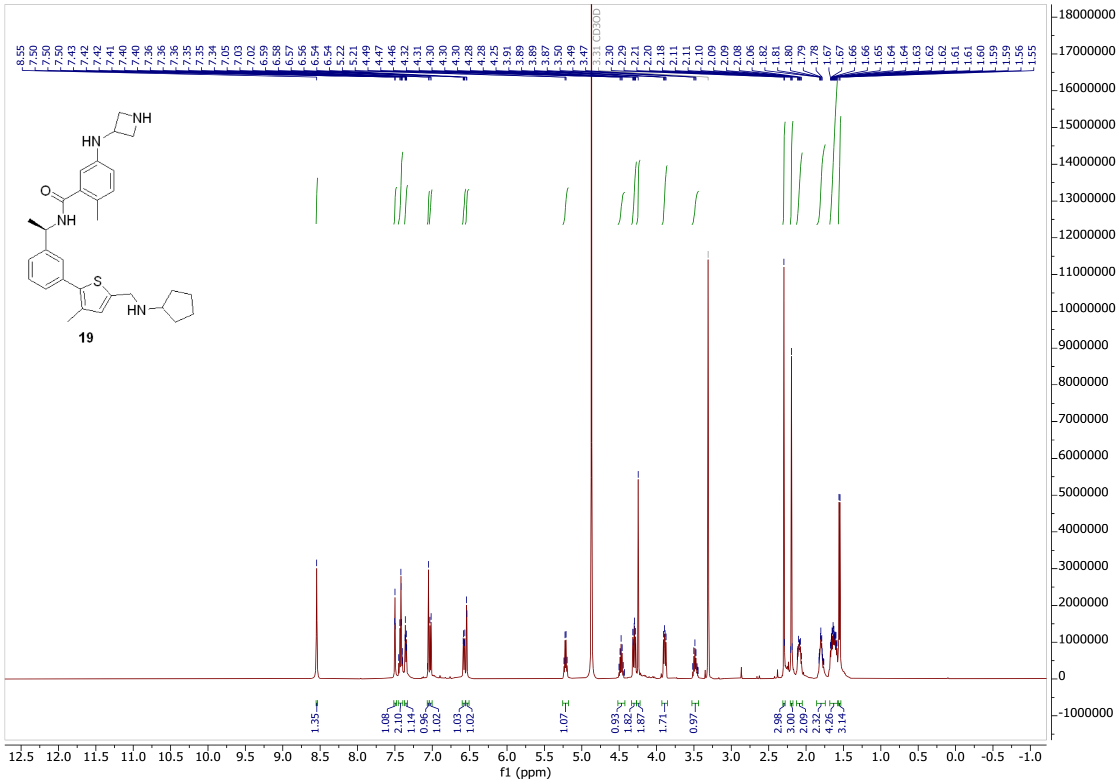

Compound **20**

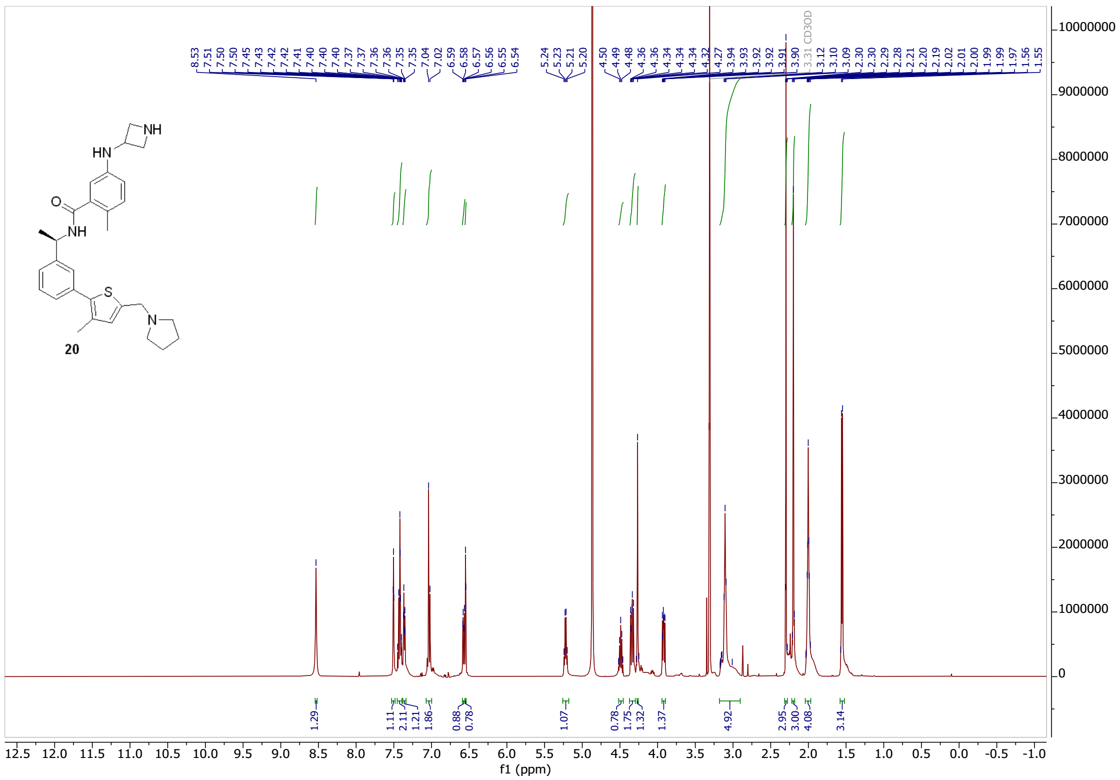

Compound **21**

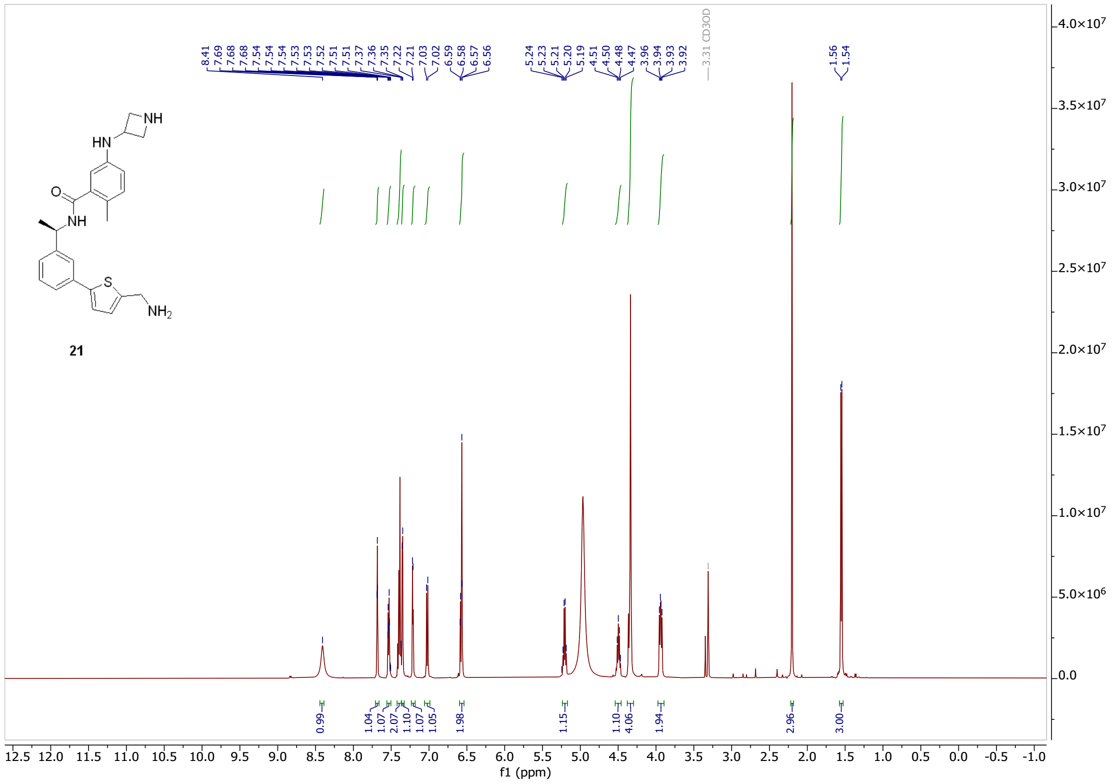

Compound **22**

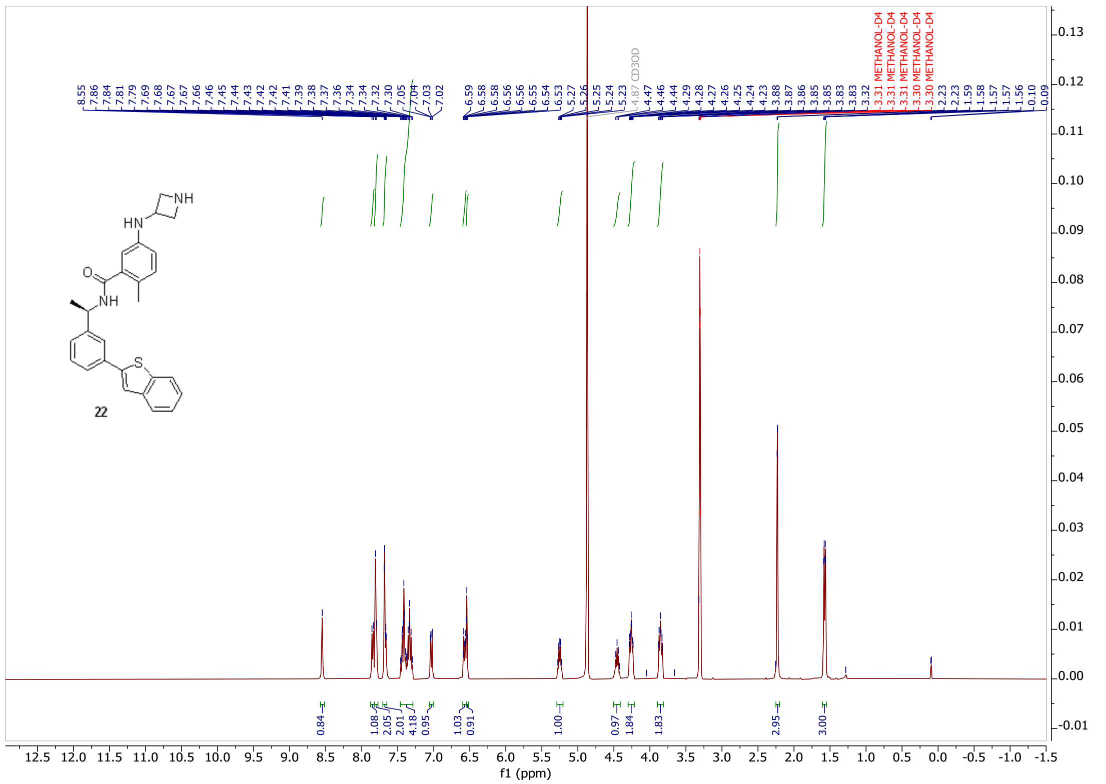

8.55 7.86 7.84 7.81 7.79 7.69 7.68 7.67 7.67 7.66 7.46 7.45 7.44 7.43 7.42 7.42 7.41 7.39 7.38 7.37 7.36 7.34 7.34 7.32 7.30 7.05 7.04 7.03 7.02 6.59 6.58 6.58 6.56 6.56 6.55 6.54 6.53 5.27 5.26 5.25 5.24 5.23
4.87 CD3OD
4.47 4.46 4.44 4.29 4.28 4.27 4.26 4.25 4.24 4.23 3.88 3.87 3.86 3.85 3.85 3.83 3.83 3.32
3.31 METHANOL-D4
3.31 METHANOL-D4
3.31 METHANOL-D4
3.30 METHANOL-D4
3.30 METHANOL-D4
2.23 2.23 1.59 1.58 1.57 1.57 1.56 0.10 0.09
NH
HN
O
NH
S
22
0.84 1.08 2.05 2.01 4.18 0.95 1.03 0.91 1.00 0.97 1.84 1.83 2.95 3.00
f1 (ppm)

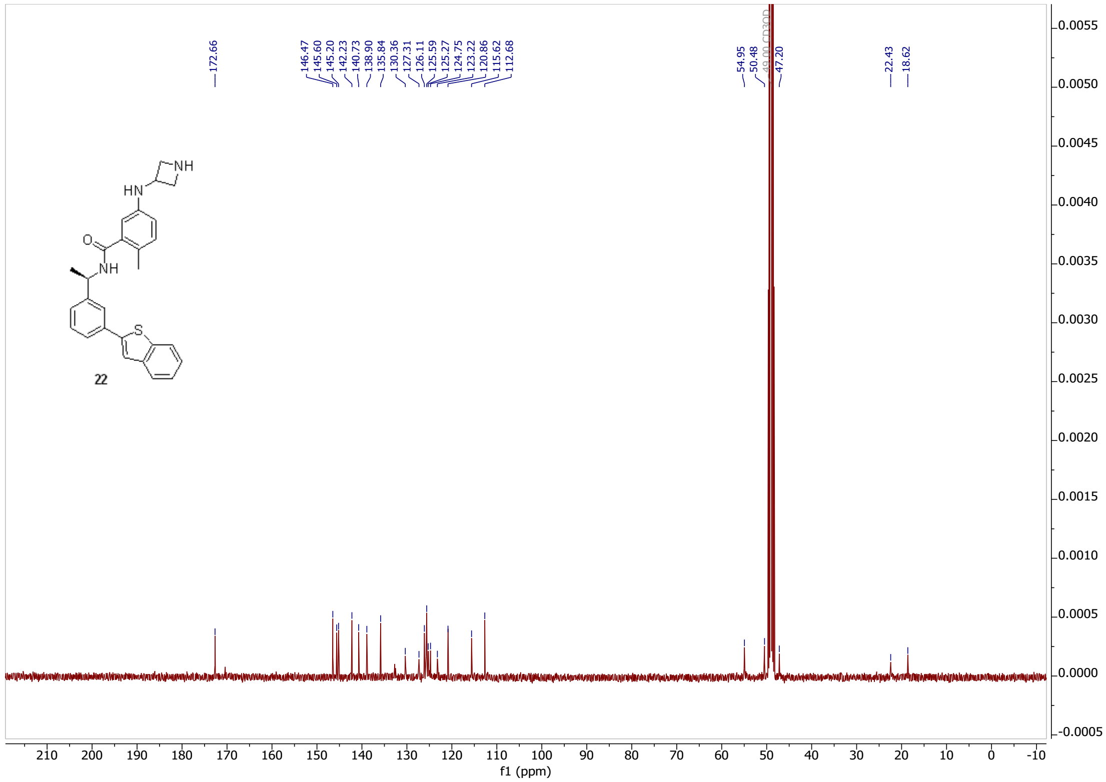

172.66
146.47 145.60 145.20 142.23 140.73 138.90 135.84 130.36 127.31 126.11 125.59 125.27 124.75 123.22 120.86 115.62 112.68
54.95 50.48 49.00 CD3OD 47.20
22.43 18.62
NH
HN
O
NH
S
22
f1 (ppm)

Compound **23**

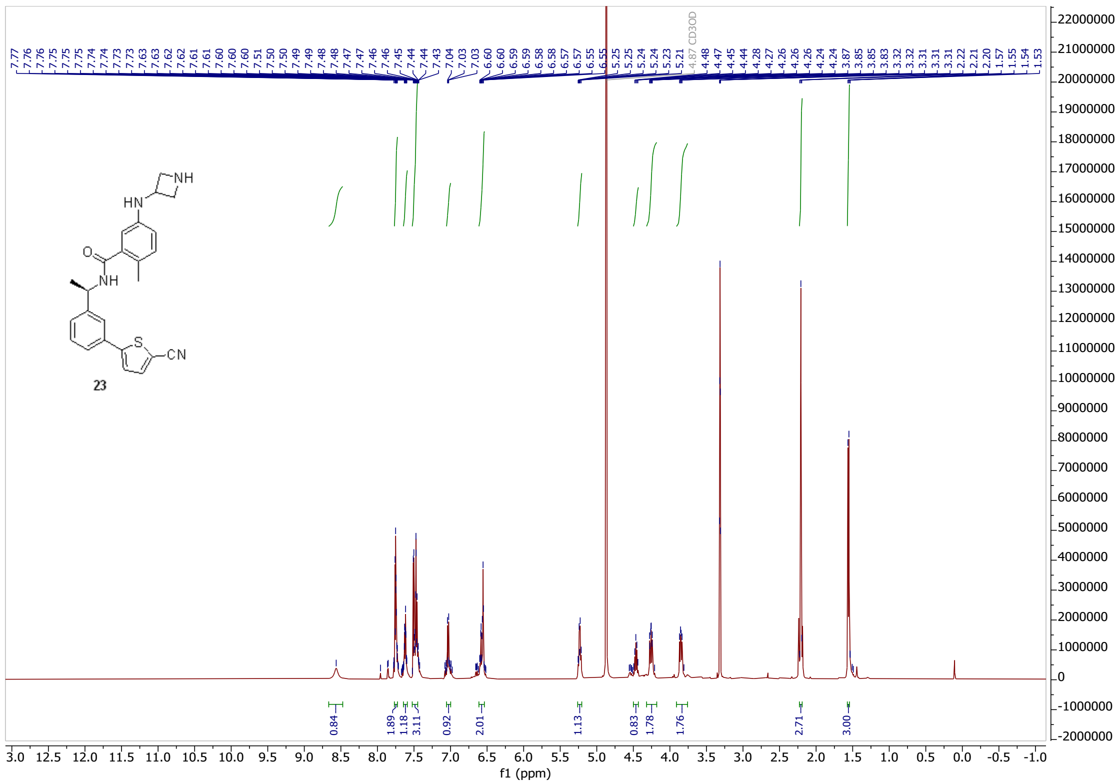

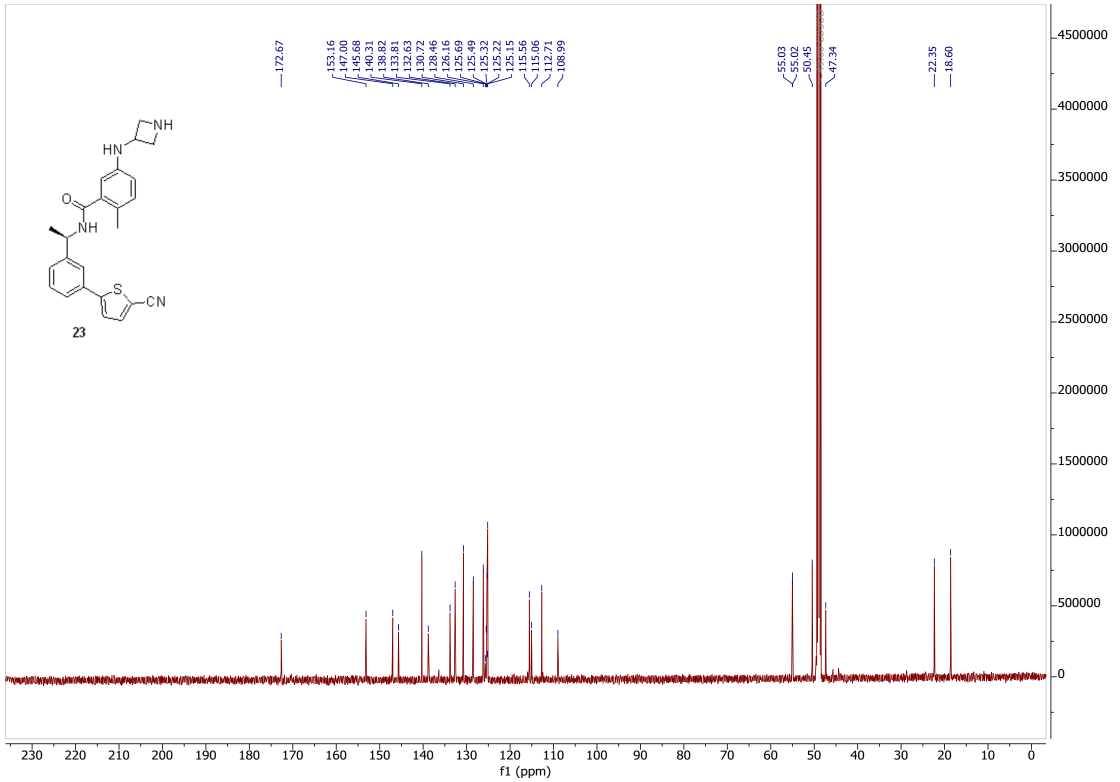

Compound **24**

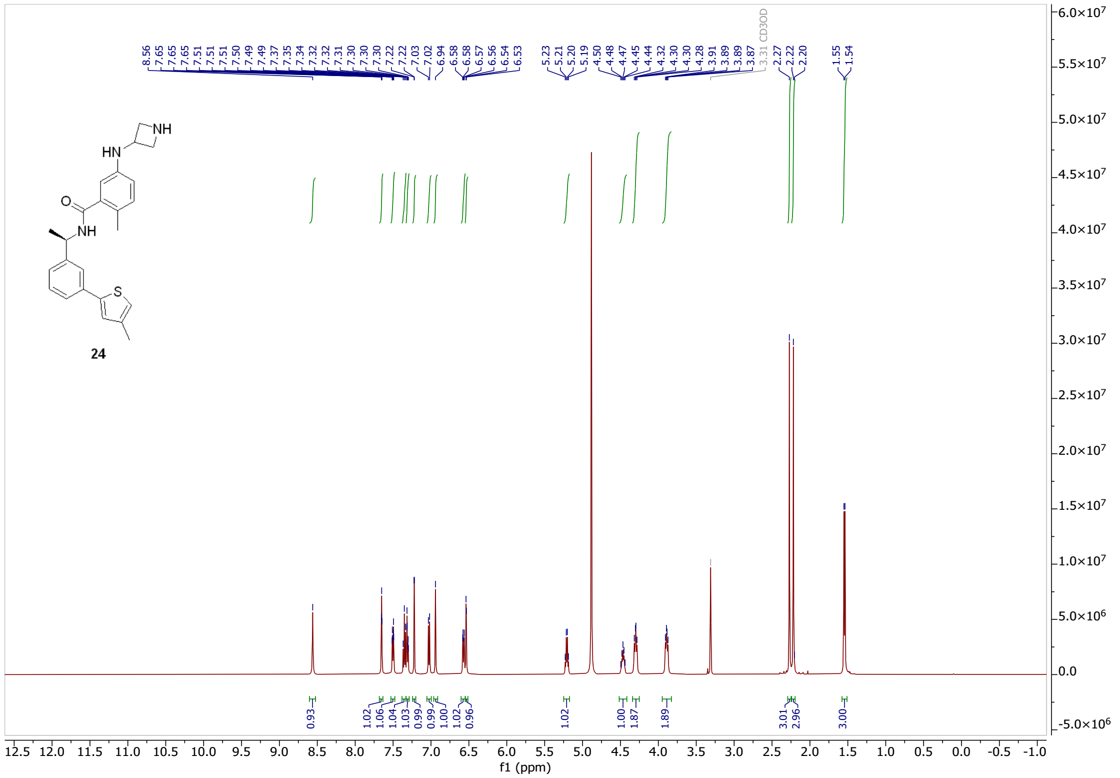

Compound **25**

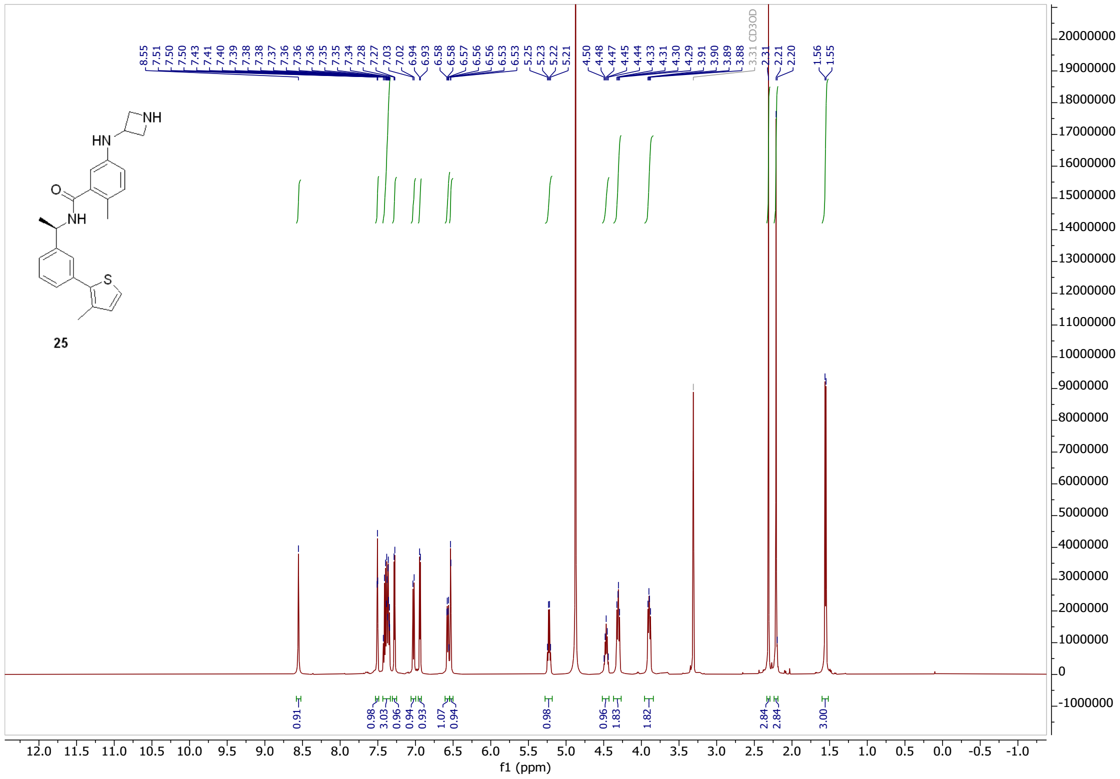

Compound **26**

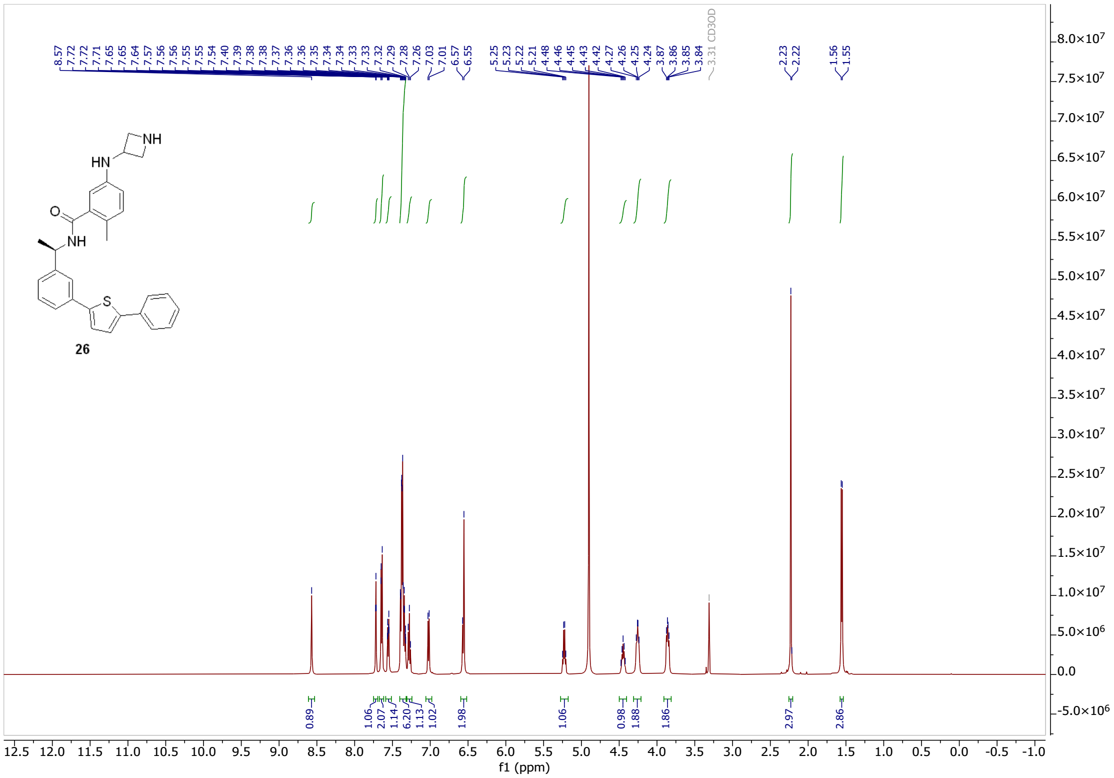

Compound **27**

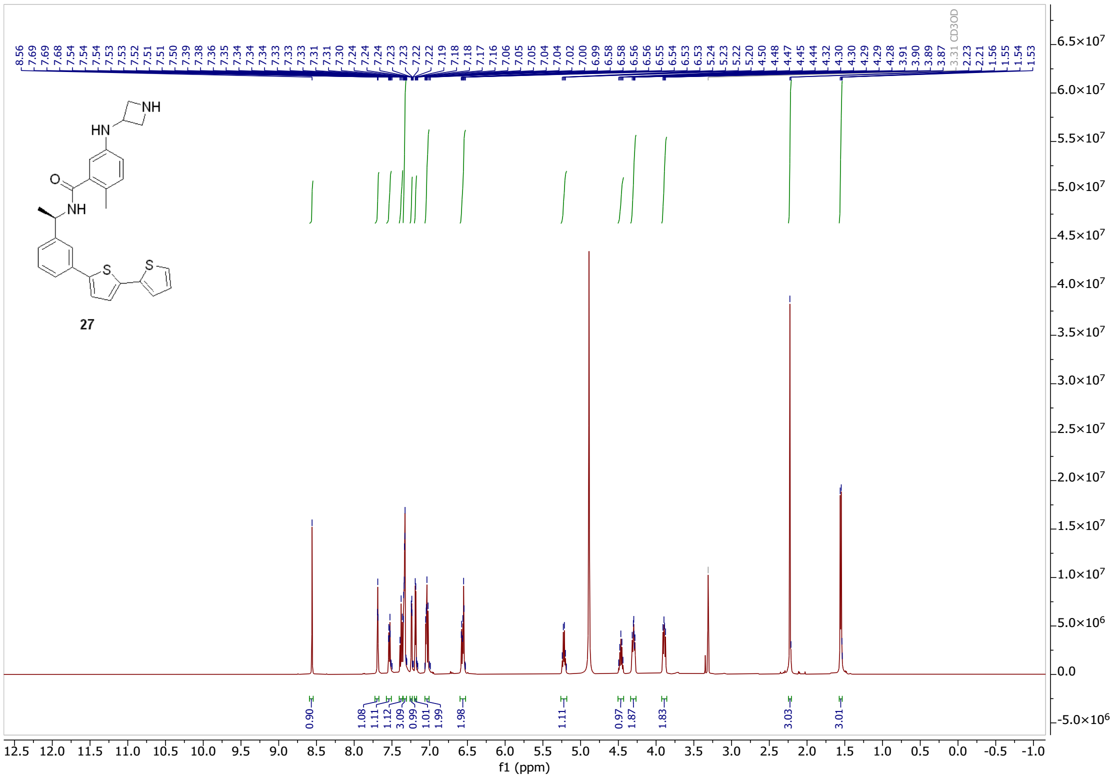

Compound **28**

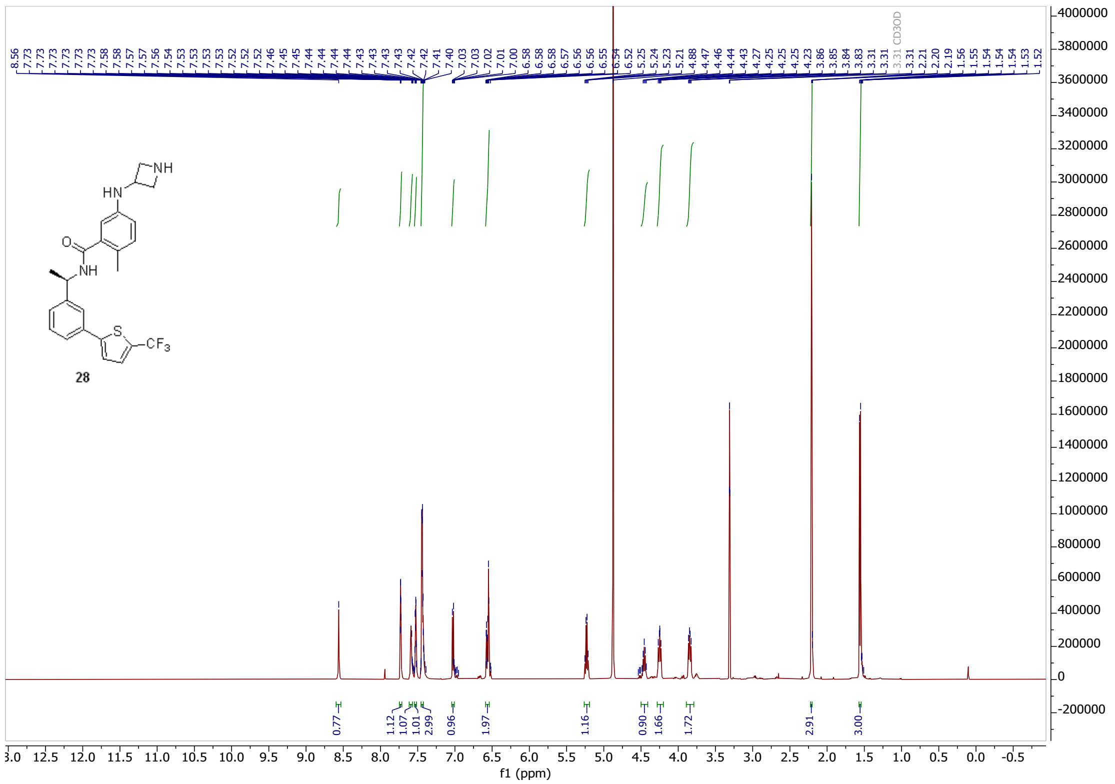

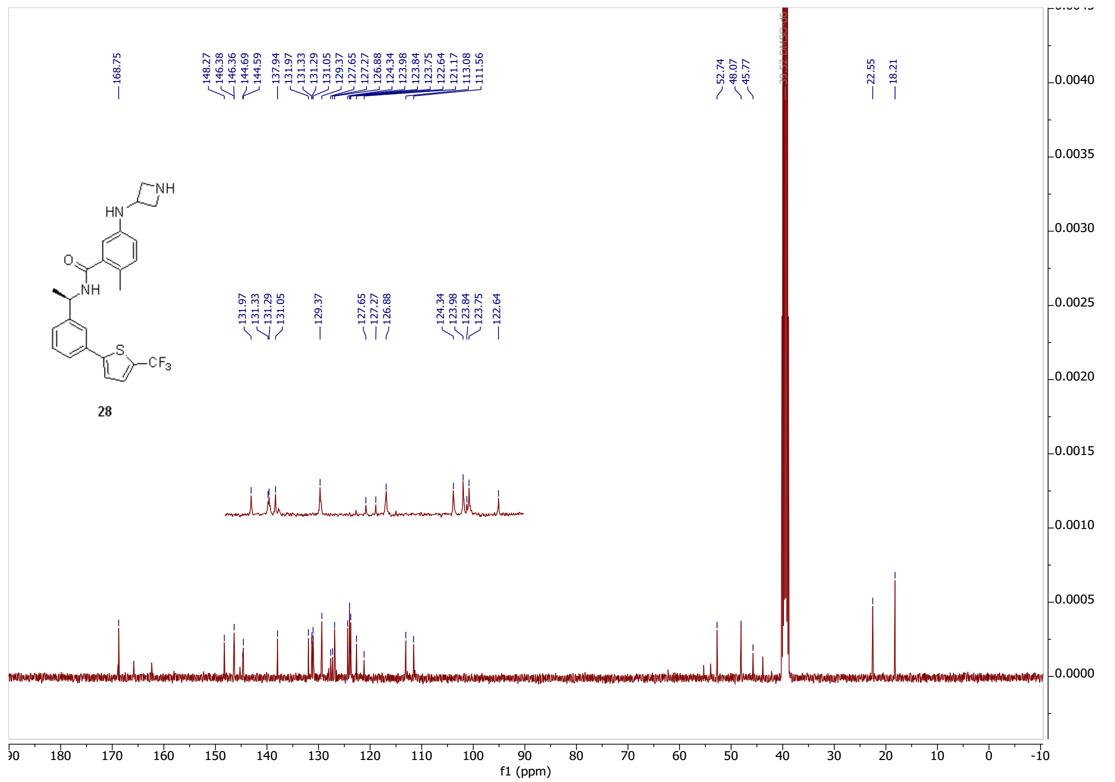

Compound **29**

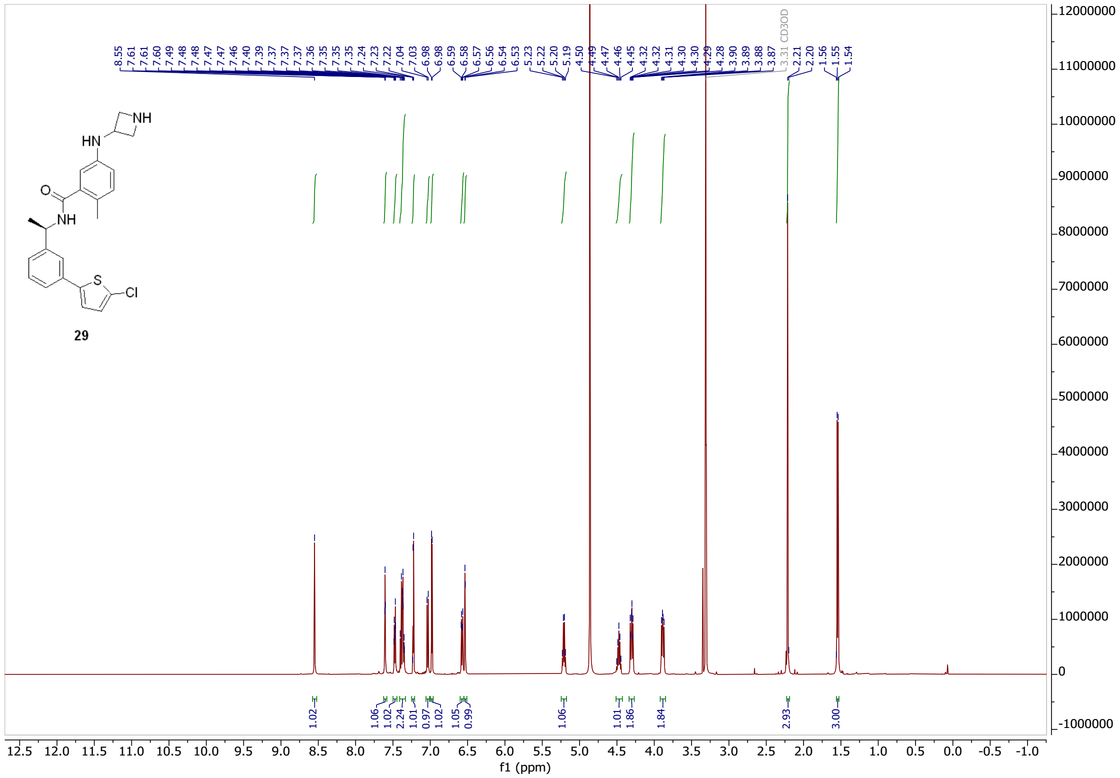

Compound **30**

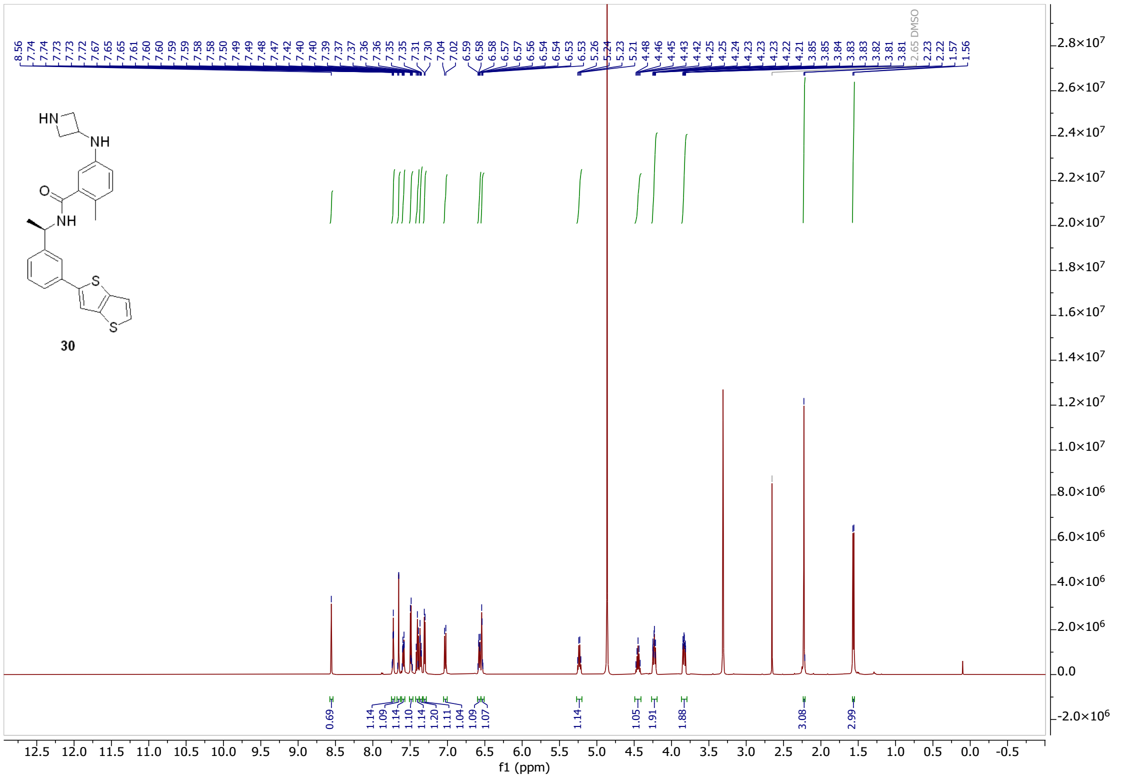

Compound **31**

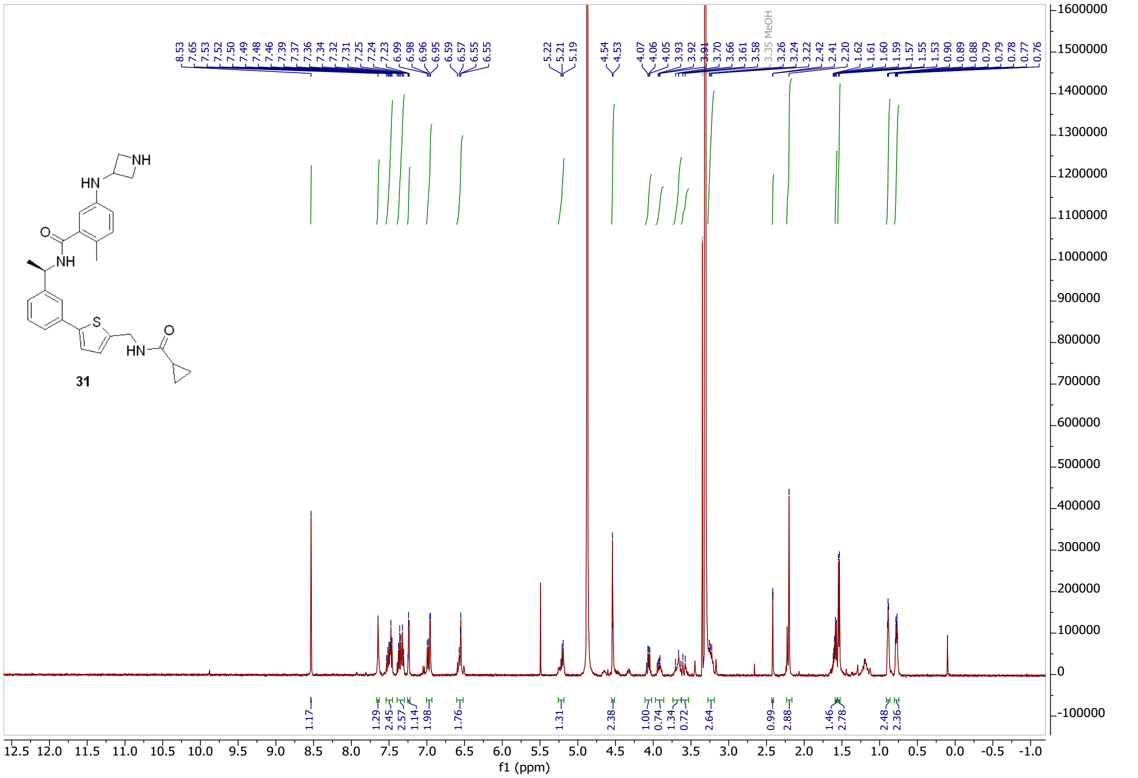

Compound **32**

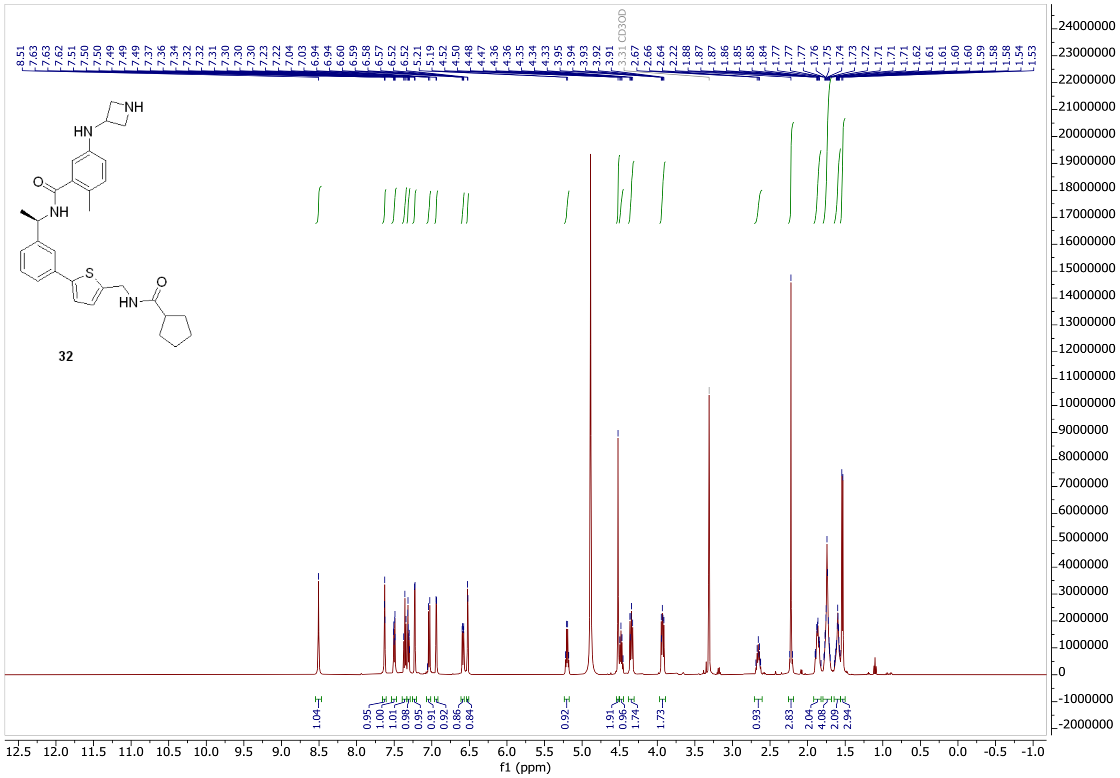

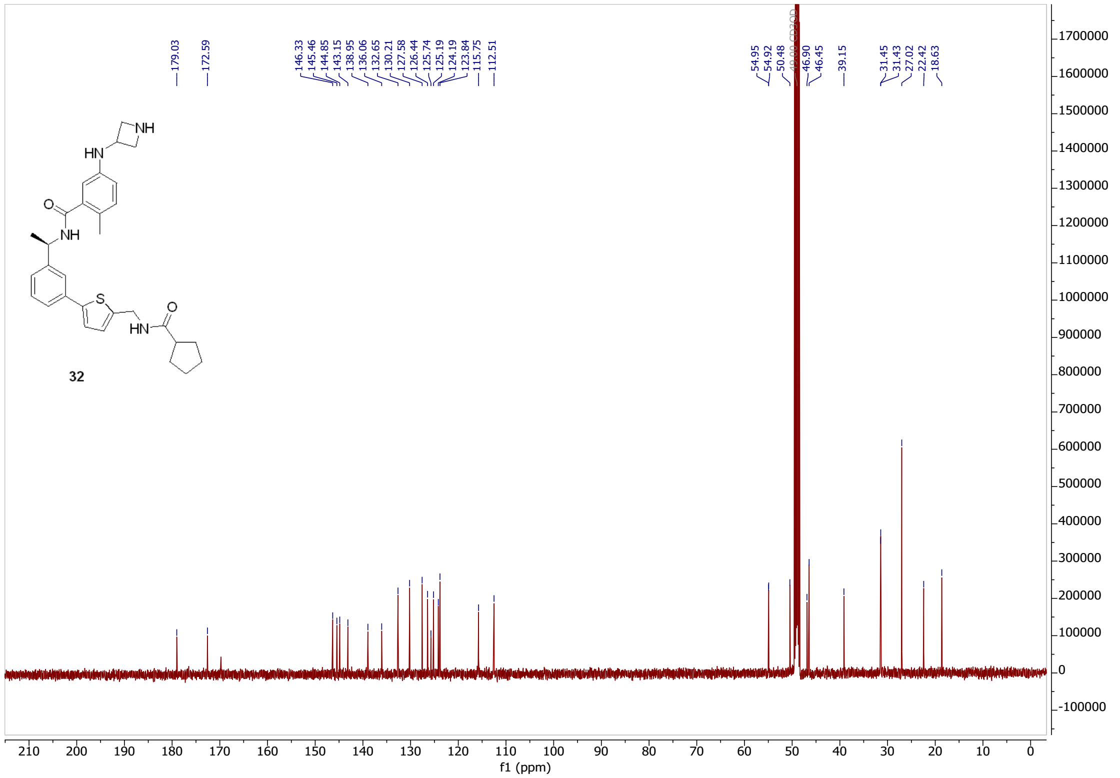

Compound **33**

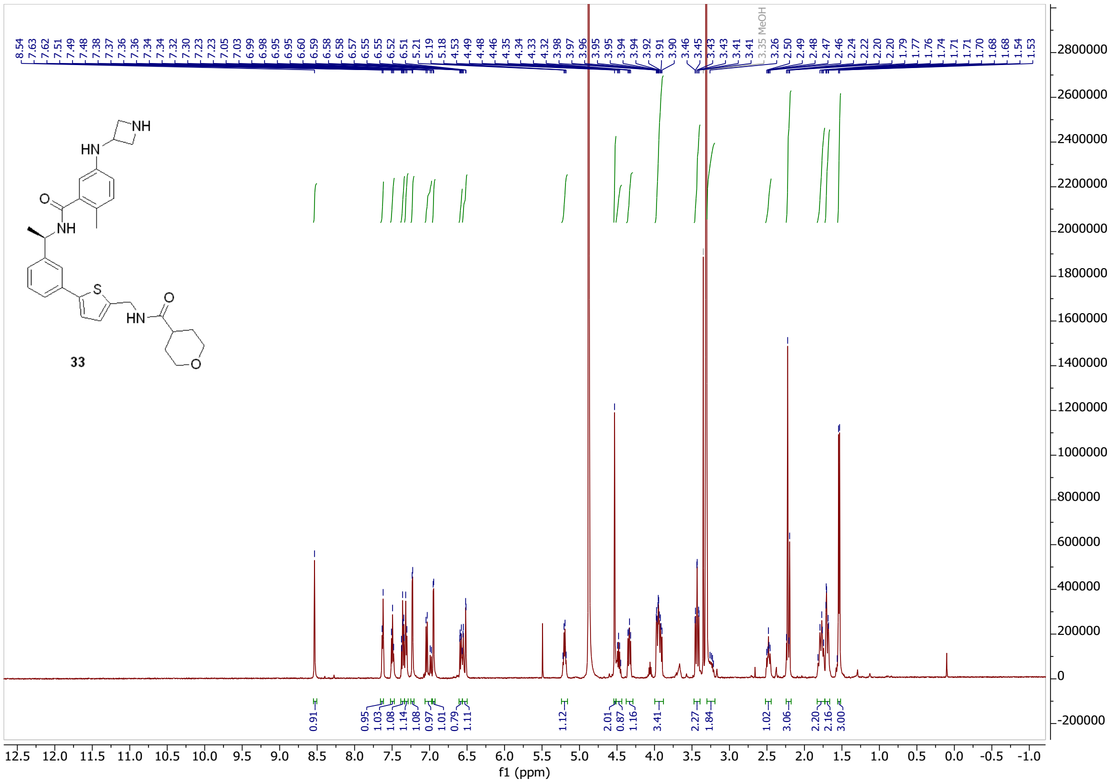

Compound **34**

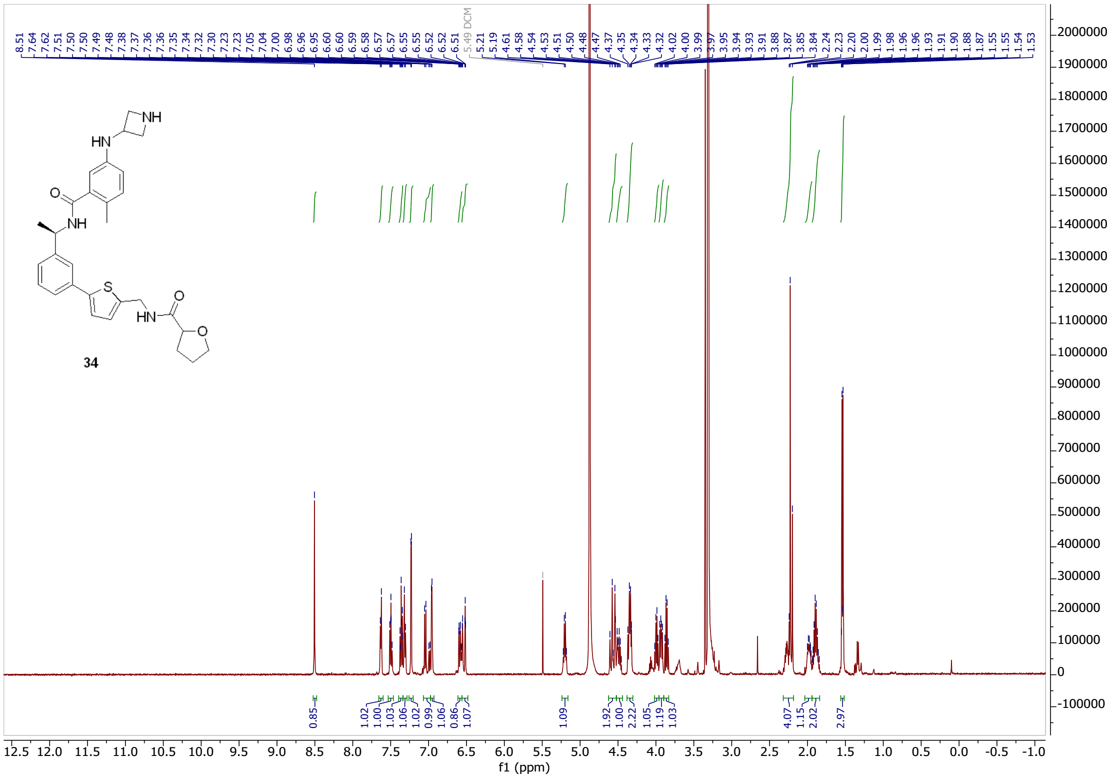

Compound **35**

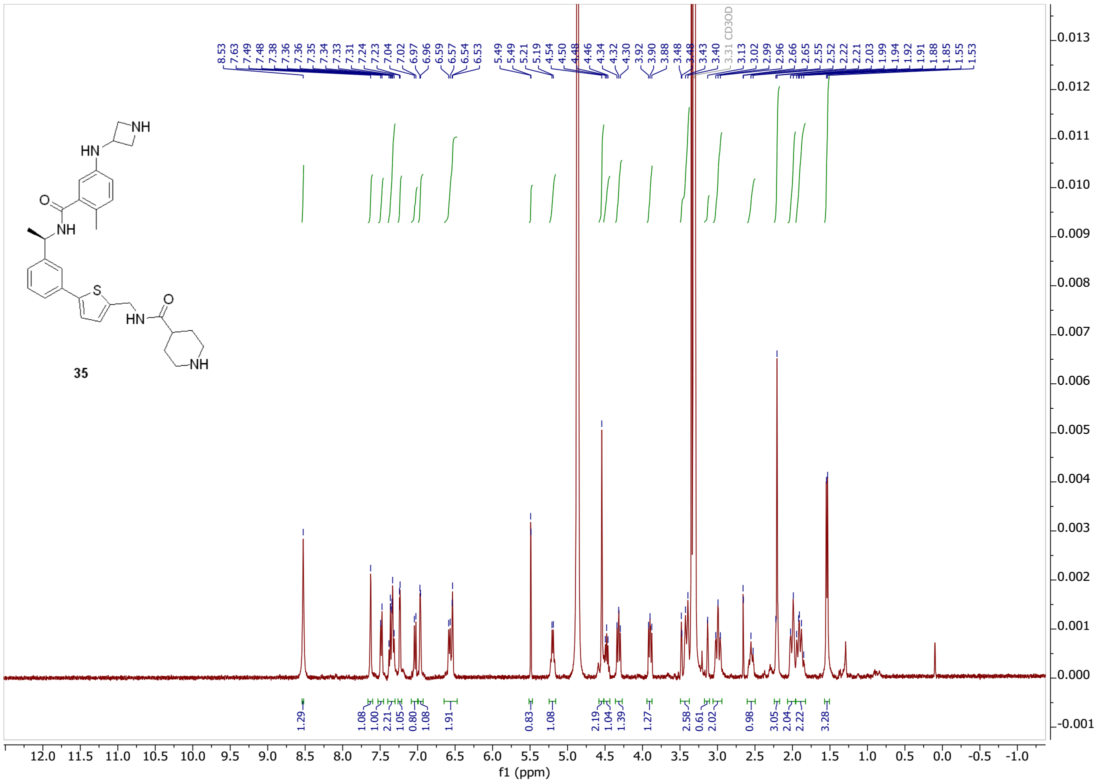

Compound **36**

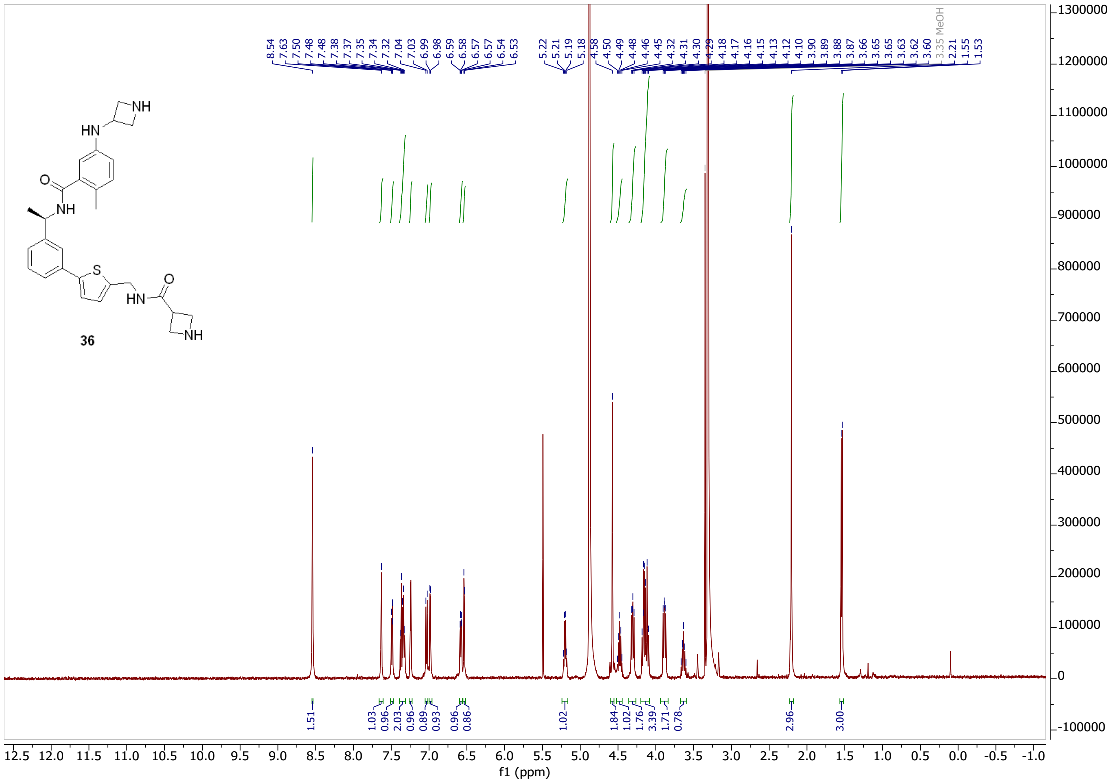

Compound **37**

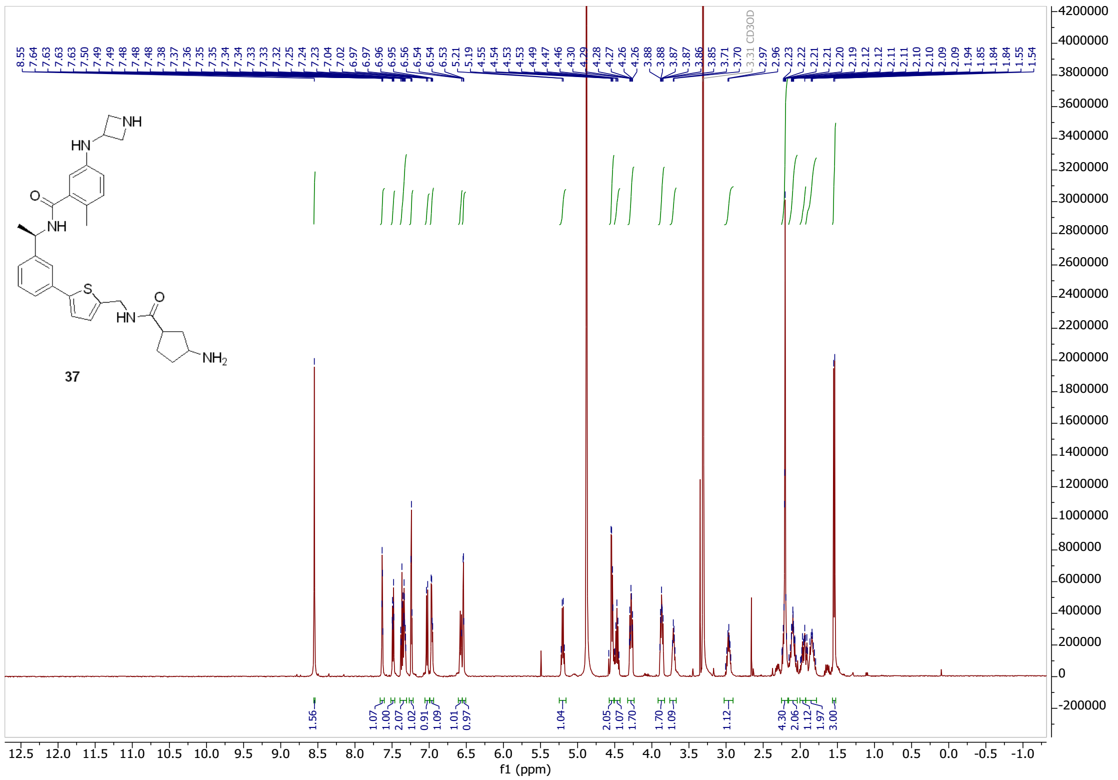

Compound **38**

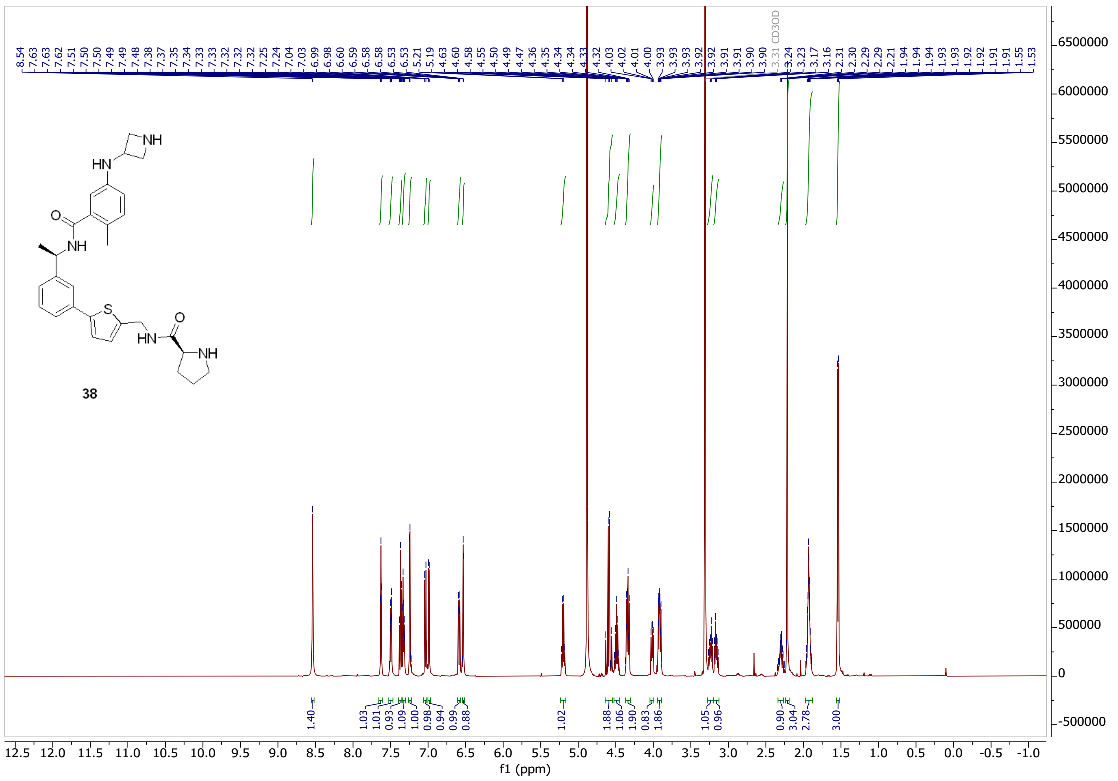

Compound **39**

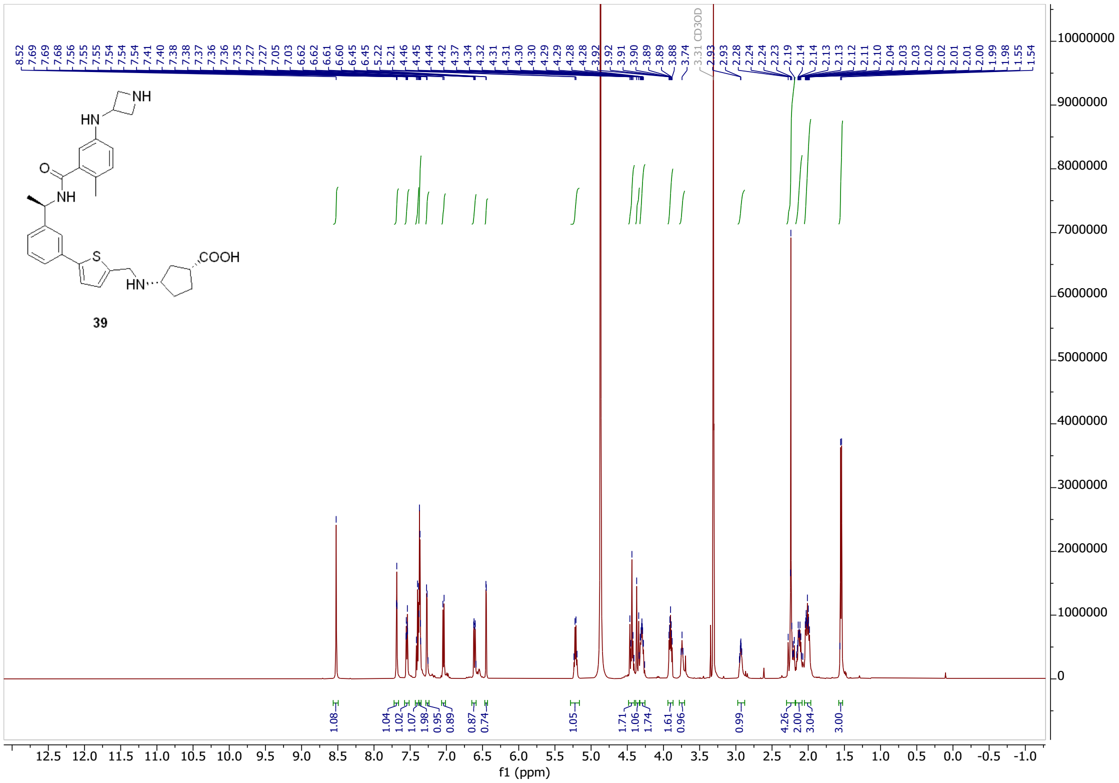

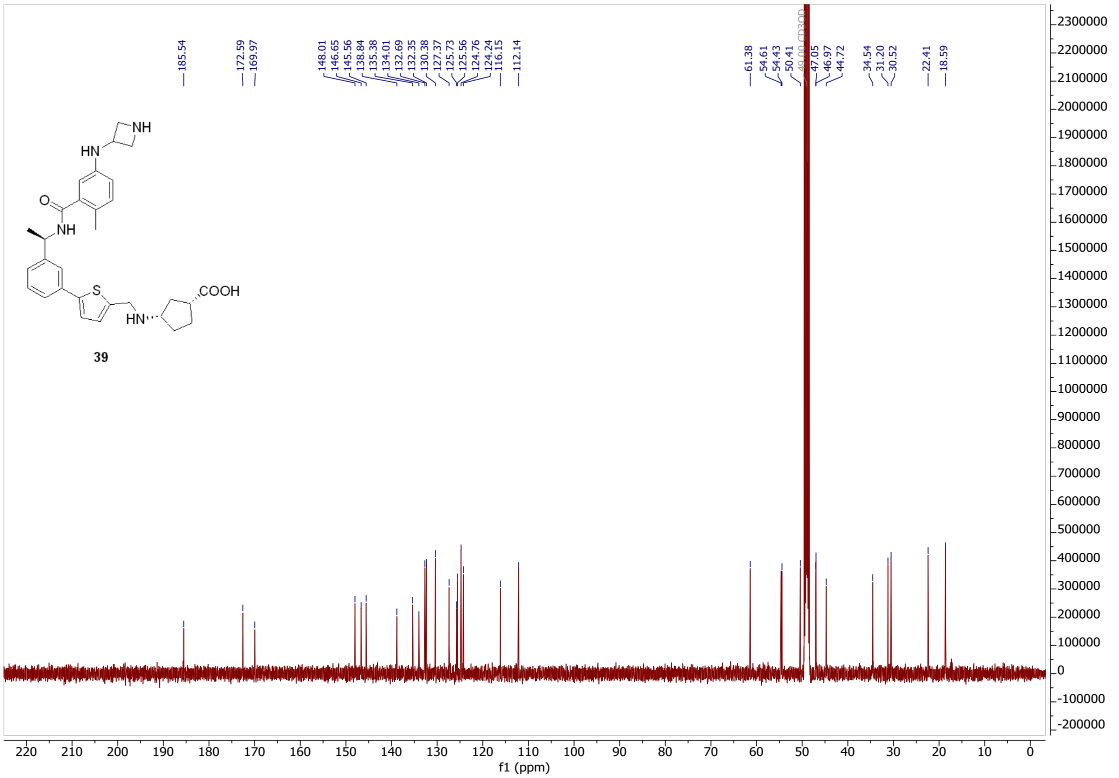

Compound **40**

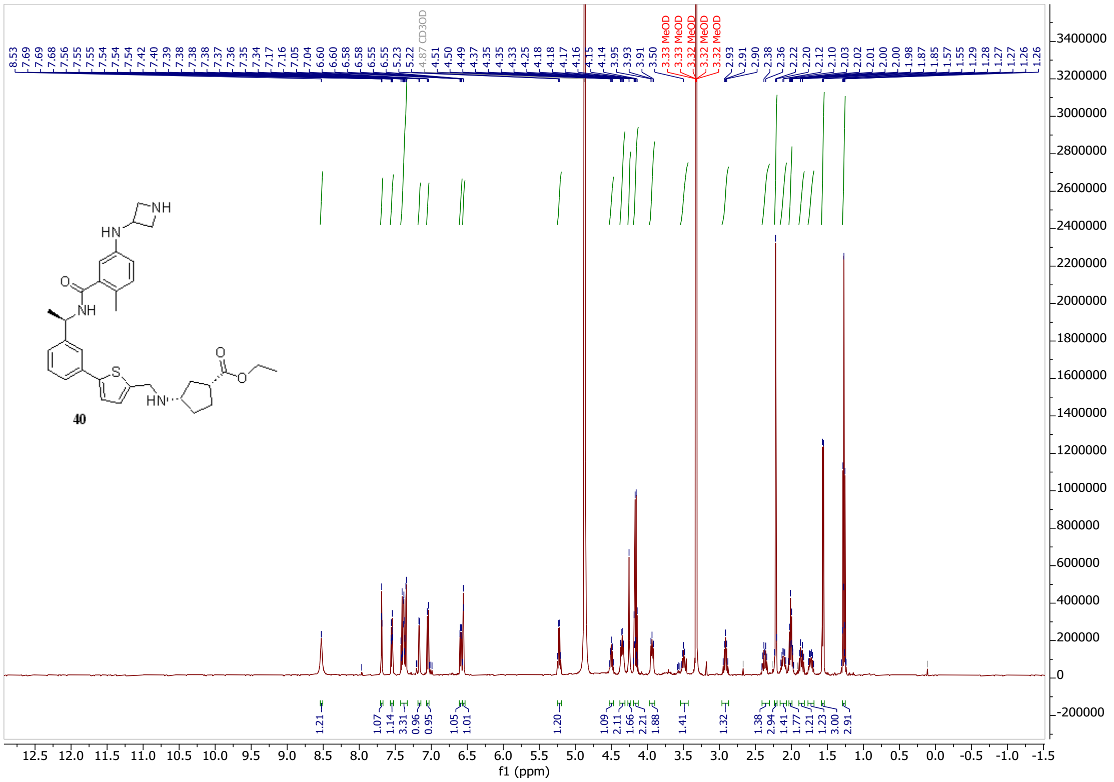

Compound **41**

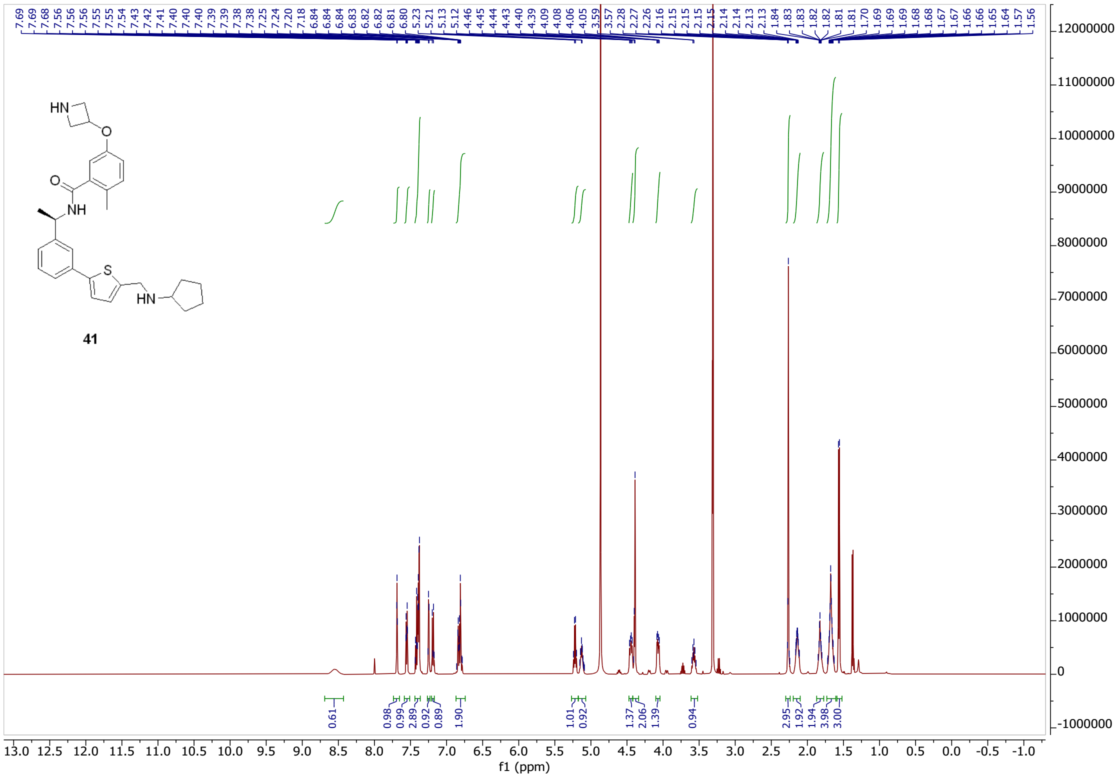

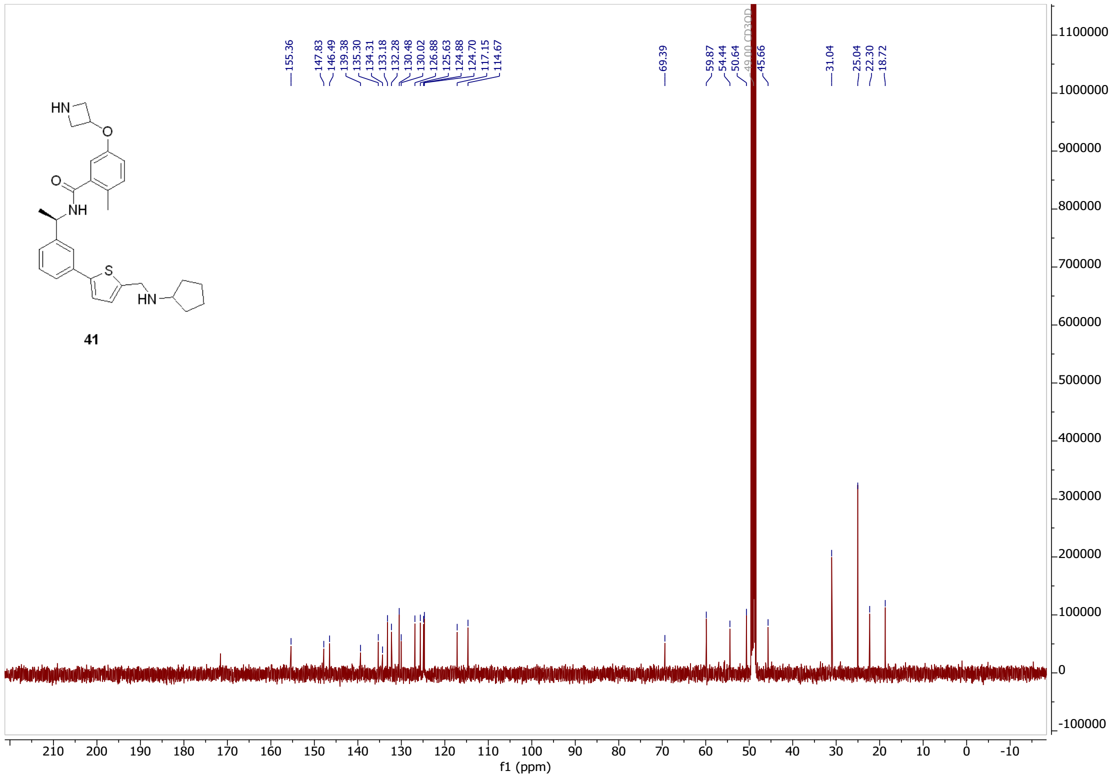

Compound **42**

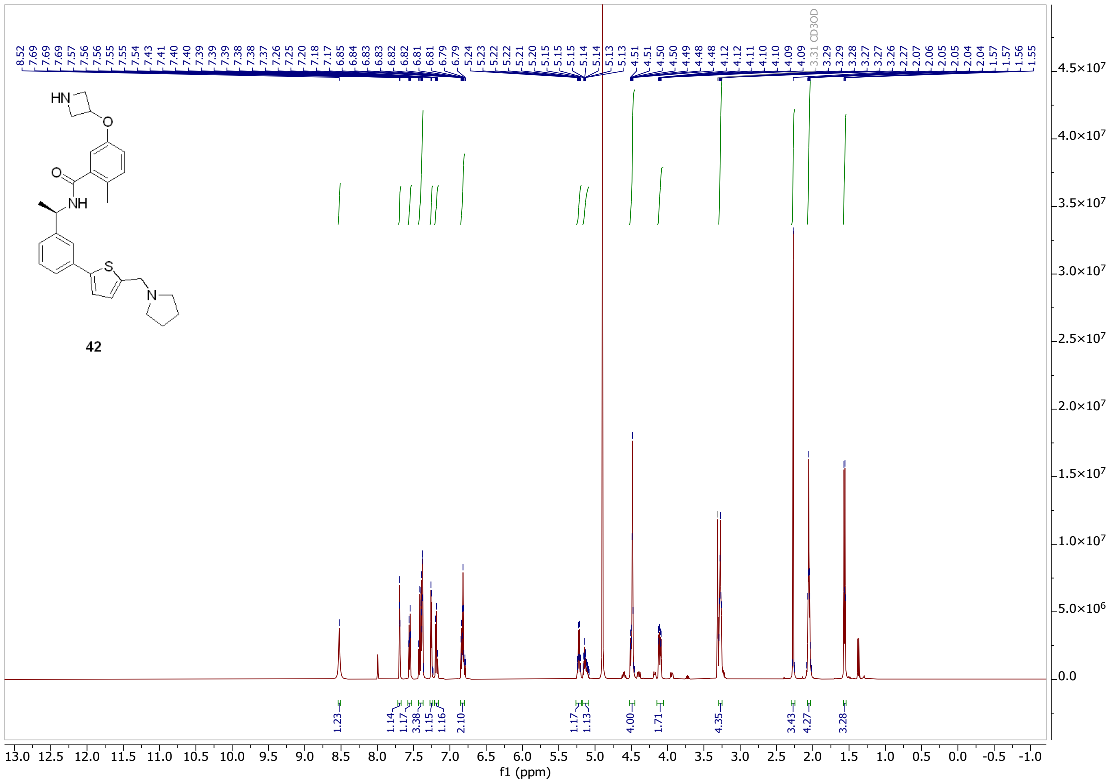

Compound **43**

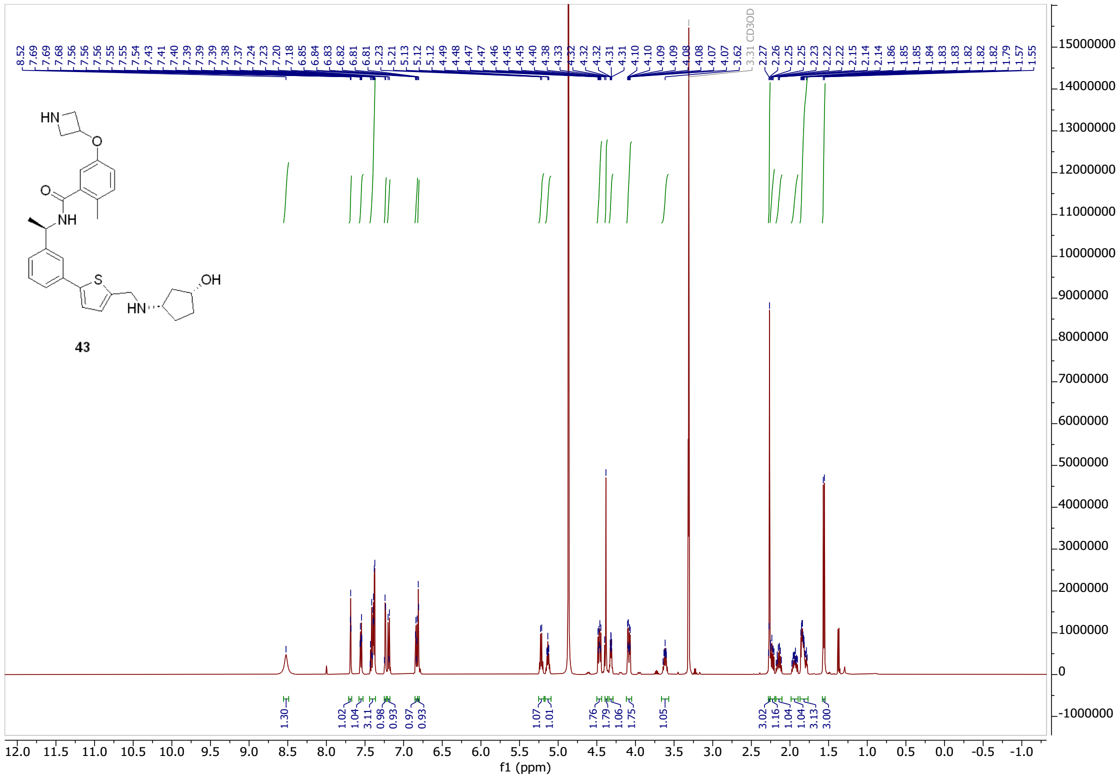

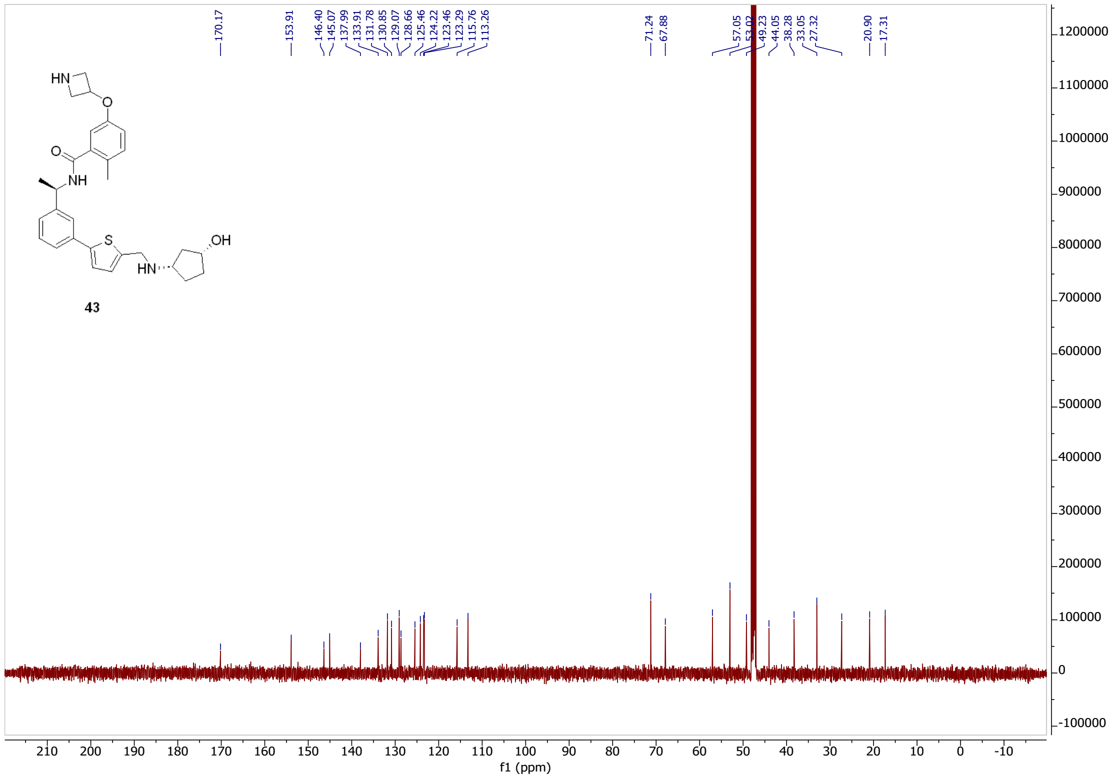

Compound **44**

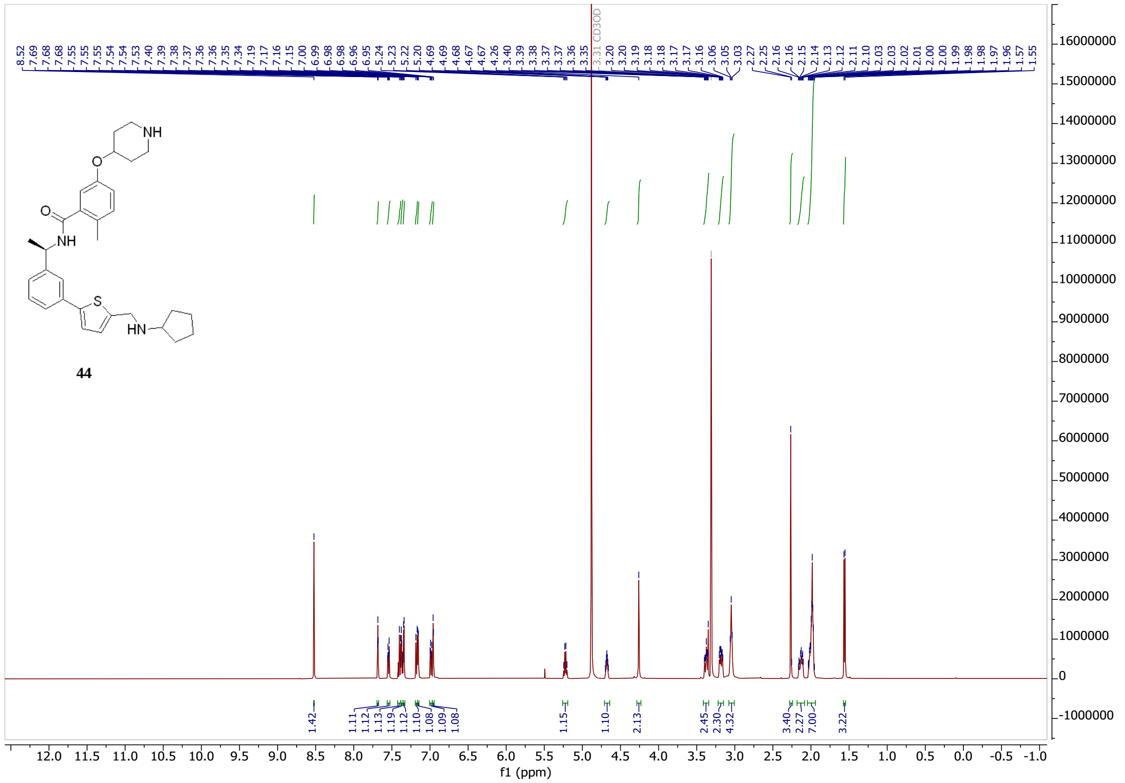

Compound **45**

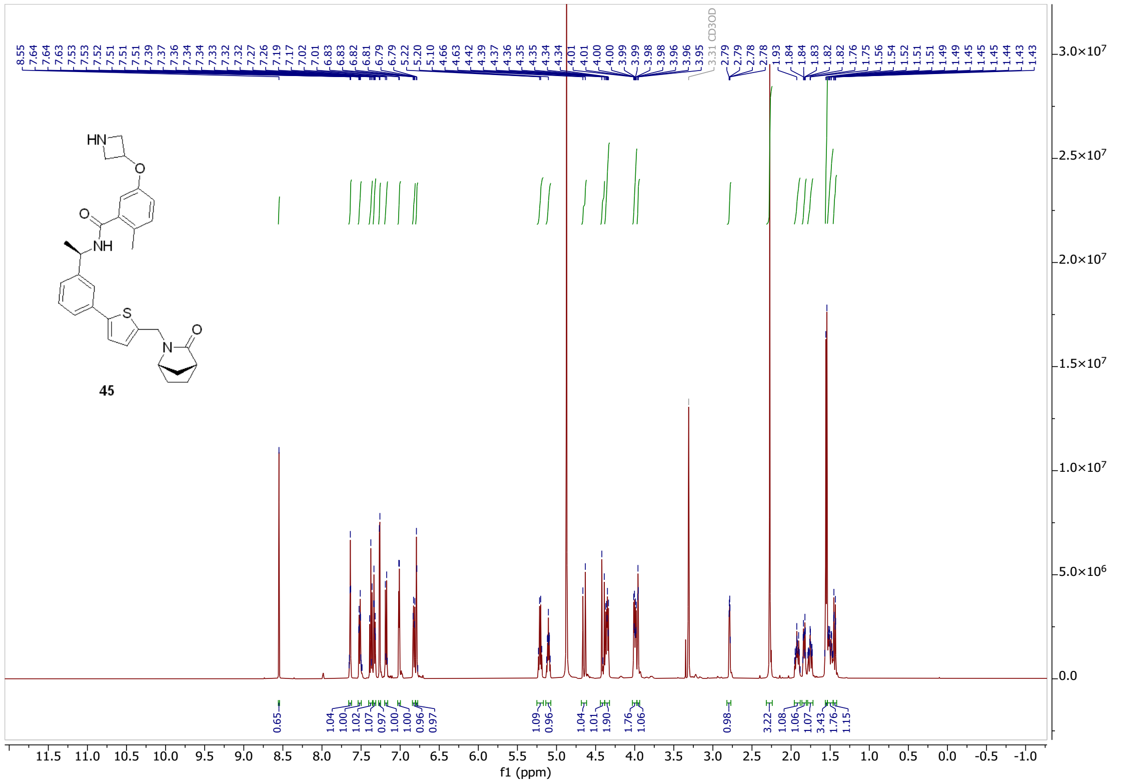

Compound **46**

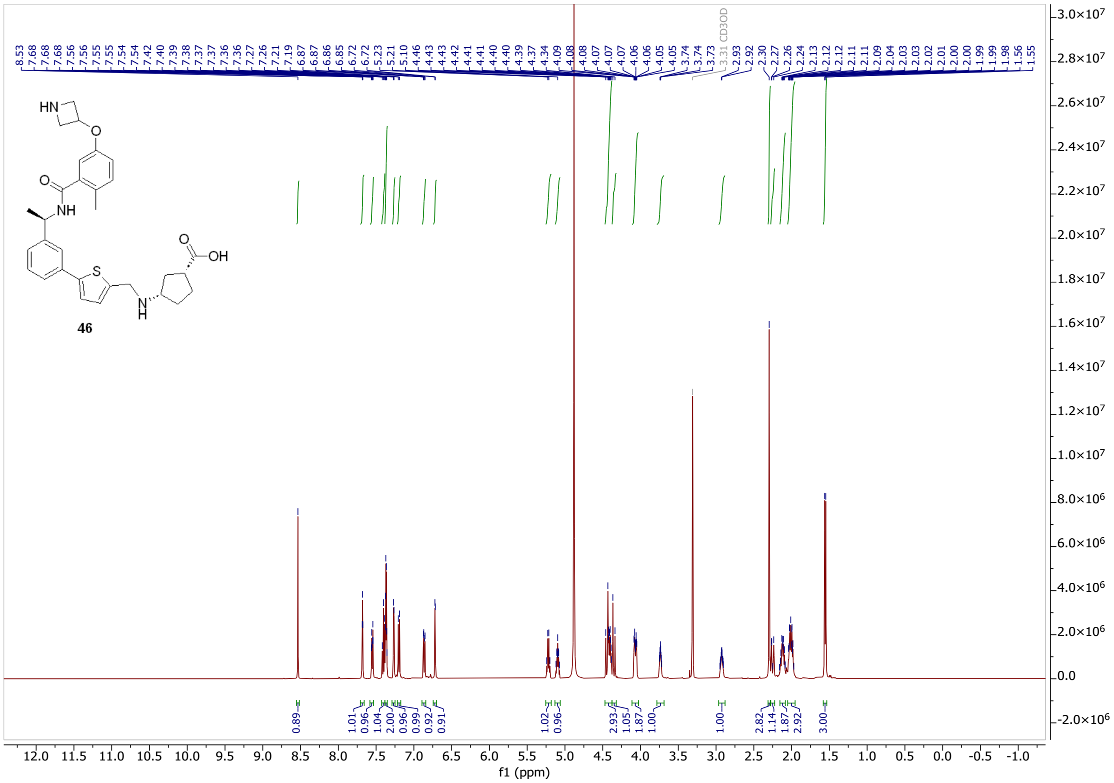

Compound **47**

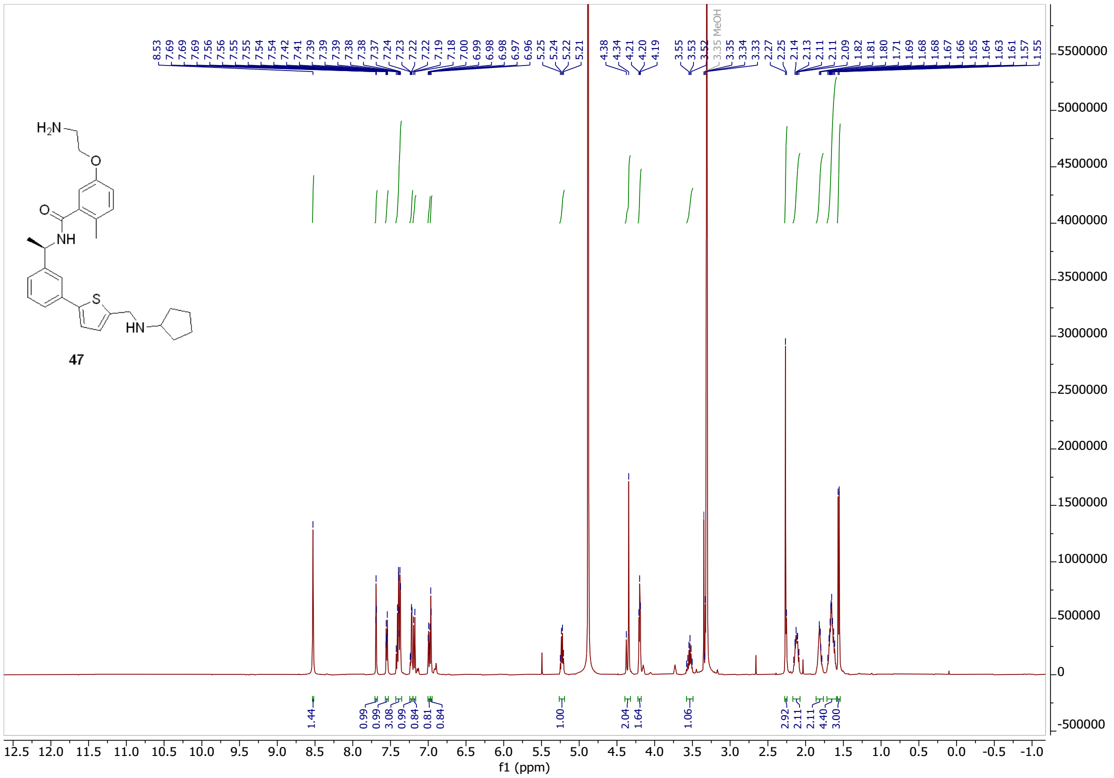

Compound **48**

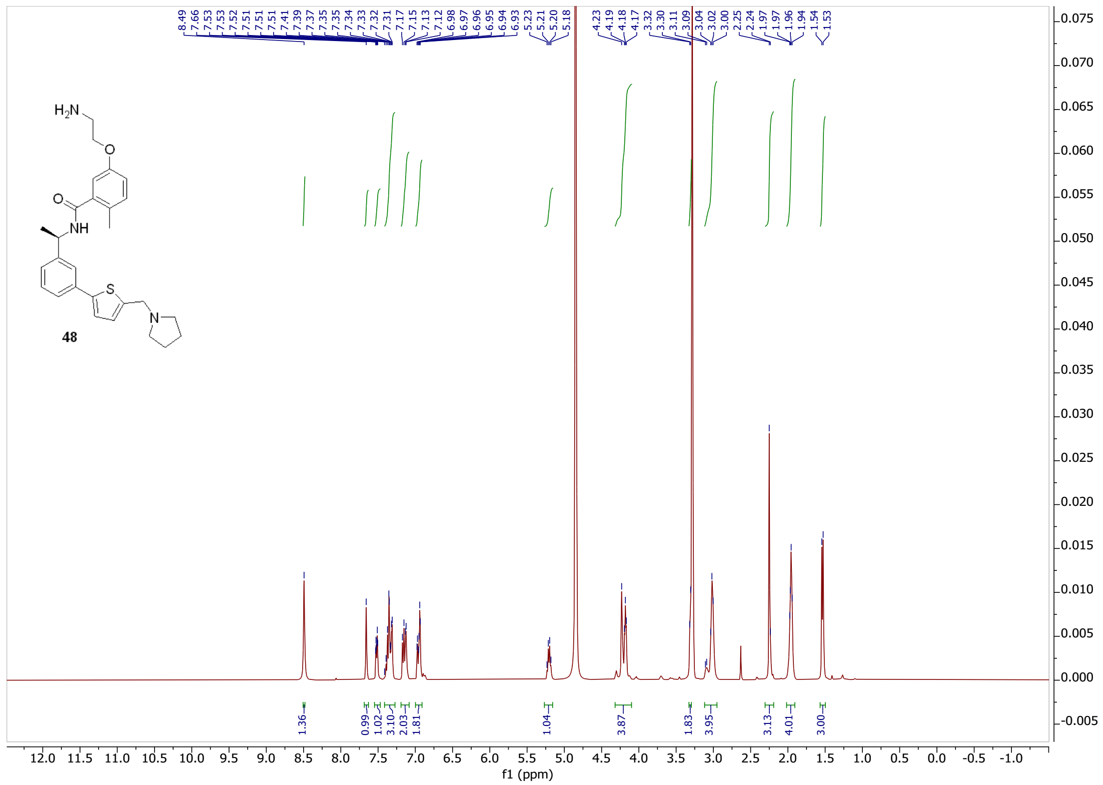

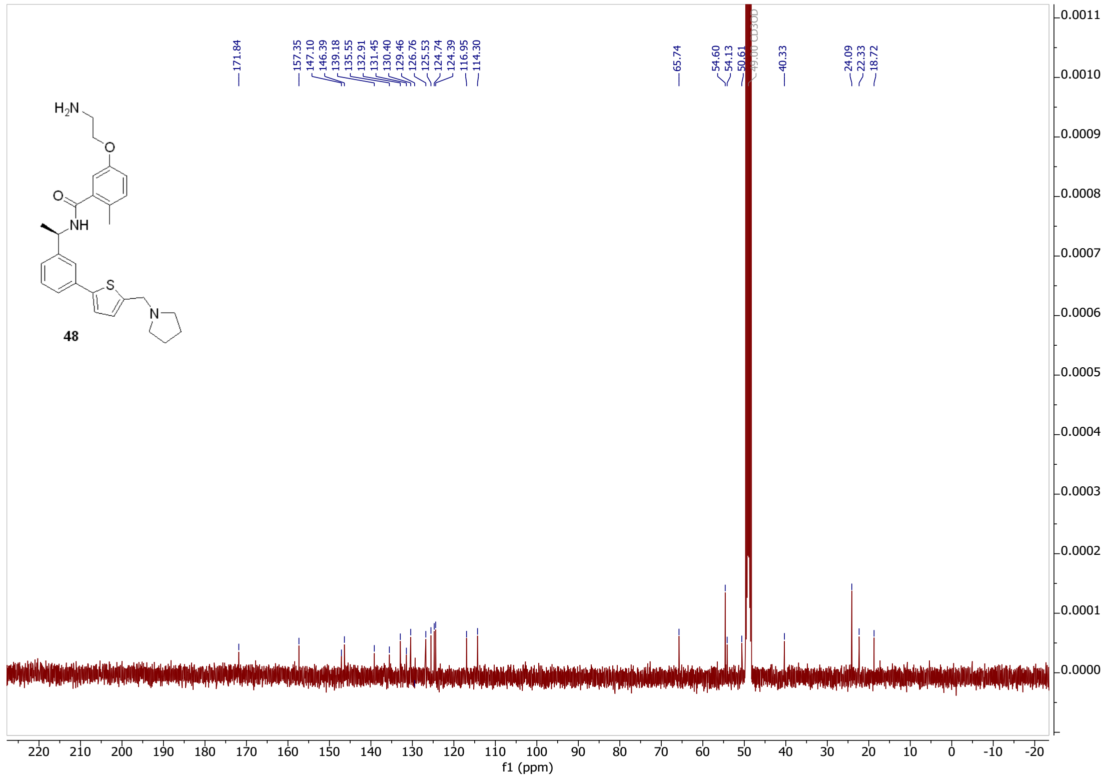

Compound **49**

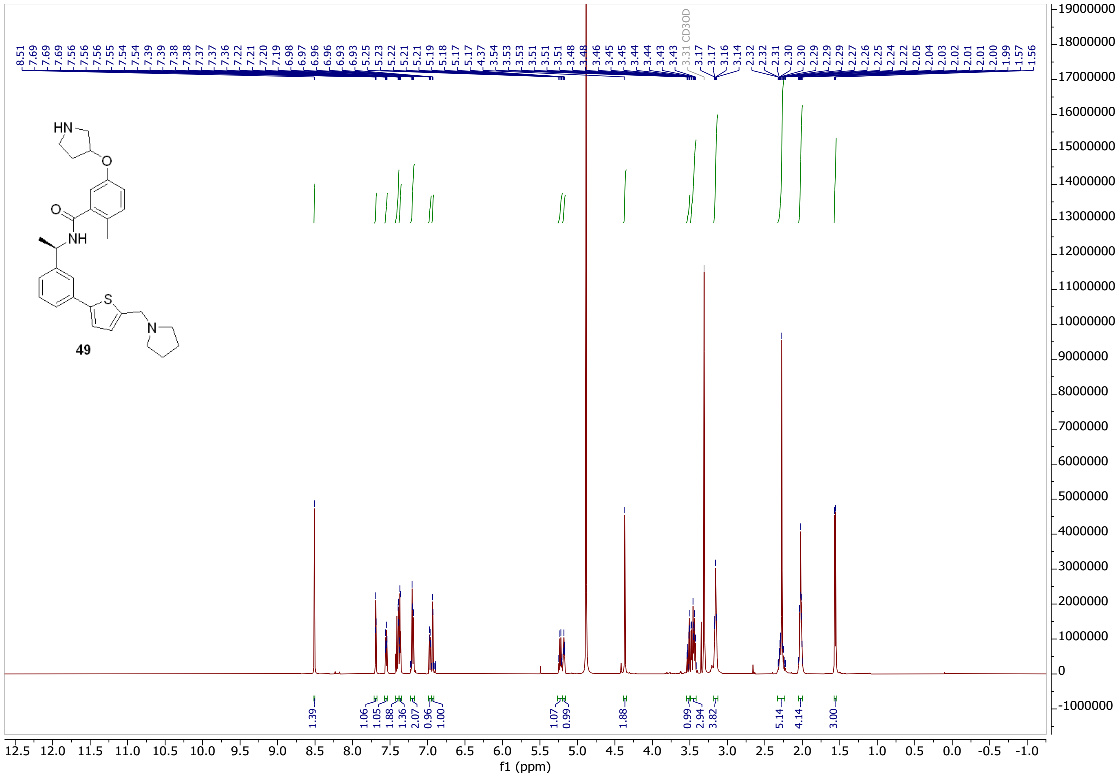

Compound **50**

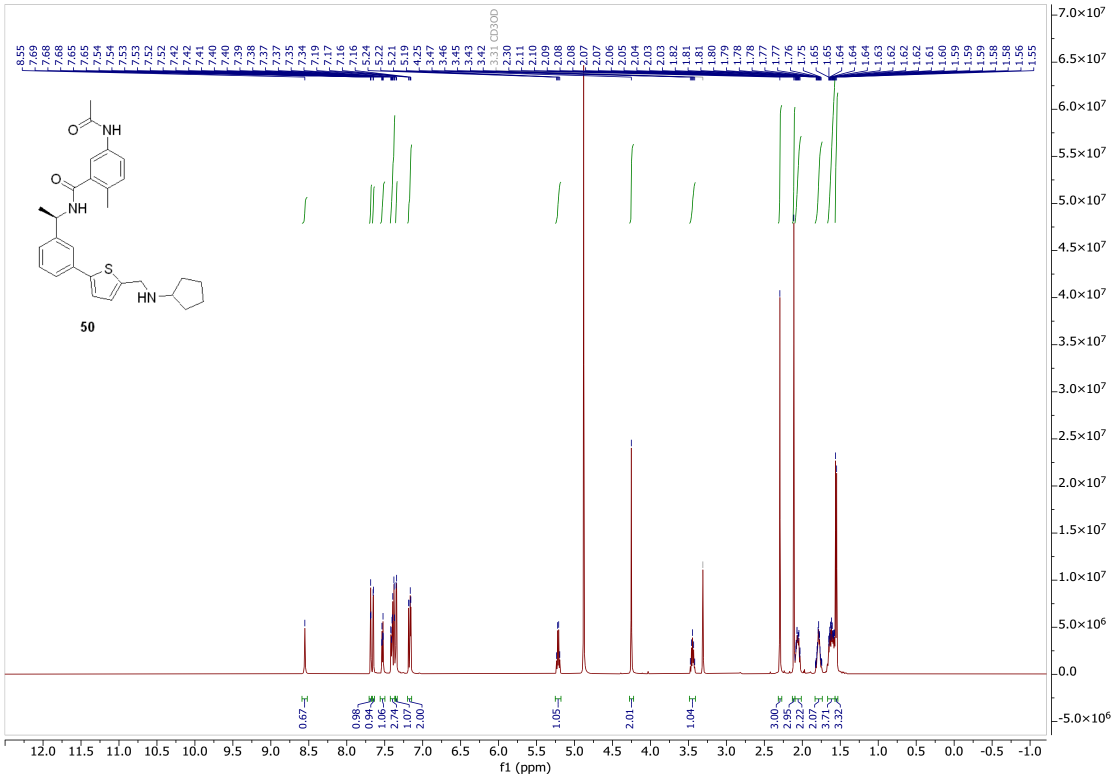

Compound **51**

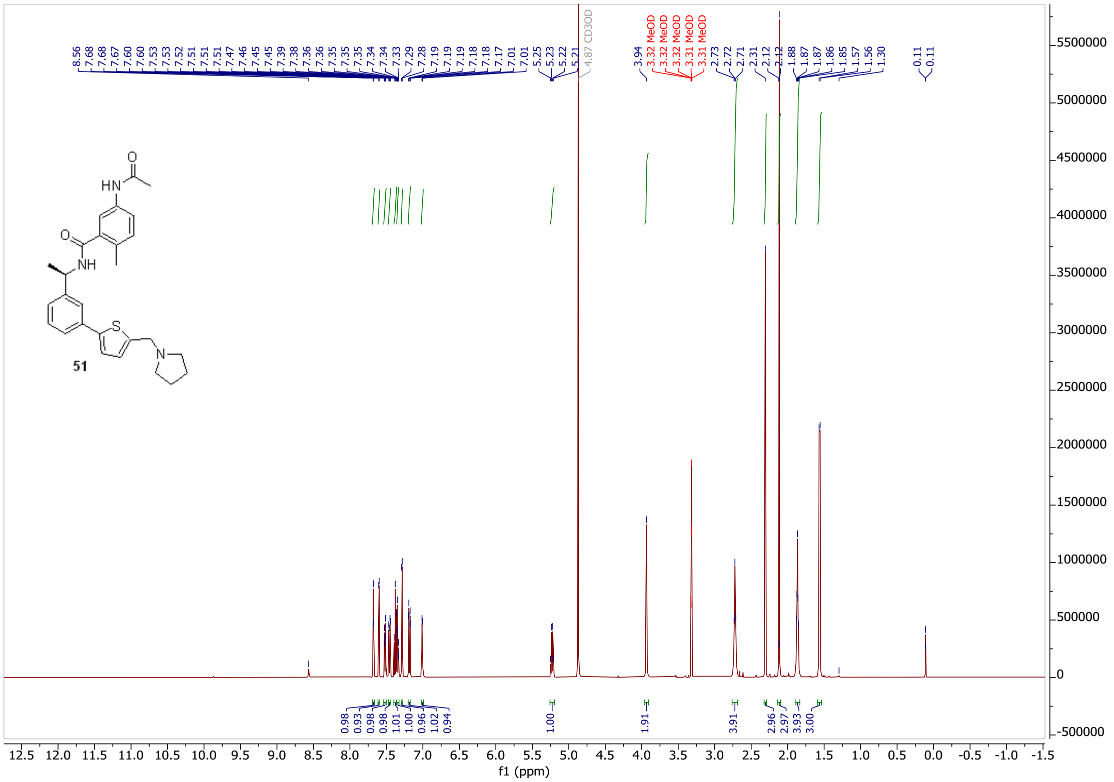

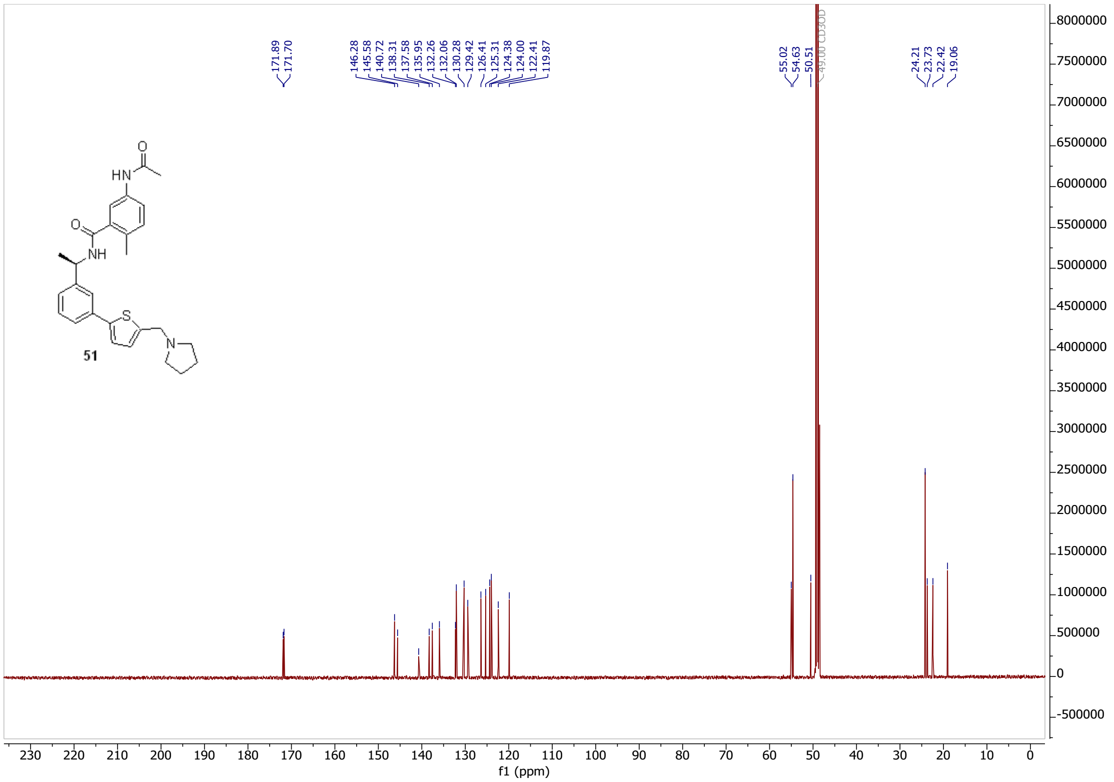

Compound **52**

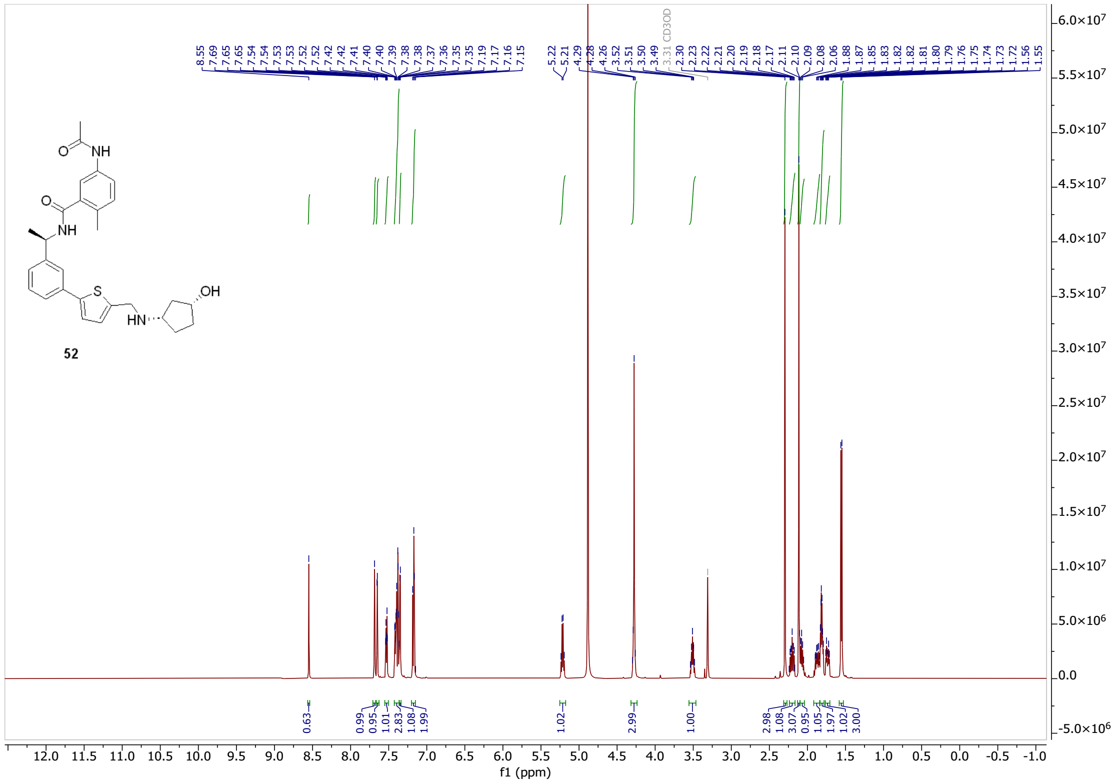

Compound **53**

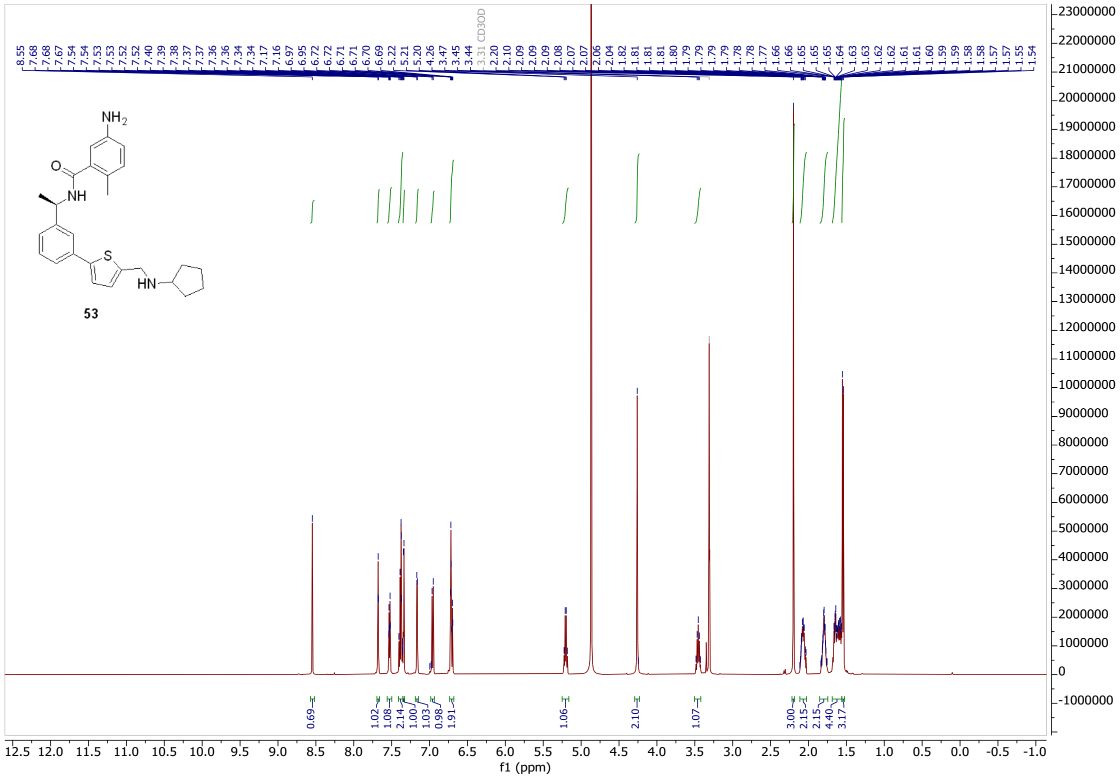

Compound **54**

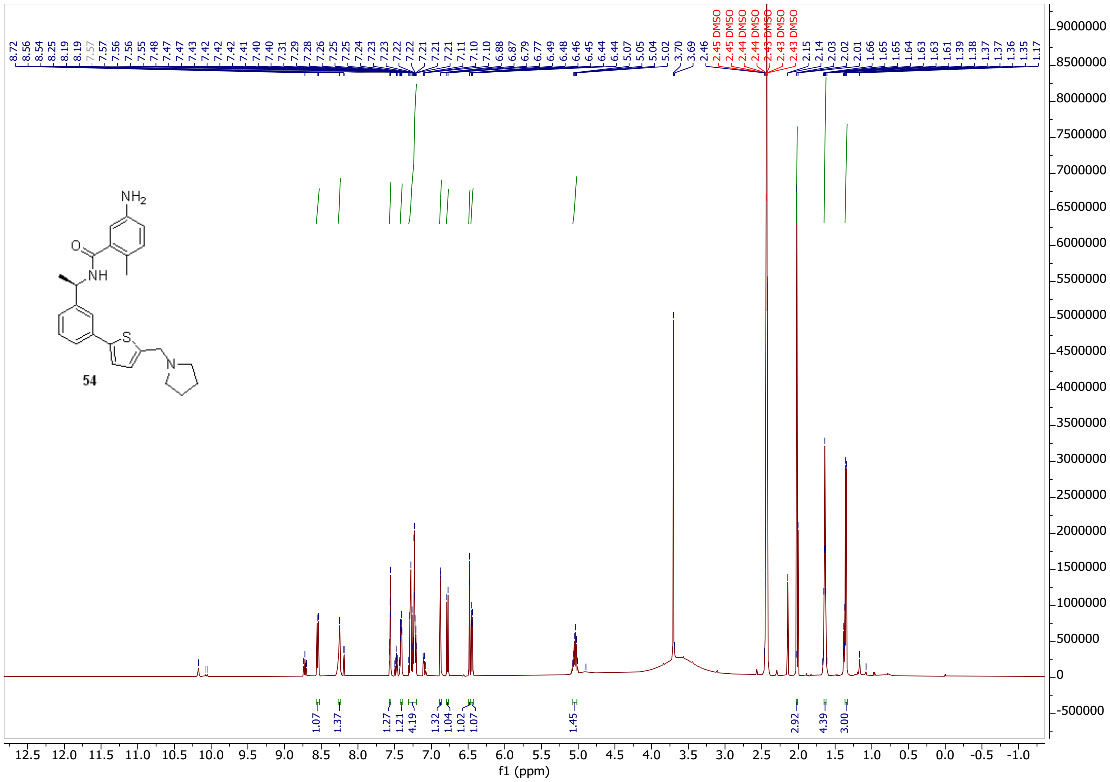

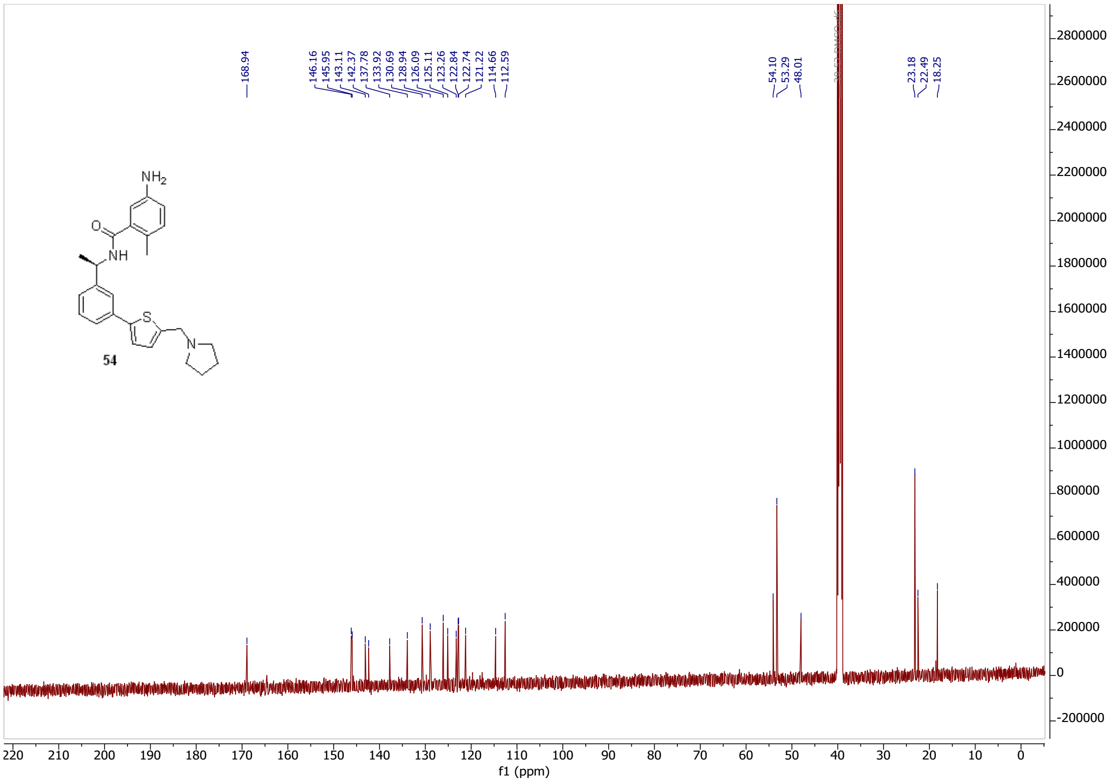

Compound **55**

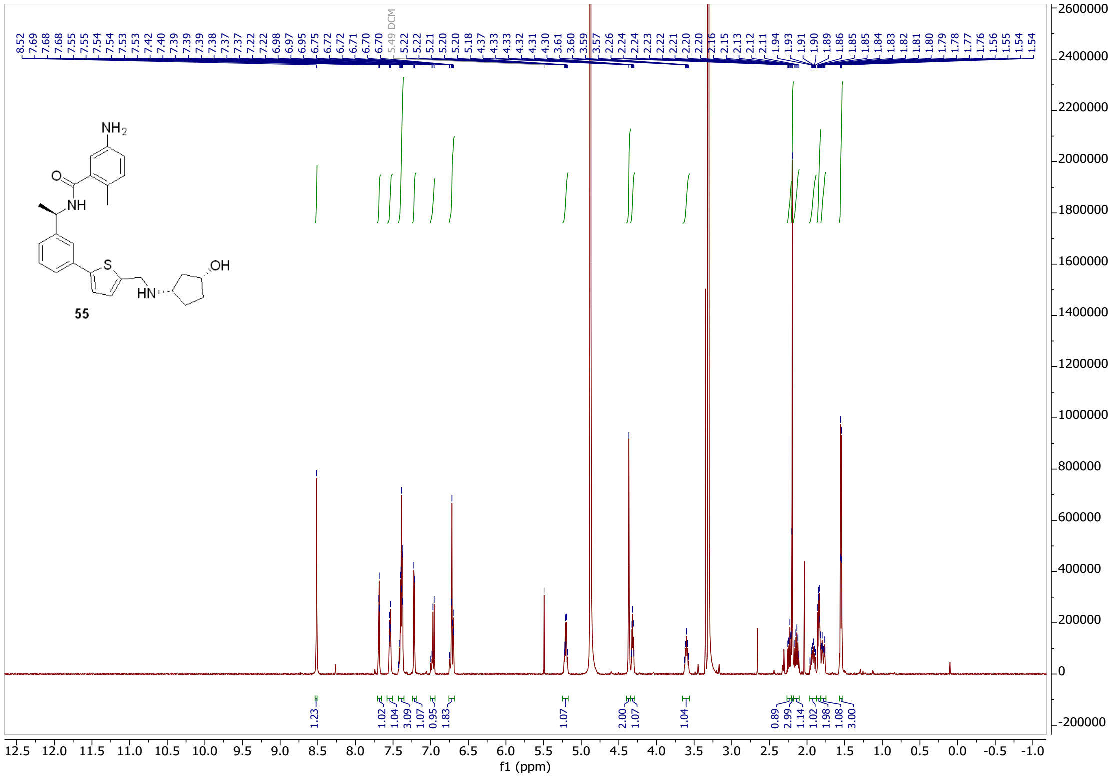

Compound **56**

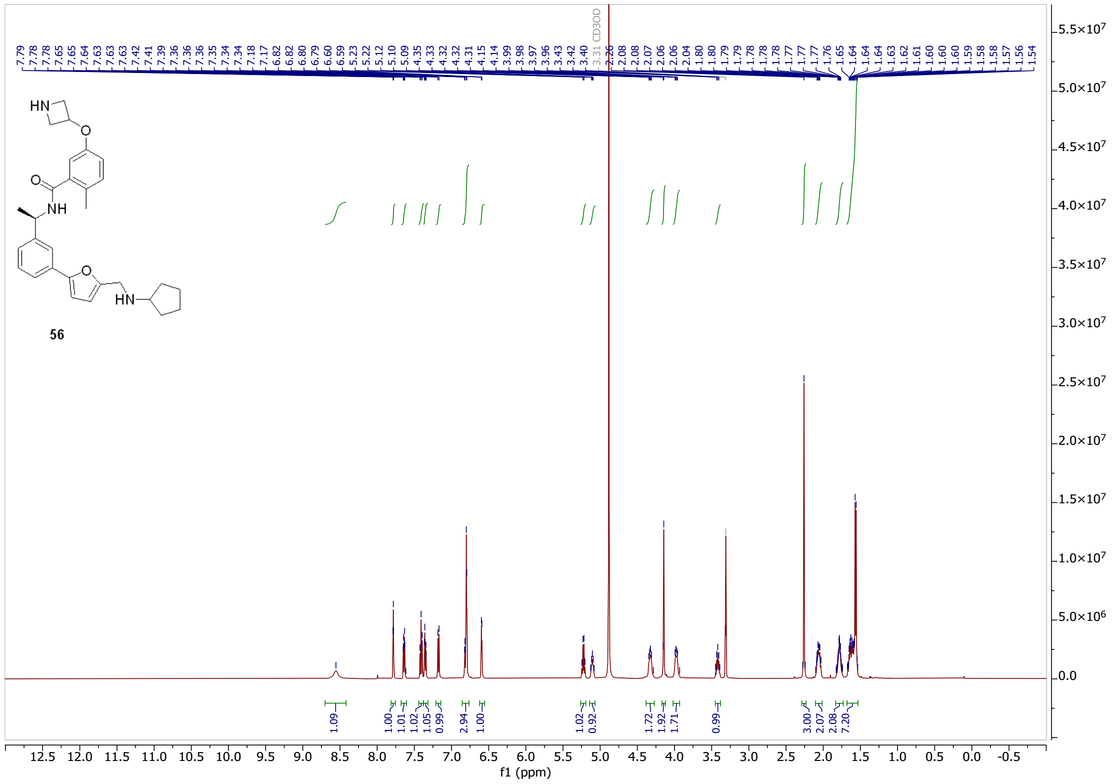

Compound **57**

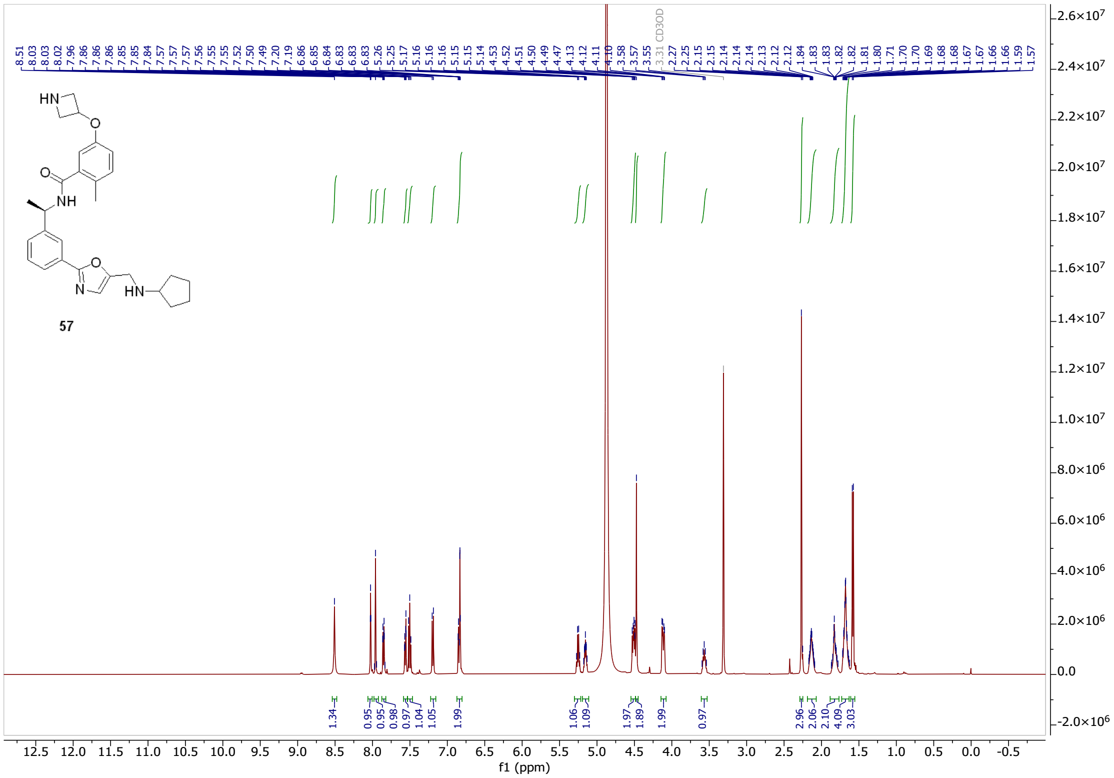

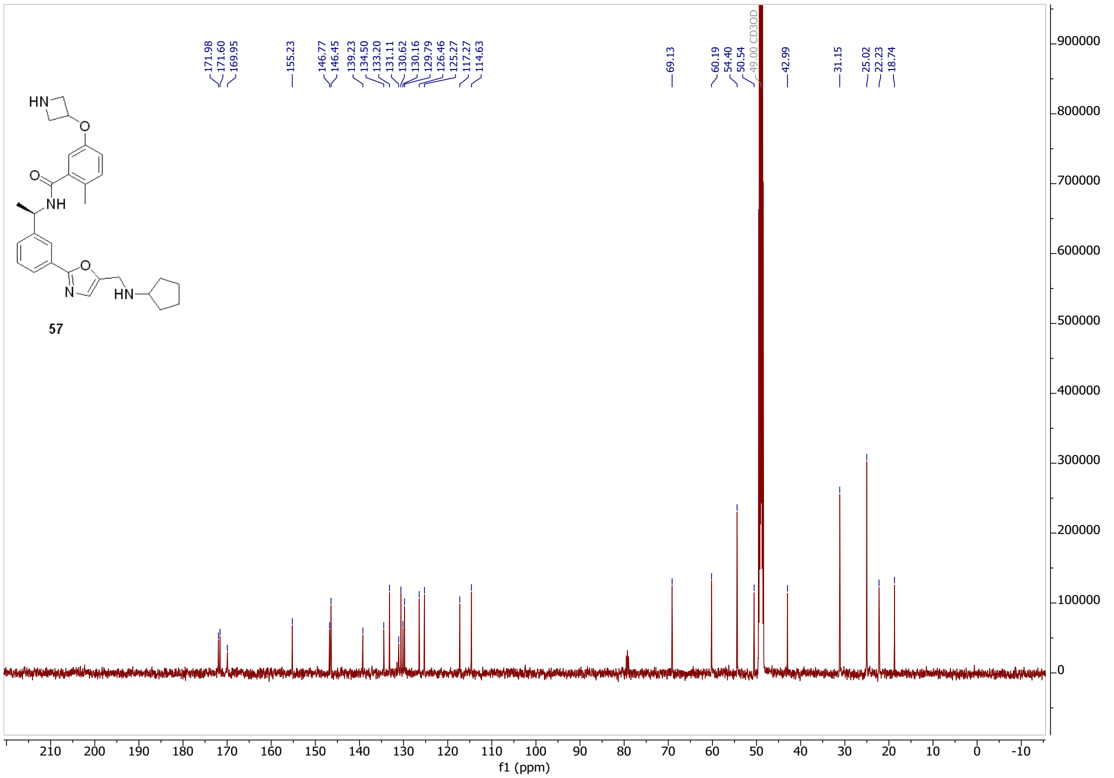

Compound **58**

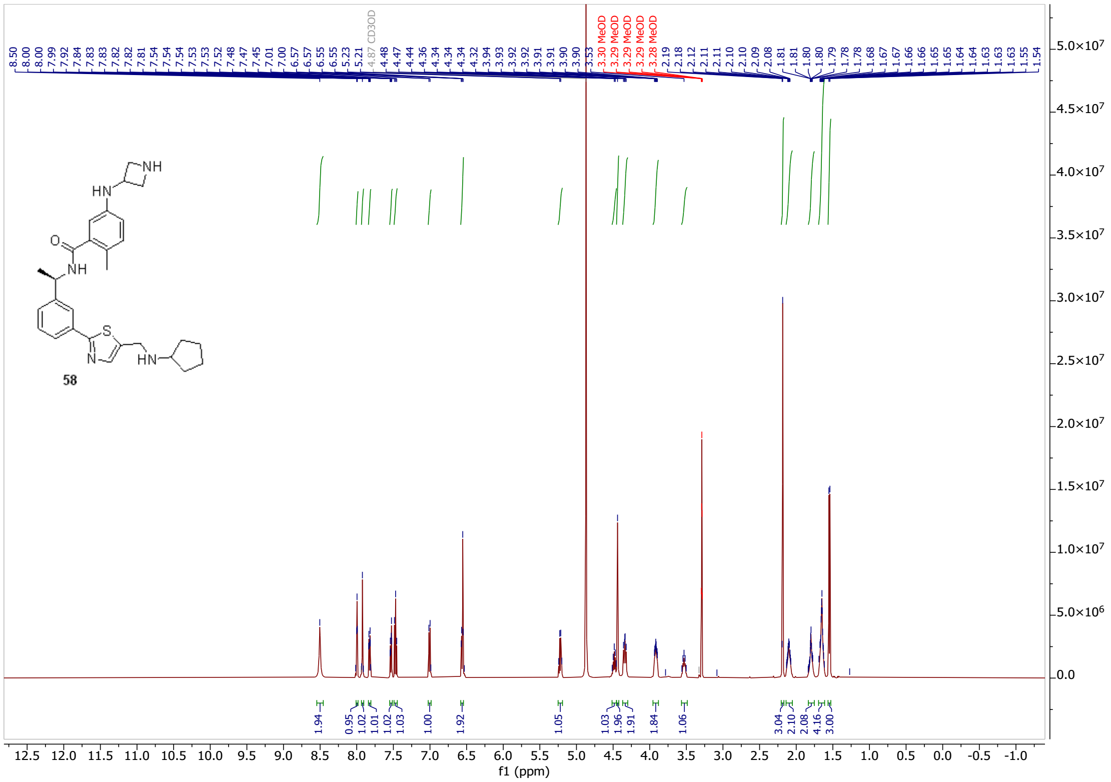
8.50 8.00 8.00 7.99 7.92 7.84 7.83 7.83 7.82 7.82 7.81 7.54 7.54 7.54 7.53 7.53 7.52 7.48 7.47 7.45 7.01 7.00 6.57 6.57 6.55 6.55 5.23 5.21 4.87 CD3OD 4.48 4.47 4.44 4.36 4.34 4.34 4.34 4.32 3.94 3.93 3.92 3.92 3.91 3.91 3.90 3.90 3.53 3.30 MeOD 3.29 MeOD 3.29 MeOD 3.29 MeOD 3.28 MeOD 2.19 2.18 2.12 2.11 2.11 2.10 2.10 2.09 2.08 1.81 1.81 1.80 1.80 1.79 1.78 1.68 1.67 1.67 1.66 1.66 1.65 1.65 1.64 1.64 1.63 1.63 1.55 1.54
HN
NH
O
NH
S
N
HN
58
1.94 0.95 1.02 1.01 1.02 1.03 1.00 1.92 1.05 1.03 1.96 1.91 1.84 1.06 3.04 2.10 2.08 4.16 3.00
f1 (ppm)

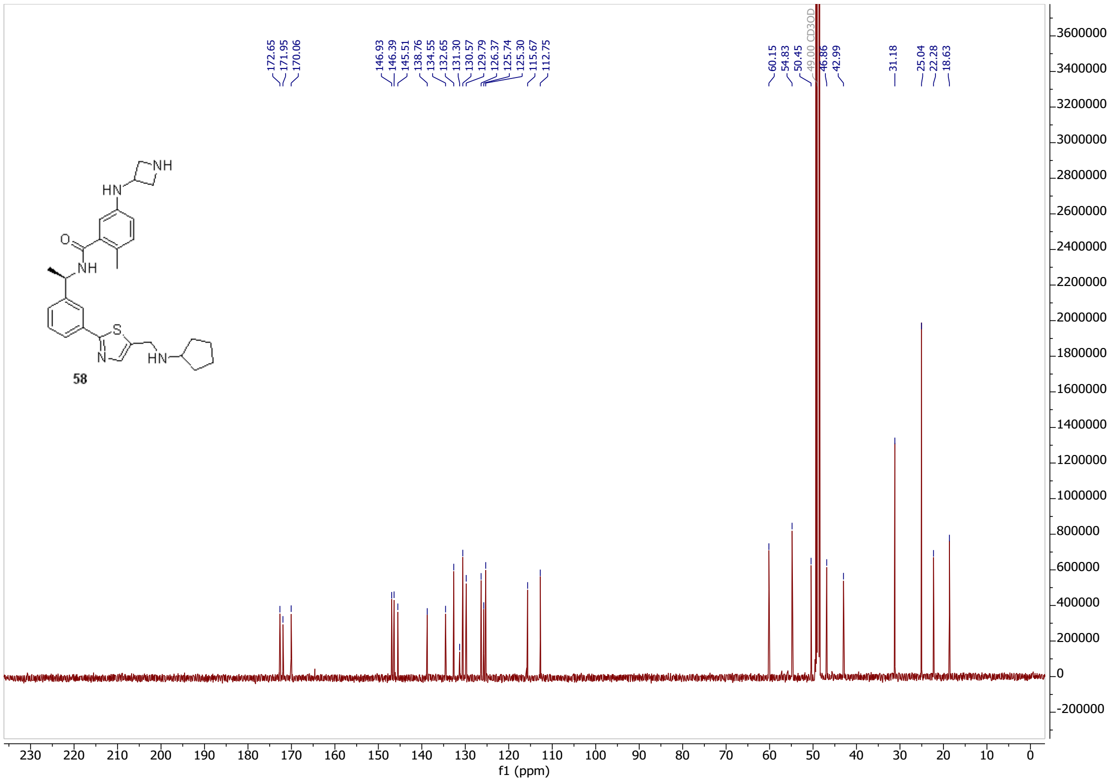
172.65 171.95 170.06 146.93 146.39 145.51 138.76 134.55 132.65 131.30 130.57 129.79 126.37 125.74 125.30 115.67 112.75 60.15 54.83 50.45 49.00 CD3OD 46.86 42.99 31.18 25.04 22.28 18.63
HN
NH
O
NH
S
N
HN
58
f1 (ppm)

Compound **59**

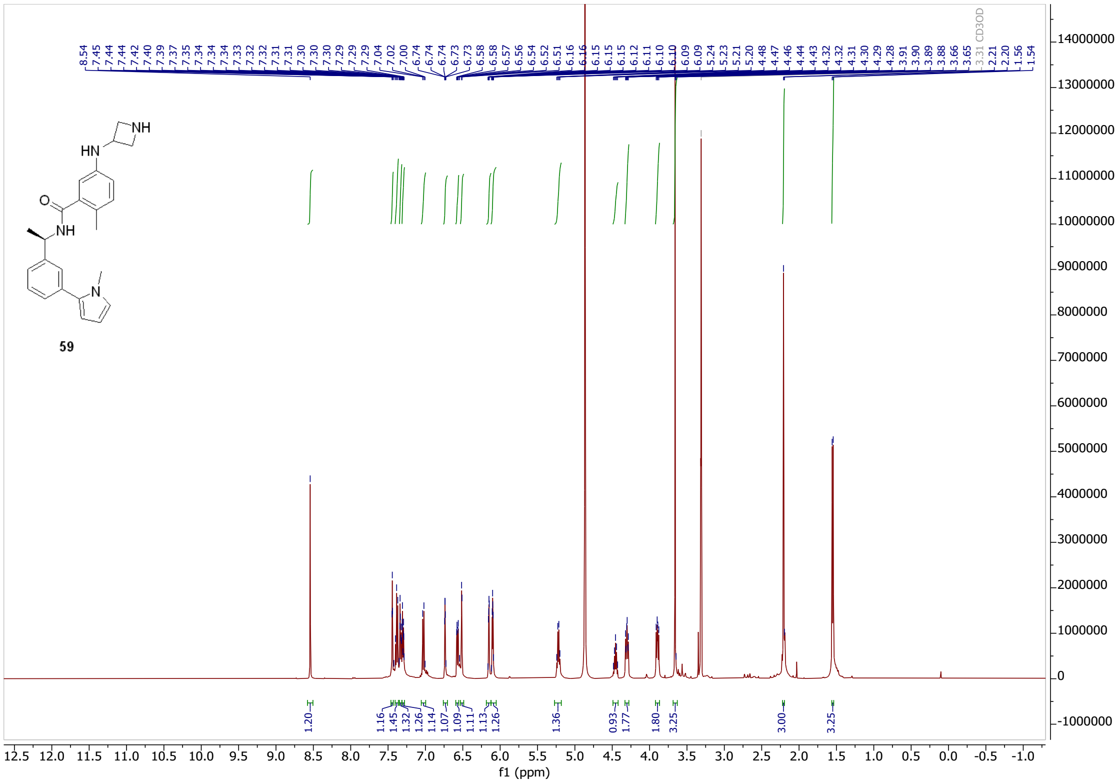

Compound **60**

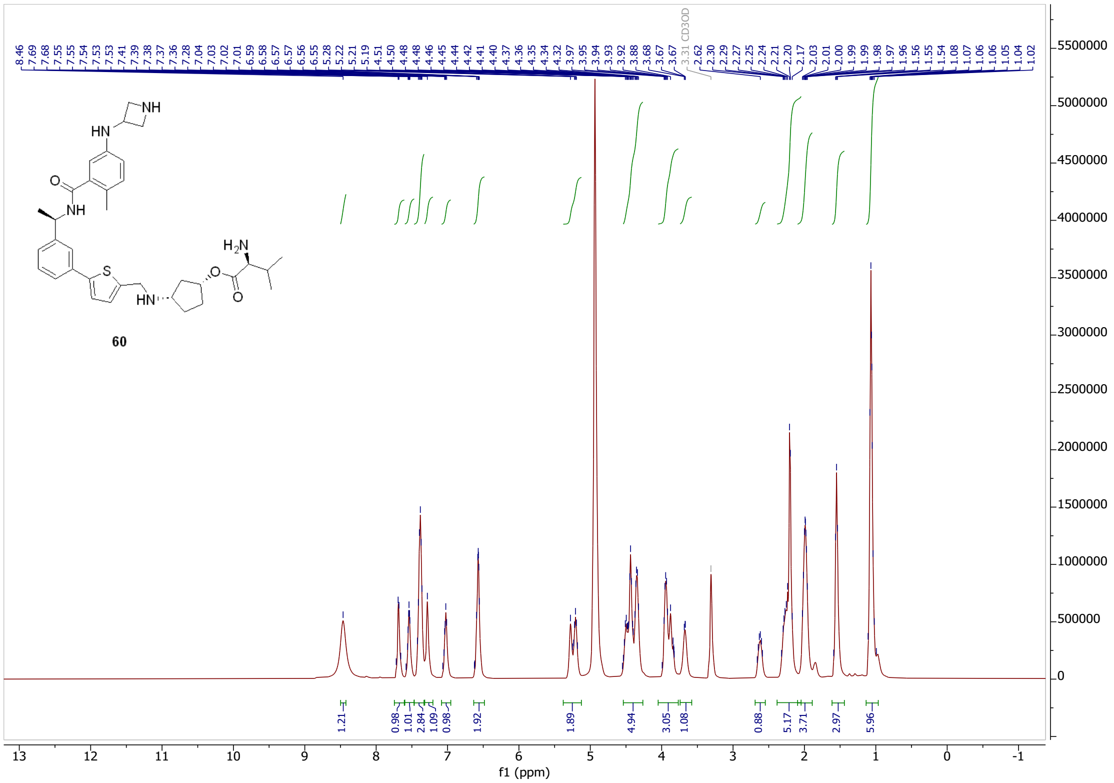

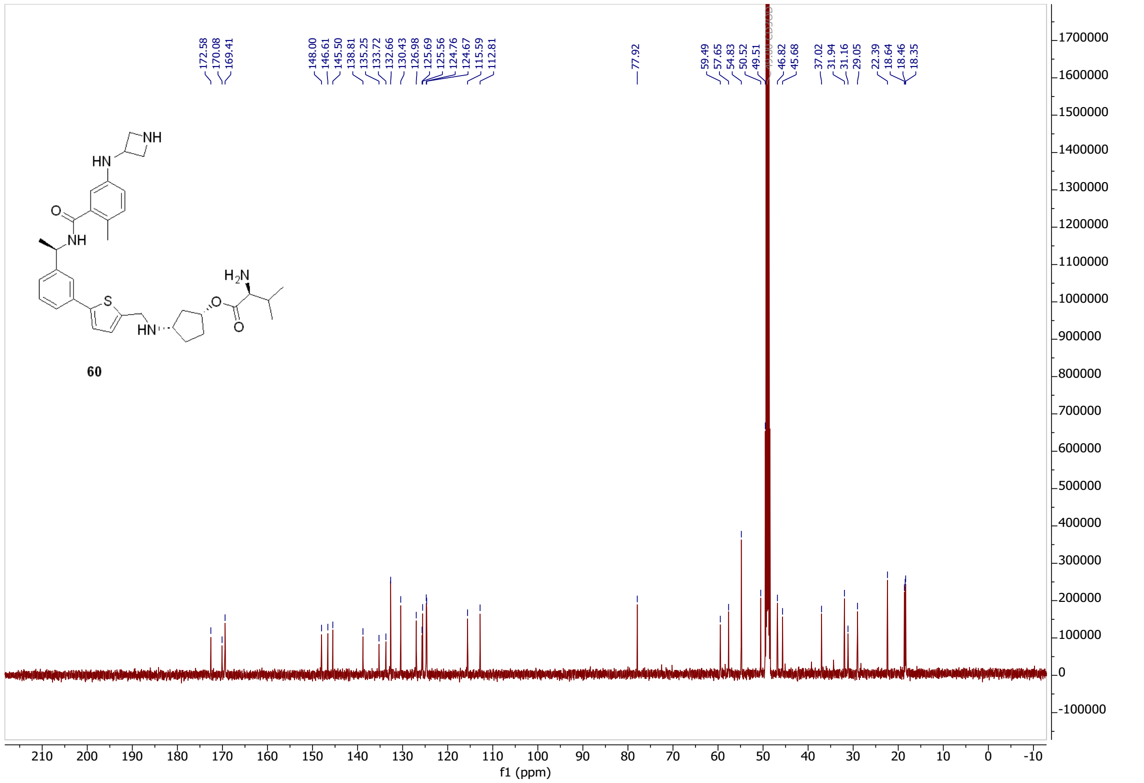

Compound **61**

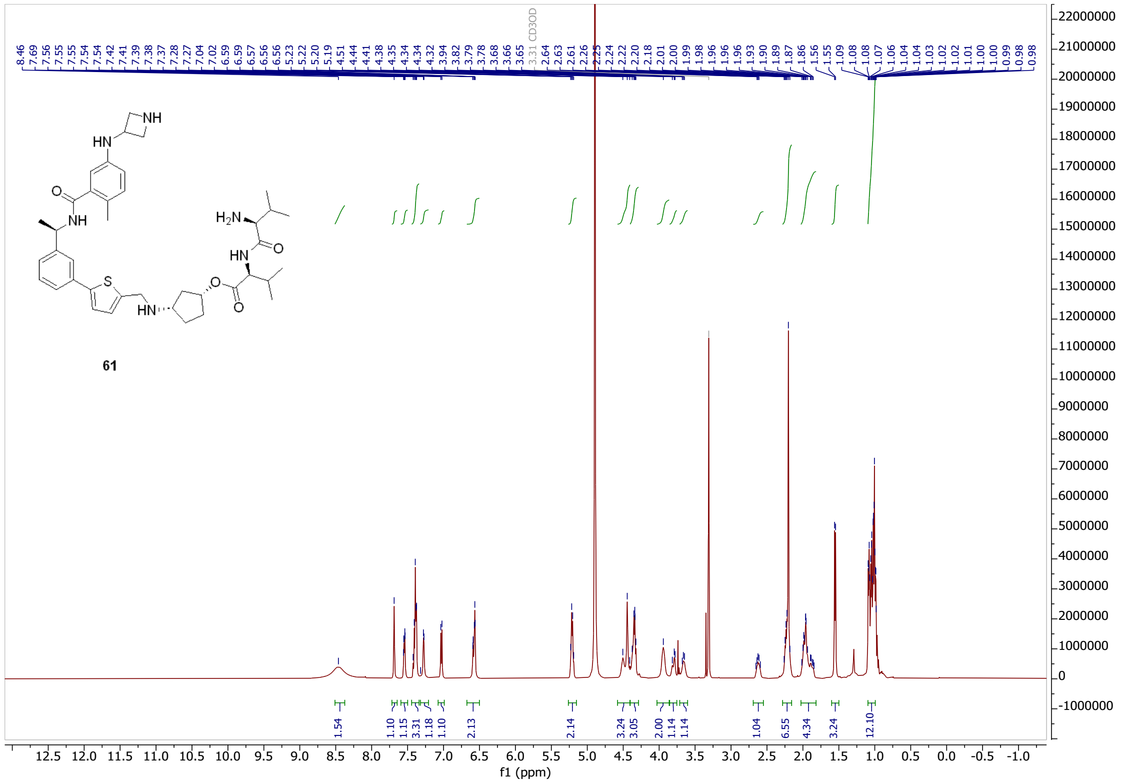

Compound **62**

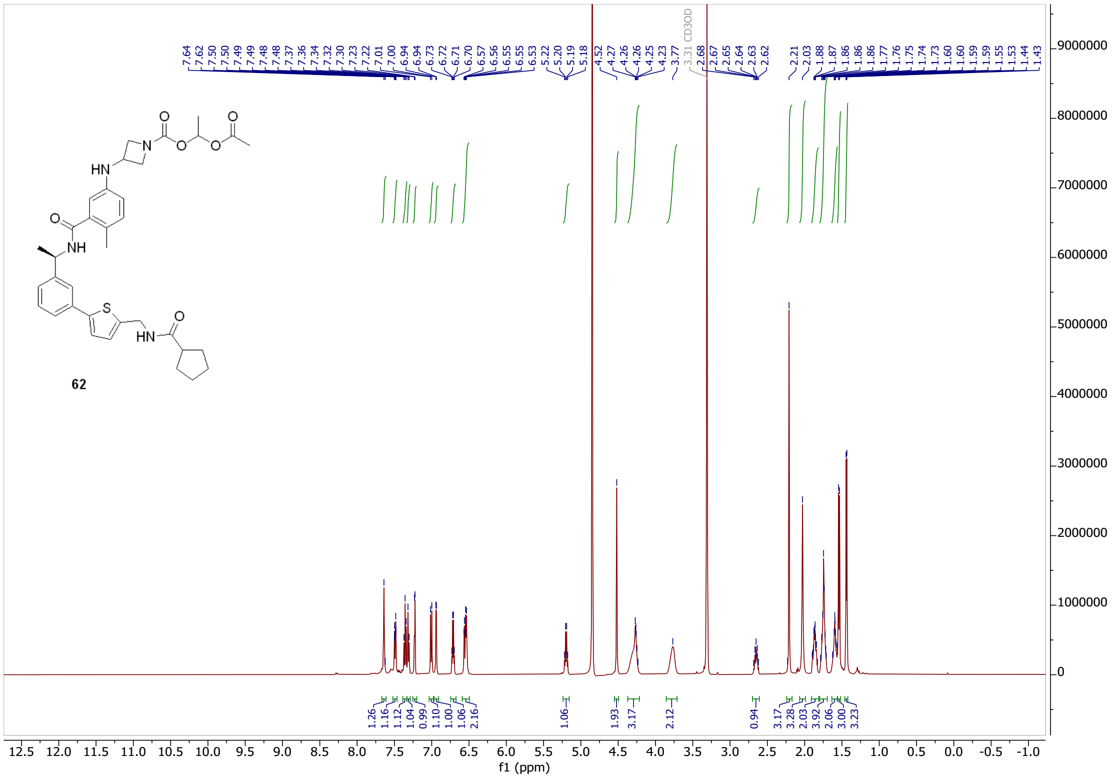

Compound **63**

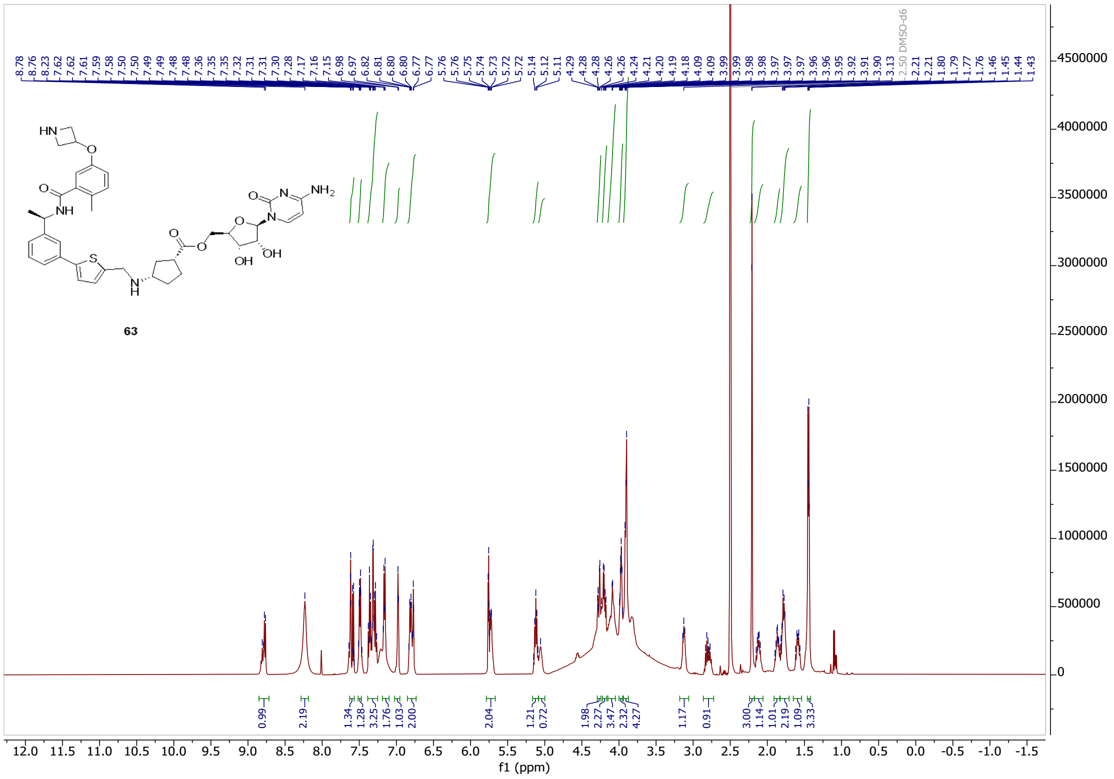

Compound **64**

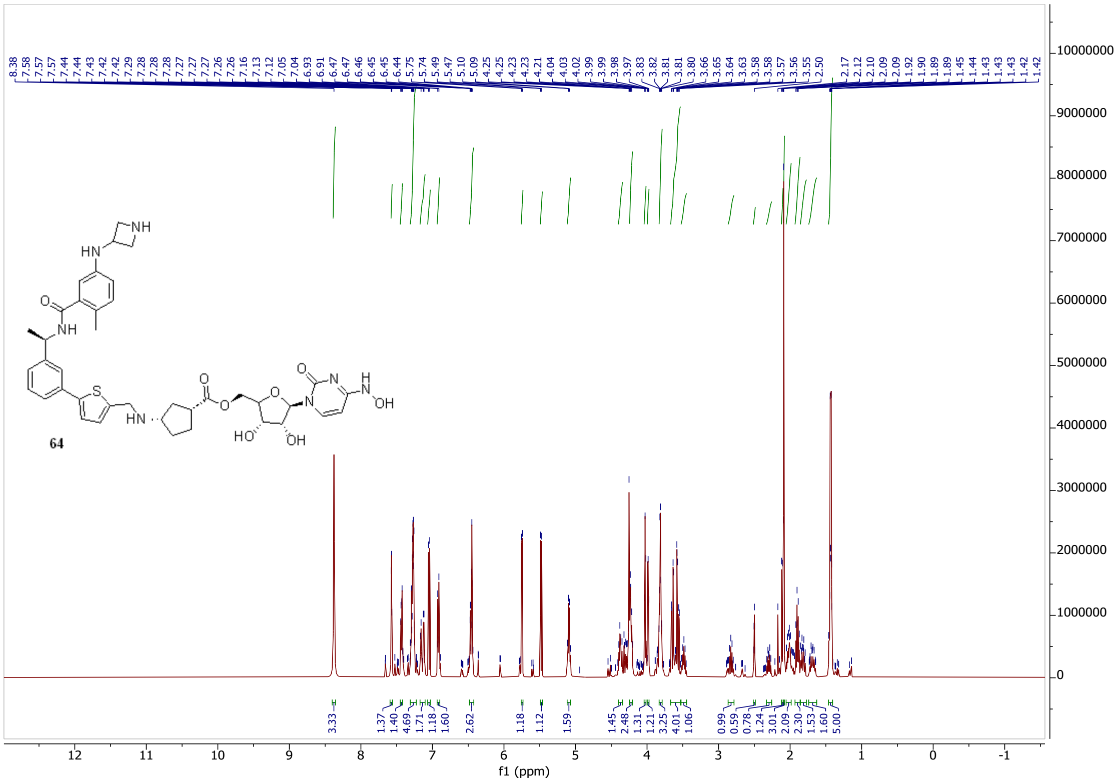

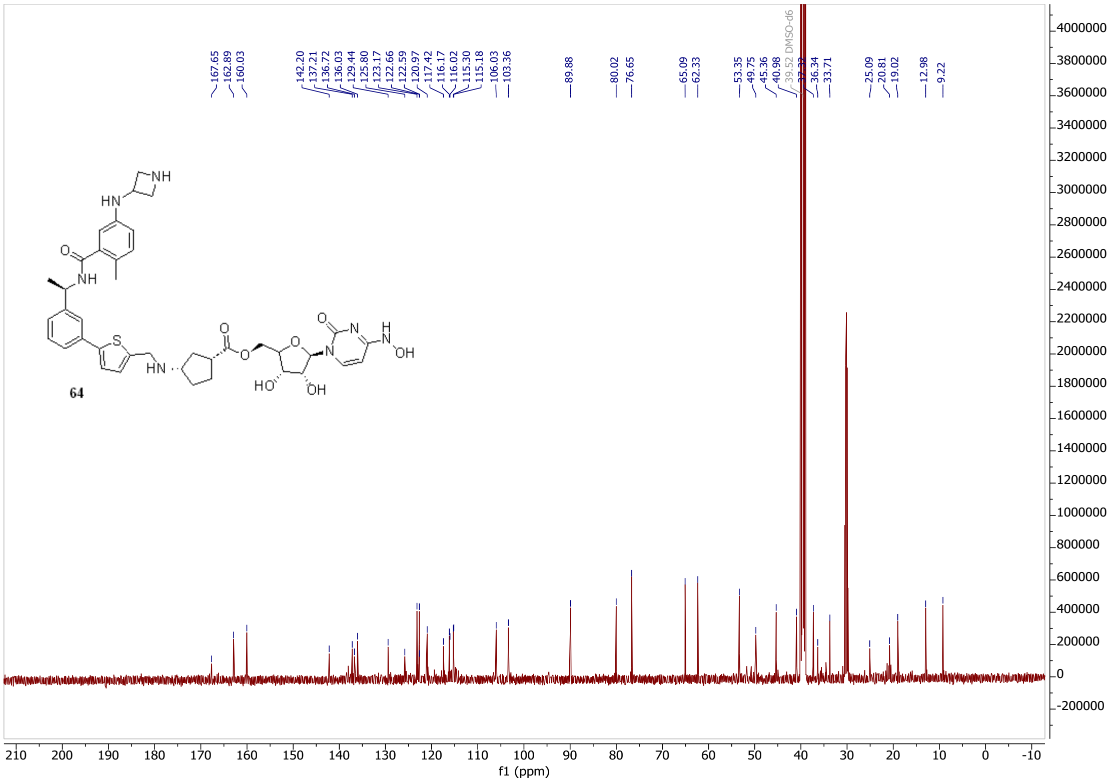

HPLC Traces

Compound **22**

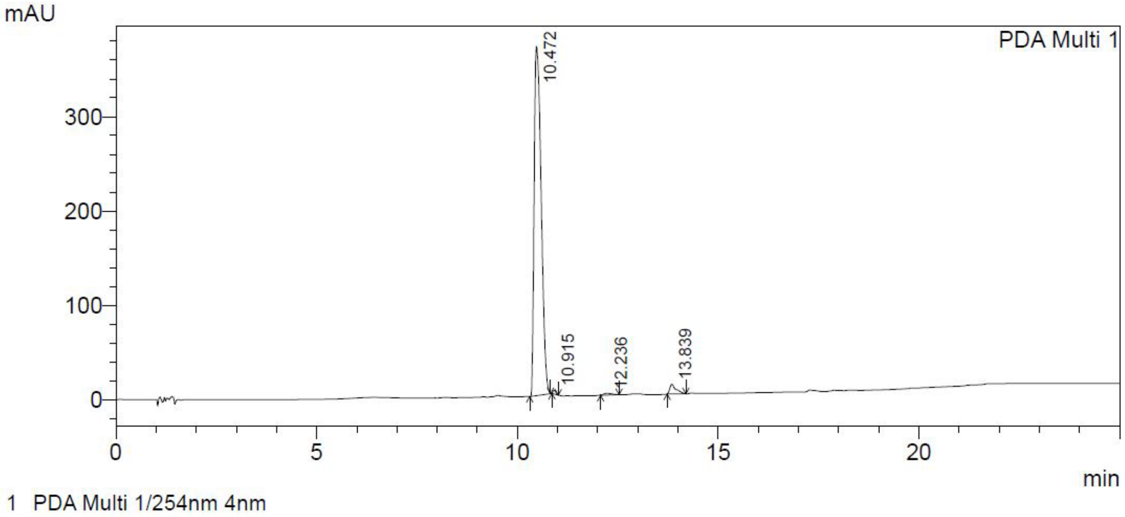

PeakTable

PDA Ch1 254nm 4nm

Peak#	Ret. Time	Area	Height	Area %	Height %
1	10.472	4443663	369942	96.540	95.792
2	10.915	20403	4161	0.443	1.077
3	12.236	26198	1766	0.569	0.457
4	13.839	112640	10322	2.447	2.673
Total		4602904	386191	100.000	100.000

Compound **23**

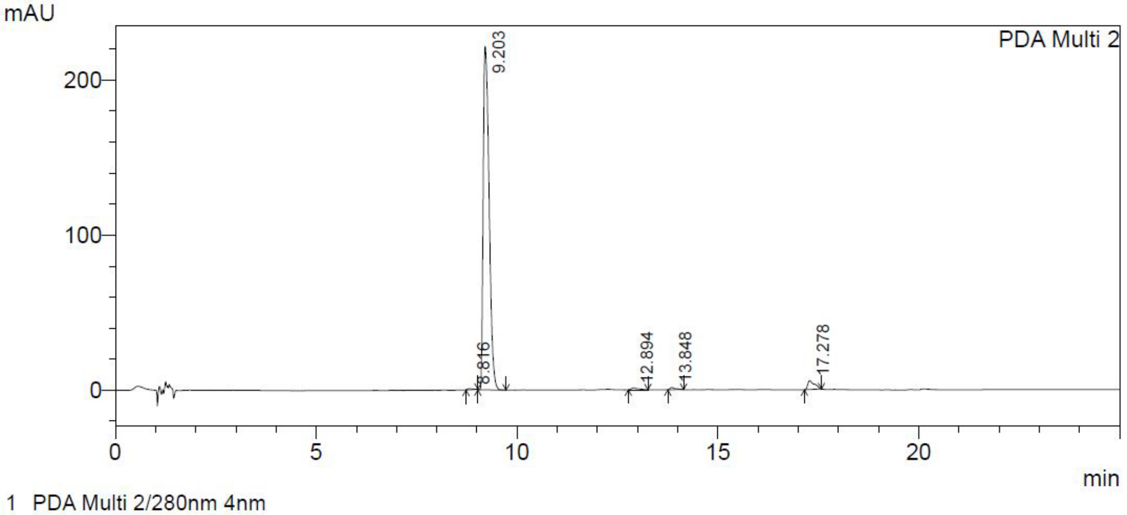

PeakTable

PDA Ch2 280nm 4nm

Peak#	Ret. Time	Area	Height	Area %	Height %
1	8.816	6140	733	0.261	0.318
2	9.203	2249718	221546	95.750	96.073
3	12.894	13706	1138	0.583	0.494
4	13.848	13439	1378	0.572	0.597
5	17.278	66579	5808	2.834	2.519
Total		2349583	230602	100.000	100.000

Compound **28**

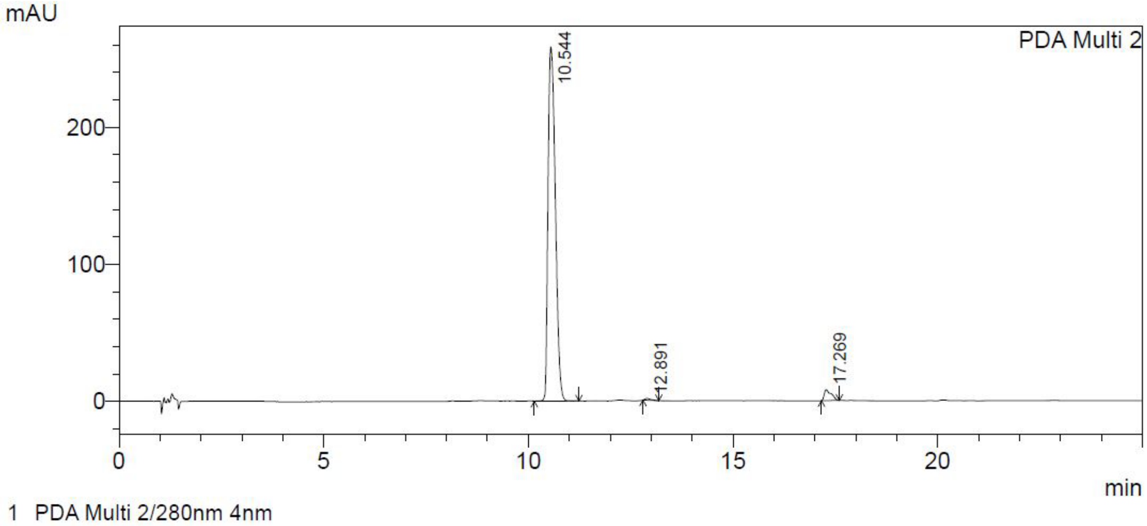

PeakTable

PDA Ch2 280nm 4nm

Peak#	Ret. Time	Area	Height	Area %	Height %
1	10.544	3190374	258541	96.736	96.548
2	12.891	13569	1271	0.411	0.475
3	17.269	94062	7972	2.852	2.977
Total		3298004	267784	100.000	100.000

Compound **32**

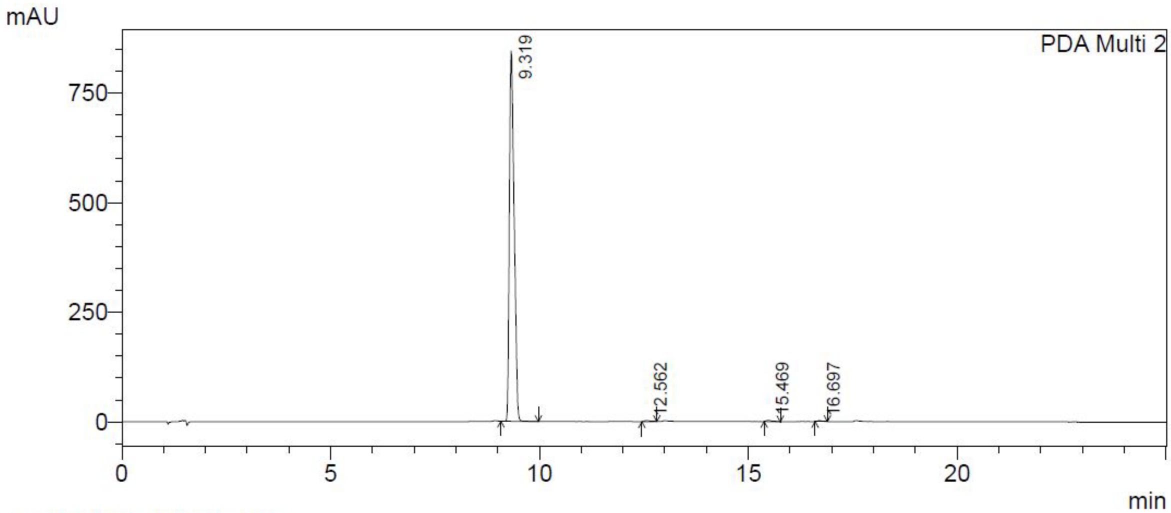

1 PDA Multi 2/280nm 4nm

PeakTable

PDA Ch2 280nm 4nm

Peak#	Ret. Time	Area	Height	Area %	Height %
1	9.319	6689914	846151	99.008	99.060
2	12.562	23262	2591	0.344	0.303
3	15.469	26560	2989	0.393	0.350
4	16.697	17225	2450	0.255	0.287
Total		6756961	854182	100.000	100.000

Compound **39**

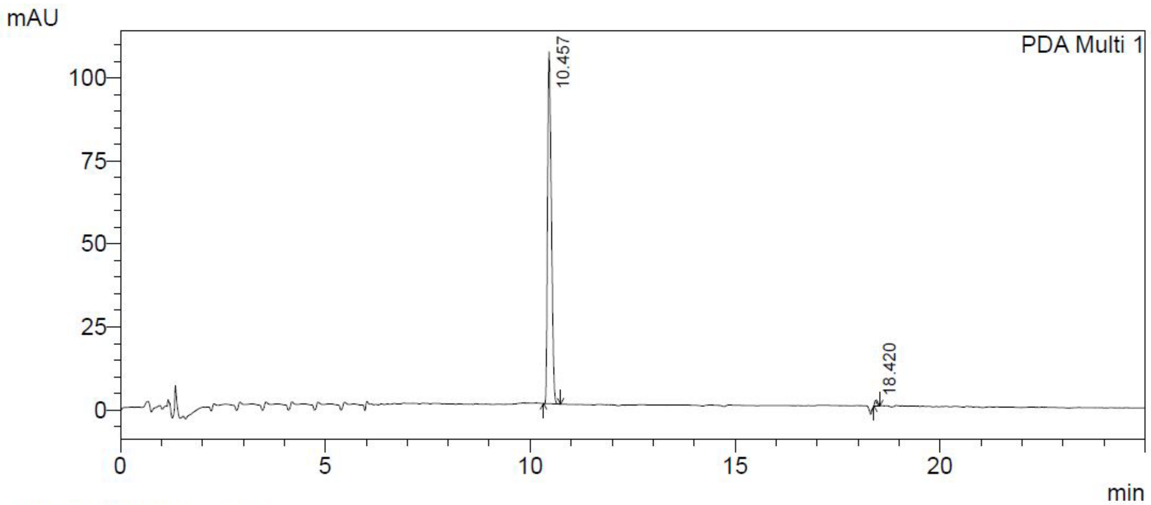

1 PDA Multi 1/254nm 4nm

PeakTable

PDA Ch1 254nm 4nm

Peak#	Ret. Time	Area	Height	Area %	Height %
1	10.457	684993	106128	99.142	98.476
2	18.420	5929	1643	0.858	1.524
Total		690923	107771	100.000	100.000

Compound **40**

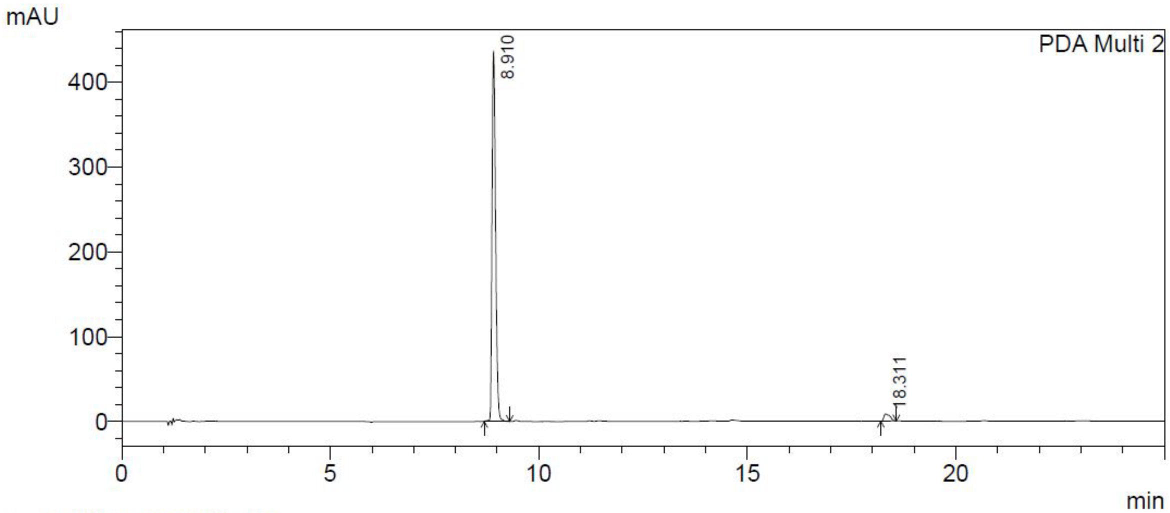

1 PDA Multi 2/280nm 4nm

PeakTable

PDA Ch2 280nm 4nm

Peak#	Ret. Time	Area	Height	Area %	Height %
1	8.910	2589963	437092	96.770	98.083
2	18.311	86450	8542	3.230	1.917
Total		2676412	445634	100.000	100.000

Compound **42**

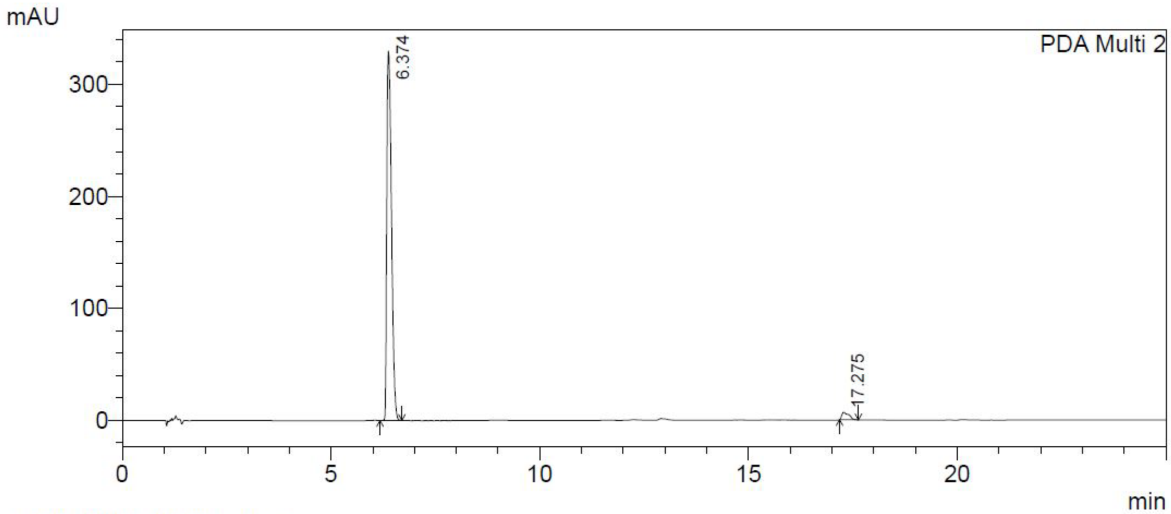

1 PDA Multi 2/280nm 4nm

PeakTable

PDA Ch2 280nm 4nm

Peak#	Ret. Time	Area	Height	Area %	Height %
1	6.374	2624053	329683	97.167	98.027
2	17.275	76506	6636	2.833	1.973
Total		2700559	336319	100.000	100.000

Compound **43**

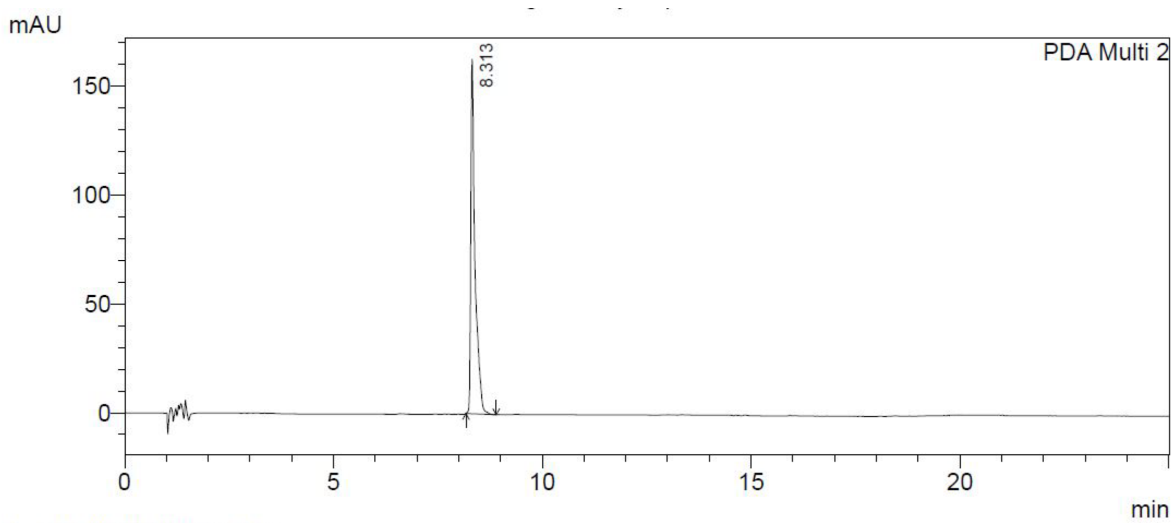

1 PDA Multi 2/280nm 4nm

PeakTable

PDA Ch2 280nm 4nm

Peak#	Ret. Time	Area	Height	Area %	Height %
1	8.313	1160728	162930	100.000	100.000
Total		1160728	162930	100.000	100.000

Compound **48**

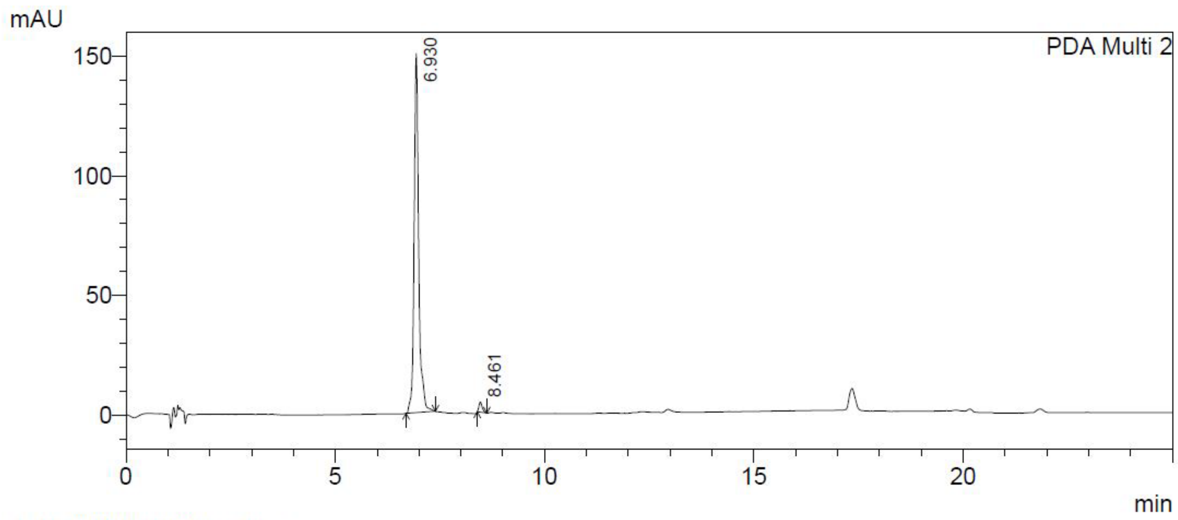

1 PDA Multi 2/280nm 4nm

PeakTable

PDA Ch2 280nm 4nm

Peak#	Ret. Time	Area	Height	Area %	Height %
1	6.930	1128678	150107	97.995	97.226
2	8.461	23096	4283	2.005	2.774
Total		1151774	154390	100.000	100.000

Compound **51**

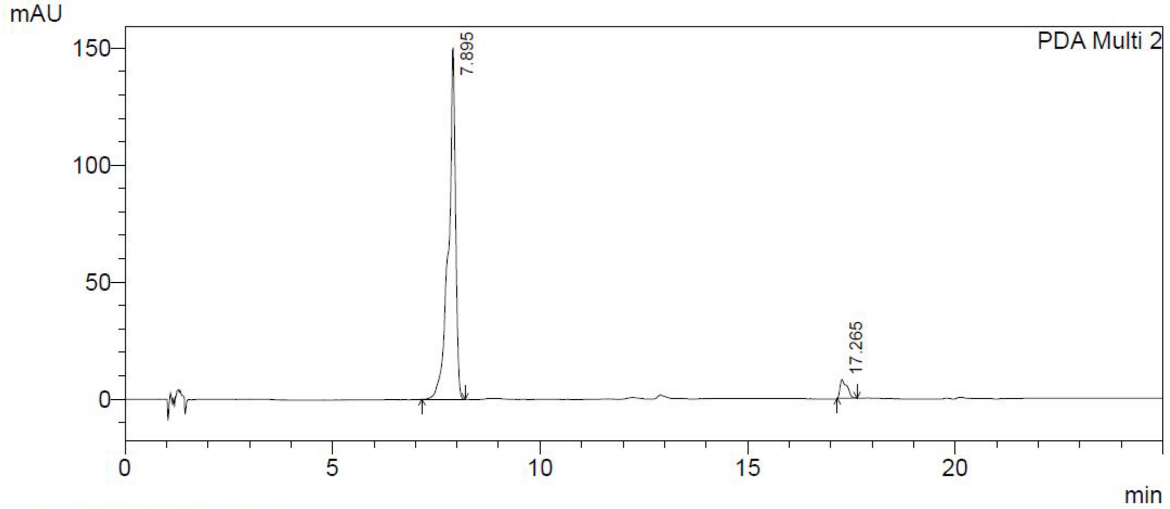

1 PDA Multi 2/280nm 4nm

PeakTable

PDA Ch2 280nm 4nm

Peak#	Ret. Time	Area	Height	Area %	Height %
1	7.895	1803156	150162	95.081	94.963
2	17.265	93278	7964	4.919	5.037
Total		1896434	158126	100.000	100.000

Compound **54**

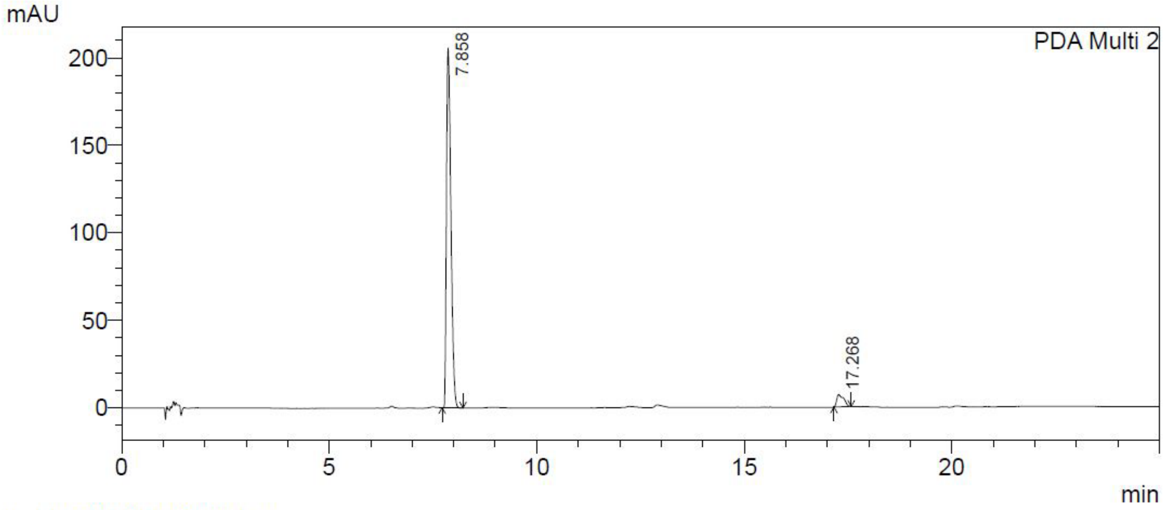

1 PDA Multi 2/280nm 4nm

PeakTable

PDA Ch2 280nm 4nm

Peak#	Ret. Time	Area	Height	Area %	Height %
1	7.858	1557810	205602	94.966	96.667
2	17.268	82569	7089	5.034	3.333
Total		1640379	212691	100.000	100.000

Compound **58**

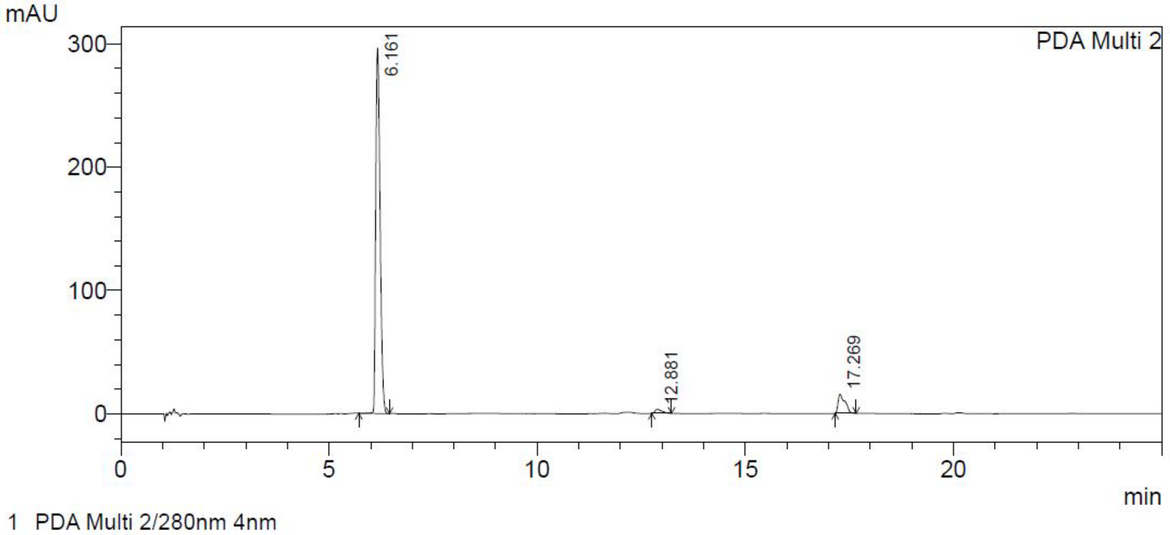

PeakTable

PDA Ch2 280nm 4nm

Peak#	Ret. Time	Area	Height	Area %	Height %
1	6.161	2134402	296567	90.702	93.929
2	12.881	40501	3520	1.721	1.115
3	17.269	178310	15648	7.577	4.956
Total		2353213	315735	100.000	100.000

Compound **62**

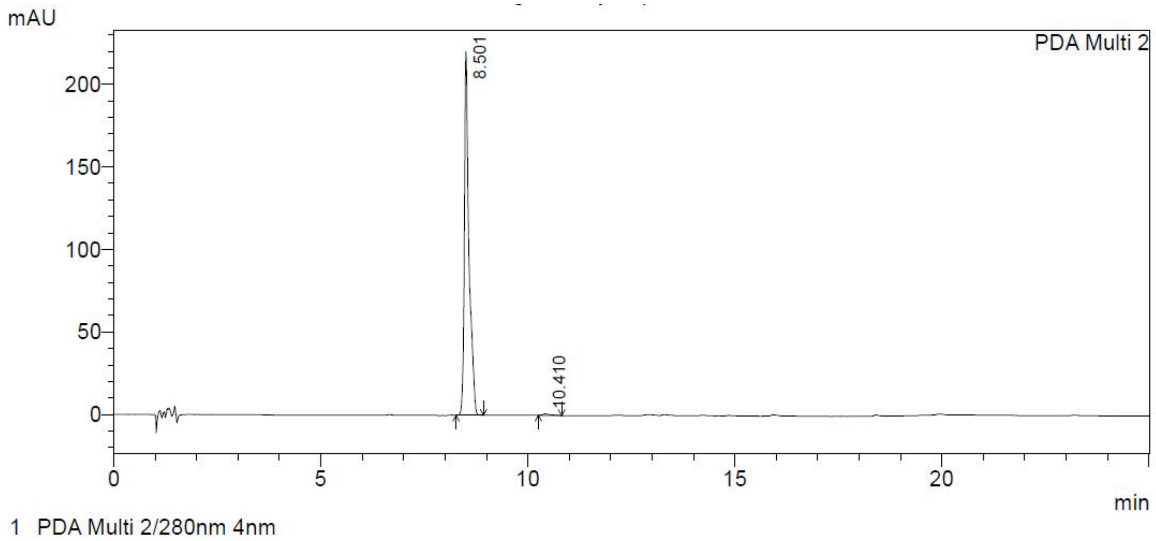

PeakTable

PDA Ch2 280nm 4nm

Peak#	Ret. Time	Area	Height	Area %	Height %
1	8.501	1707261	220283	99.469	99.583
2	10.410	9115	922	0.531	0.417
Total		1716376	221205	100.000	100.000

Compound **64**

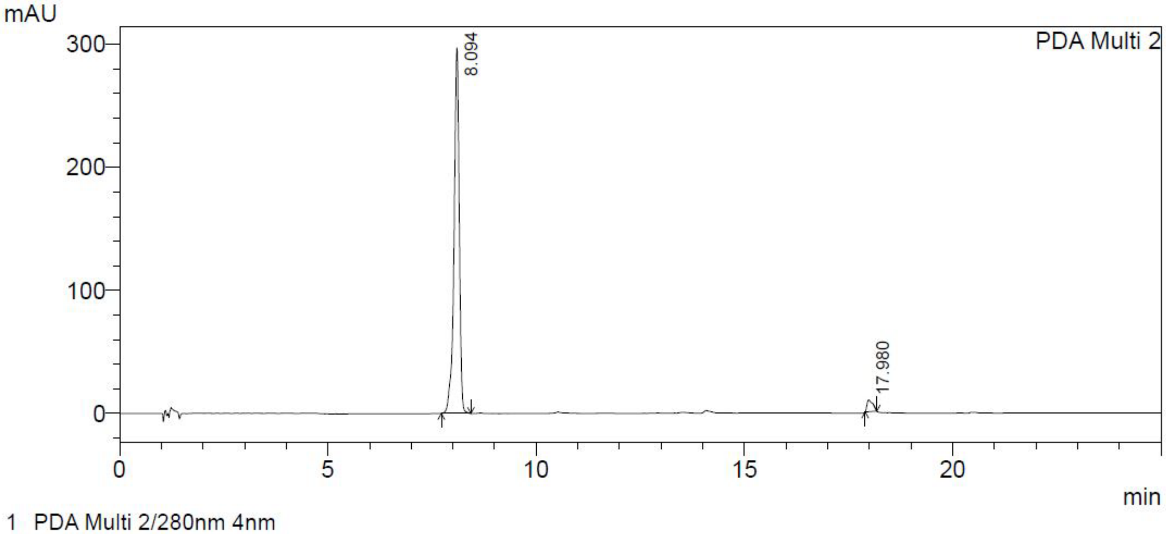

1 PDA Multi 2/280nm 4nm

PeakTable

PDA Ch2 280nm 4nm

Peak#	Ret. Time	Area	Height	Area %	Height %
1	8.094	2568273	297056	96.547	96.832
2	17.980	91862	9718	3.453	3.168
Total		2660136	306774	100.000	100.000
